# Identification and analysis of ribosome-associated lncRNAs using ribosome profiling data

**DOI:** 10.1186/s12864-018-4765-z

**Published:** 2018-05-29

**Authors:** Chao Zeng, Tsukasa Fukunaga, Michiaki Hamada

**Affiliations:** 1 AIST-Waseda University Computational Bio Big-Data Open Innovation Laboratory (CBBD-OIL), 3-4-1, Okubo Shinjuku-ku, Tokyo, 169-8555 Japan; 2 Department of Electrical Engineering and Bioscience, Faculty of Science and Engineering, Waseda University, 55N-06-10, 3-4-1 Okubo Shinjuku-ku, Tokyo, 169-8555 Japan; 3 Artificial Intelligence Research Center, National Institute of Advanced Industrial Science and Technology (AIST), 2-41-6 Aomi, Koto-ku, Tokyo, 135-0064 Japan; 4 Institute for Medical-oriented Structural Biology, Waseda University, 2-2, Wakamatsu-cho Shinjuku-ku, Tokyo, 162-8480 Japan; 5 Graduate School of Medicine, Nippon Medical School, 1-1-5, Sendagi, Bunkyo-ku, Tokyo, 113-8602 Japan

**Keywords:** Long noncoding RNA, Ribosome profiling, lncRNA, Ribosome-associated

## Abstract

**Background:**

Although the number of discovered long non-coding RNAs (lncRNAs) has increased dramatically, their biological roles have not been established. Many recent studies have used ribosome profiling data to assess the protein-coding capacity of lncRNAs. However, very little work has been done to identify ribosome-associated lncRNAs, here defined as lncRNAs interacting with ribosomes related to protein synthesis as well as other unclear biological functions.

**Results:**

On average, 39.17% of expressed lncRNAs were observed to interact with ribosomes in human and 48.16% in mouse. We developed the ribosomal association index (RAI), which quantifies the evidence for ribosomal associability of lncRNAs over various tissues and cell types, to catalog 691 and 409 lncRNAs that are robustly associated with ribosomes in human and mouse, respectively. Moreover, we identified 78 and 42 lncRNAs with a high probability of coding peptides in human and mouse, respectively. Compared with ribosome-free lncRNAs, ribosome-associated lncRNAs were observed to be more likely to be located in the cytoplasm and more sensitive to nonsense-mediated decay.

**Conclusion:**

Our results suggest that RAI can be used as an integrative and evidence-based tool for distinguishing between ribosome-associated and free lncRNAs, providing a valuable resource for the study of lncRNA functions.

**Electronic supplementary material:**

The online version of this article (10.1186/s12864-018-4765-z) contains supplementary material, which is available to authorized users.

## Background

Long non-coding RNAs (lncRNAs) are sequences longer than 200 nucleotides with no protein-coding capacity. Over 58,000 genes had been identified as human lncRNAs as of 2015 [1], and that number continues to grow [2, 3]. In contrast, only a small number of lncRNAs have been functionally annotated to date [4]. Because the majority of human lncRNAs are still interpreted as having an unknown function, identification of lncRNA functions has become a challenging problem [5].

Analysis of macromolecular lncRNA interactions has been used as an approach to conduct large-scale studies of lncRNA functions [6]. Ribosome profiling techniques adapt high-throughput sequencing methods to ribosome-protected fragment sequences, which provides a genome-wide dataset of ribosome–RNA interactions [7]. Ingolia et al. first developed ribosome profiling and applied it to studying long intergenic noncoding RNAs (lincRNAs) and reported that the majority of lincRNA fragments engaged by ribosomes represent a limited portion of different lincRNA sequences [8]. Other modified ribosome profiling techniques were applied to identify ribosome-associated lncRNAs and reduce false positives [9, 10].

Many previous studies have used ribosome profiling data to examine ribosome–lncRNA interactions, with a primary focus on detecting protein-coding signatures in lncRNAs. Hence, rigorous metrics and ignoring lncRNA characteristics can lead to underestimates of the association between lncRNAs and ribosomes. For instance, Guttman et. al defined the RRS, a ratio of counts of ribosome footprints from putative ORF to counts of ribosome footprints based on downstream sequences, to assess the sharp decrease in ribosome occupancy at the end of putative ORFs, ultimately demonstrating that lincRNAs do not produce proteins [11]. Wang et al. utilized the three-nucleotide periodicity and uniform distribution of ribosome occupancy to evaluate the translation potential of lincRNAs [12]. These two studies mainly focused on detecting lincRNAs with the ability to encode proteins while excluding any other forms or functions of ribosome-associated lncRNAs from consideration (e.g., storing ribosomes or translational regulation discussed in [13]). Ruiz-Orera et al. assessed ribosomal associations by measuring the breadth of ribosome coverage, which was defined as the number of nucleotides overlapped by Ribo-seq reads on a transcript or a transcript region [14]. This metric ignores the influences of the depth of ribosome coverage, the expression level of a transcript, and the length of a transcript with ribosomal association. Taken together, little attention has been given to ribosome–lncRNA interactions that may involve biological functions [15–17]. Efforts that focus on the identification of reliable ribosome-associated lncRNAs are insufficient.

Here, we define the term “ribosome-associated lncRNAs” as a class of lncRNAs that ribosomes associate with by sliding along regions on them or by binding to specific sites within them. In contrast, “ribosome-free lncRNAs” represent lncRNAs with little (or no) ribosomal association. Note that the term “ribosome-associated lncRNA” was frequently used in previous studies to refer to a rare fraction of lncRNAs with the predicted ability to encode peptides. By mapping ribosome profiling data to lncRNAs, we observed that an average of 39.17% (24.65–59.92%) and 48.16% (26.04–70.13%) of expressed lncRNAs interact with ribosomes in human and mouse, respectively. The protein-coding capacity remains relatively low for the total population of ribosome-associated lncRNAs compared with mRNAs. However, some evidence has emerged for the translation of ribosome-associated lncRNAs. As such, we newly present the ribosomal association index (RAI), an integrative and evidence-based tool that assigns a confidence score to a specific lncRNA representing its ribosomal associability. RAI can be applied to both tissue-specific and ubiquitous lncRNAs in combination with the tissue-specific expression metric *spec* (see “Methods”). Focusing on ubiquitously expressed lncRNAs, we used RAI * (1 - *spec*) to measure ribosomal associability. (Note that RAI*spec can be used for analyzing tissue-specific lncRNAs.) Furthermore, we apply two threshold values (the 5th and 95th percentiles of RAI * (1 - *spec*) scores) to divide the lncRNAs into “noribo-lncRNAs” and “ribo-lncRNAs.” Those lncRNAs that scored below the lower threshold are defined as “noribo-lncRNAs,” representing a subset of reliable ribosome-free lncRNAs. Conversely, lncRNAs that scored above the upper threshold are referred to as “ribo-lncRNAs,” representing a subset of high-confidence ribosome-associated lncRNAs. We show that transcript length may not be a major factor associated with ribosomal associability in lncRNAs. Moreover, we have obtained 78 human sequences (and 42 mouse sequences) that are putatively translated lncRNAs from ribo-lncRNAs, respectively. Finally, we investigated the relationship between the ribosome-associated lncRNAs and NMD and cell localization, and we conclude that RAI analyses are a valuable resource that will assist with determining the underlying lncRNA functions.

## Methods

### Data collection

We retrieved the original experimental data from NCBI GEO [18] as detailed in Additional files 1 and 2. To calculate the transcript expression level and quantify potential lncRNA–ribosomal associations, we selected ribosome profiling experiments that contained both RNA-seq and ribosome footprint (Ribo-seq) measurements. For further analysis of lncRNA–ribosomal associations, we chose a single representative dataset for each tissue or cell type according to the following three empirical criteria: (i) The mapping rates of both RNA-seq and Ribo-seq are greater than 30%; (ii) The *dist* value is less than 0.15; (iii) For a tissue/cell type represented across multiple datasets, the dataset with the lowest *dist* value, indicating that the footprint length distribution for lncRNAs is closest to that of CDSs in this dataset, is selected. Here, *dist* is a metric of the length distribution similarity between two populations of ribosome footprints that mapped to lncRNAs and CDSs, respectively.

1 $$\begin{array}{@{}rcl@{}} \text{dist}(P,Q) = \frac{1}{2}{\sum_{l\in \mathcal{L}}{|P(l) - Q(l)|}} \end{array} $$

where *P* and *Q* denote length frequency distributions of ribosome footprints that mapped to CDSs and lncRNAs, respectively, and $\mathcal {L}$ is a finite length space. This value takes a real number between 0 and 1, and larger values indicate a greater difference between these two distributions (see Additional files 3, 4 and 5). Finally, we selected ten different human datasets, which were derived from different tissues or cell types (i.e., brain, breast, fibroblasts, RPE-1, myeloma, ES, HEK293, HeLa, PC3, and U2OS). We selected eight mouse datasets, which were derived from different cell types (fibroblast, EB, and ES) and tissues (i.e., brain, hippocampi, skin, liver, and testis).

### Transcriptome

The transcriptome (consisting of mRNAs and lncRNAs) was used as a reference for mapping RNA/Ribo-seq reads based on the following considerations. First, we restricted read mapping to annotated transcripts to avoid the identification of novel transcripts. Second, mapping reads to a genome is a complicated problem as the mapping rate is sensitive for short reads and those reads spanning splicing junctions. Thus, we downloaded genomic sequences and gene annotation files from GENCODE [2] and then utilized custom Python scripts to generate transcriptome sequences (see Additional file 6). By excluding lncRNAs that are derived from known protein coding genes, we finally obtained 27,545 and 14,609 lncRNAs for human and mouse, respectively, which primarily represent lincRNA and antisense RNA sequences (see Table 1).

**Table 1:** Long non-coding RNAs used in this study

Biotype	Human	Mouse
LincRNA	13245	6473
Antisense	10980	3612
TEC	1072	2759
Sense_intronic	984	294
Retained_intron	517	294
Processed_transcript	368	980
Sense_overlapping	310	50
3prime_overlapping_ncRNA	34	3
Pseudogene	20	24
Bidirectional_promoter_lncRNA	11	118
Non_coding	3	0
Macro_lncRNA	1	2
Total	27545	14609

See https://www.gencodegenes.org/gencode_biotypes.html for the details on transcript biotype

### Alignment and quantification

RNA/Ribo-seq reads were mapped to the transcriptome using Bowtie2 [19] with the *–very-sensitive-local* option. Cutadapt [20] was used to trim adapter sequences from reads if the adapter sequence was described in the literature. Additionally, we performed a local read alignment to remove adapter sequences from one or both ends of the alignment. The Ribo-seq reads were produced by a strand-specific protocol, which means reads from 5 ^′^ to 3 ^′^ are mostly mapped to the transcript sense strand. This helps to determine whether reads were sequenced from the protein-coding transcript or the antisense transcript on the opposite strand. For each read, we allowed a maximum of 100 distinct alignments to take into account the high sequence similarity among transcript variants of the same gene locus or among transcripts with repetitive elements. Additional file 7 shows the details of the software parameters used in this procedure.

The transcript expression value RPKM (reads per kilobase per million mapped reads) was pre-computed from RNA-seq data using RSEM v1.2.31[21]. To quantify one Ribo-seq read that mapped to *N* (1≤*N*≤100) different locations, we defined a metric *w*(*i*) to represent the fraction of mapped reads assigned to the *i*-th location (1≤*i*≤*N*).

2 $$\begin{array}{@{}rcl@{}} {w(i)} = \frac{\text{RPKM}(i)}{\sum_{n=1}^{N}{\text{RPKM}(n)}} \end{array} $$

where RPKM(*i*) is the expression value for the transcript referring to the *i*-th location.

### Expressed transcripts and tissue specificity

Although most previous studies are based on quantitative data over a single representative transcript for each gene, we used RSEM to estimate the abundance of total known transcript variants from RNA-seq data, defined by an expression threshold of 1 (i.e., ≥1 RPKM) for the purpose of identifying expressed transcripts [22, 23]. Where not otherwise specified, the following analyses were based on sets of expressed transcripts.

For a transcript, to measure the expression tissue specificity, we count the number (*x*) of tissues/cell types in which this transcript is expressed and transform it to a scale from 0 (ubiquitous) to 1 (specific) as follows:

3 $$\begin{array}{@{}rcl@{}} \text{spec}= \frac{M - x}{M - 1} \end{array} $$

where *M* is the total number of tissues and cell types used in this study. The *spec* metric is consistent with the *counts* metric mentioned in [24].

### Ribosome density to distinguish ribosome-associated and ribosome-free lncRNAs in a single dataset

**Fig. 1:**
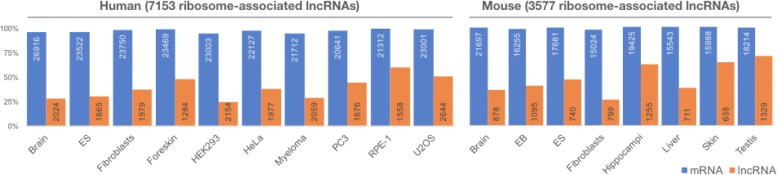
The percentages of expressed mRNAs (blue) and lncRNAs (orange) that are associated with ribosomes across multiple tissues or cell types. For the human datasets, 94.73–99.51% of mRNAs and 24.65–59.92% of lncRNAs were associated with ribosomes. For the mouse datasets, 95.99–99.42% of mRNAs and 26.04–70.13% of lncRNAs were associated with ribosomes. In summary, 7153 and 3577 lncRNAs were identified to be associated with ribosomes in at least one dataset. The ribosome association was defined based on ribosome density (see “Methods”). The number of expressed mRNAs or lncRNAs is shown in each bar

To measure the extent to which ribosomes are associated with a transcript or a region of a transcript, we used ribosome density, which is calculated as

4 $$\begin{array}{@{}rcl@{}} \text{ribosome\_density}(i,j;T)= \frac{\text{ribo}(i,j)}{\text{RPKM}(T) \cdot {|j-i+1|}} \end{array} $$

where *T*=*t*_1_...*t*_*n*_ is a transcript of length *n*, ribo(*i*,*j*) is the number of Ribo-seq reads mapped on the substring *T*(*i*,*j*)=*t*_*i*_...*t*_*j*_ (1≤*i*≤*j*≤*n*), and RPKM(*T*) is the expression value of *T*. Thus, ribosome_density(1,*n*;*T*) represents the density of ribosome occupancy over the whole transcript *T*. In general, a ribosome will dissociate from mRNA once a stop codon is encountered, which makes the area downstream of the stop codon (the 3 ^′^ UTR), a ribosome-free region and thus a suitable reference region for detecting ribosome-associated signals. To obtain a significant ribosome-associated lncRNA, we further derived an empirical distribution of ribosome density scores from 3 ^′^ UTRs and then applied a 90th percentile cut-off value of ribosome density scores from 3 ^′^ UTRs in order to distinguish between ribosome-associated and ribosome-free lncRNAs (see Fig. 3a). The rationale for choosing this seemingly less stringent cut-off value is that it (i) may enable the detection of ribosome rescue in 3 ^′^ UTRs [25] and (ii) guarantees that the majority (i.e., > 90%) of mRNAs that are associated with ribosomes and produce proteins are identified as expected [26] (see Fig. 1).

### Ribosomal association index (RAI) defines ribo-lncRNAs and noribo-lncRNAs across multiple datasets

For each lncRNA, we applied the newly proposed ribosomal association index (RAI) to quantify ribosome associability.

5 $$\begin{array}{@{}rcl@{}} \text{RAI} = \frac{\sum_{i=1}^{M}{x(i) \cdot y(i)}}{\sum_{i=1}^{M}{x(i)}} \end{array} $$

where *M* is the number of independent experiments; *x*(·) is the indicator function of transcript expression, that is, *x*(*i*)=1 if the lncRNA is expressed in the *i*-th experiment and 0 otherwise; and *y*(·) denotes the ribosomal association sign function, that is, *y*(*i*)=1 if the ribosomal association was supported by the *i*-th experiment and − 1 otherwise. Here, a continuous value of *y*(*i*) will provide more information about the ribosomal association. However, it is difficult to directly compare the ribosomal association across different datasets by using ribosome density, which is normalized to transcript abundance in each dataset.

**Fig. 2:**
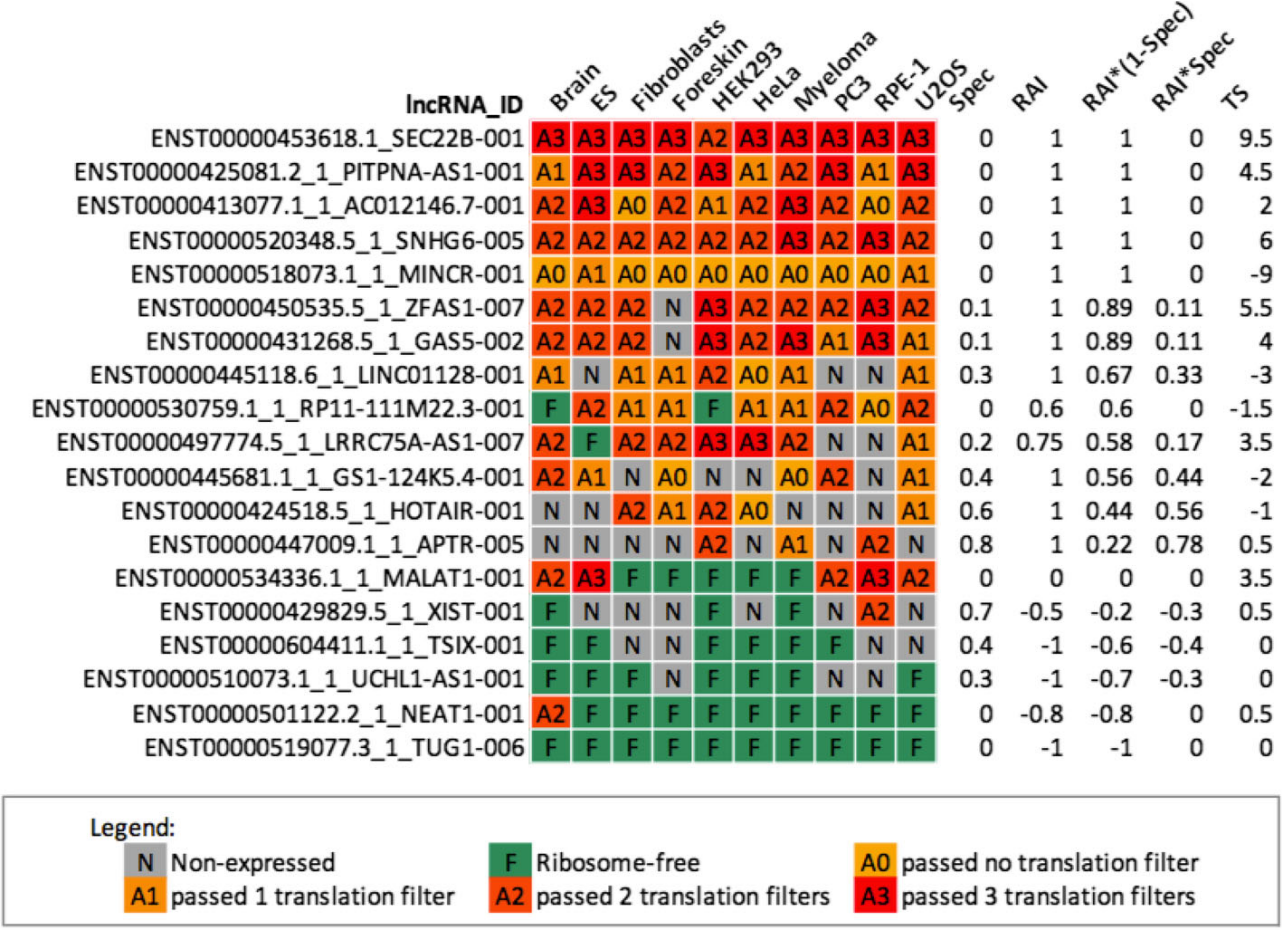
The ribosomal association index (RAI) enables an integrative analysis of ribosome associability of lncRNAs across multiple independent datasets. The table summarizes ribosomal association and translation for selected human lncRNAs. Rows represent lncRNAs, while colored columns denote datasets. For each lncRNA, “N (gray)” and “F (green)” cells correspond to unexpressed and ribosome-free lncRNAs, respectively. “A0” ∼“A3” cells represent the lncRNA containing a putative ORF that passed 0–3 coding filters. The last four columns are statistics that describe the corresponding lncRNAs. Spec is the transcript expression specificity, ranging from 0 (ubiquitous) to 1 (specific). For a lncRNA, RAI is the ribosomal association index across datasets in which this lncRNA is expressed, ranging from -1 (ribosome-free) to 1 (ribosome-associated). RAI * (1 - *spec*) is a metric to measure the confidence of ribosomal association for a lncRNA that has a broad expression, ranging from -1 (lncRNA was observed as ribosome-free in most datasets) to 1. Conversely, RAI * spec can be used to select ribosome-associated or ribosome-free lncRNAs from the population of tissue-specific lncRNAs. TS can be used with RAI * (1 - *spec*) to filter the putatively translated lncRNAs. (See Additional file 8 for the complete list of human and mouse lncRNAs)

Furthermore, we used RAI * (1 - *spec*) to assign a more confident score of ribosome associability based on multiple pieces of experimental evidence. The RAI * (1 - *spec*) score can range between 1, for ribosome-associated lncRNAs, and -1, for the ribosome-free lncRNAs (see Fig. 2 and Additional file 8).

### The putative ORF in lncRNAs

For lncRNAs, putative ORFs with lengths ≥ 30nt (including the stop codon) were considered to analyze their coding potential. A putative ORF is a continuous sequence of trinucleotides starting with an ATG trinucleotide and ending with TGA, TAA, or TAG.

### Coding potential assessment

#### Fragment length organization similarity score

The fragment length organization similarity score (FLOSS) was computed as formulated and presented by [27]. Footprints derived from translating ribosomes are expected to have a specific length distribution. Thus, the idea behind the FLOSS analysis is to compare the histogram distributions of footprint lengths between a given transcript and the reference (i.e., CDSs), in which ribosomes are considered to translate proteins. To maintain the consistency of metrics of coding potential, we transformed the original FLOSS score to 1 - FLOSS. Thus, the transformed FLOSS (called FLOSS hereafter) value range is from 0 (non-translated) to 1 (high possibility of translating).

#### Ribosome release score

For a previously defined putative ORF of a lncRNA or a CDS, the ribosome release score (RRS) was calculated according to the description in [11]. A transcript undergoing translation tends to show ribosome coverage over the majority of an ORF, and thus the ribosome density over ORFs ends sharply at the translation termination site. Guttman et al. developed the ribosome release score (RRS) based on the drop signal at the translation termination site to detect the translation event [11]. Here, the RRS value was scaled to range from 0 to 1.

#### Framescore

As the ribosome moves three nucleotides in each step along each ORF during protein synthesis, the three-base periodicity can be represented by the frame distribution, which displays the frequency of Ribo-seq reads (from the 5 ^′^ end of each read) in each frame. If the majority of lncRNAs also encode peptides, three-base periodicity would be expected in most of their putative ORFs. Note that the different experiments and different methods of processing reads may affect the shape of the frame distributions. Fortunately, the frame distribution of CDSs provides a good reference for the differentiation of ORFs between active and inactive translation. We proposed the Framescore to measure the dissimilarity in terms of frame distribution, which is the proportion of 5 ^′^ ends of Ribo-seq reads mapped to all three frames. Here, *Q* is the frame distribution of Ribo-seq reads among all CDSs undergoing ribosomal translation, and *P* represents the frame distribution of reads in a (putative) ORF from a transcript. Framescore was used to calculate the Kullback–Leibler divergence from *P* to *Q* as

6 $$\begin{array}{@{}rcl@{}} \text{Framescore}(P,Q) = {\sum_{i=1}^{3}{P(i)\log\frac{P(i)}{Q(i)}}}. \end{array} $$

The difference between Framescore and ORFscore which is the other triplet phasing metric [28], is that ORFscore supposes footprints derived from translating ribosomes will be predominantly mapped to frame one and frame two. However, Framescore uses the mapping results onto CDSs as the reference to obtain a more stable performance.

#### Translation score

Taken together, we applied these three coding metrics (FLOSS, RRS, and Framescore) to assess the ability of each putative lncRNA ORF to encode a peptide. For each coding metric, a cut-off was generated such that 90% of mRNAs can be identified as having coding ability according to this threshold, which was then applied to lncRNAs. To integrate these three filtering results, we developed the translation score (TS) to evaluate the coding potential for a specific lncRNA across multiple datasets.

7 $$\begin{array}{@{}rcl@{}} \text{TS} = {\sum_{i=1}^{N}{w(\alpha(i))}} \end{array} $$

where *N* is the number of datasets in which the transcript is identified as ribosome-associated. In the *i*-th dataset, *α*(*i*) is a translation level function ranging from 0 to 3, indicating the maximum number of coding filters passed for a putative lncRNA ORF. While *w*(·) is a function that assigns the weight for each translation level (0, 1, 2, and 3 correspond to weights of -1, -0.5, 0.5, and 1, respectively). Finally, for a given lncRNA, TS is a weighted sum function, with a positive value indicating translation, and a negative value indicating no translation.

### Mass spectrometry data

Peptide sequences derived from mass spectrometry data were downloaded from sORFs.org [29]. Peptide sequences aligned to protein coding transcripts (by tBlastn [30]) were removed, then the remaining peptides were aligned to lncRNAs.

### Sequence conservation

PhyloP scores, which measure base-wise evolutionary conservation from multiple alignments, were downloaded from GENCODE [2]. Positive phyloP scores represent slower evolution than expected (in other words, conserved), and vice versa.

### Nonsense-mediated decay (NMD) and cellular localization analysis

For the NMD analysis, we computed the fold change of RNA-seq expression levels from the control sample to those from the UPF1 knockdown sample. Here, UPF1 is one of the major NMD factors, and interfering with the expression of UPF1 is expected to cause increased expression levels of NMD-targeted transcripts. RNA-seq data from HeLa cells were downloaded from NCBI GEO (GSE86148) [31].

For the cellular localization analysis, cells were first separated into cellular fractions before the extraction of RNA. We calculated the fold change of RNA-seq data from the cytoplasmic fraction to that from the nuclear fraction of HeLa cells. RNA-seq data from the nucleus (ENCSR000CPQ) and the cytoplasm (ENCSR000CPP) were download from ENCODE [32].

We applied the same procedure to calculate the fold change for the NMD analysis and the cellular localization analysis. Reads mapped to tRNAs, rRNAs, snoRNAs, or miRNAs were first removed. For the remaining reads, their first 15 nucleotides with low sequencing qualities were trimmed by Cutadapt [20]. Trimmed reads were mapped to the transcriptome by Bowtie [33]. Transcript expression values were calculated by RSEM v1.2.31 [21]. Differential expression analysis was performed using EBSeq [34] to obtain the posterior fold change for each transcript.

## Results

### A large fraction of expressed lncRNAs are associated with ribosomes

**Fig. 3:**
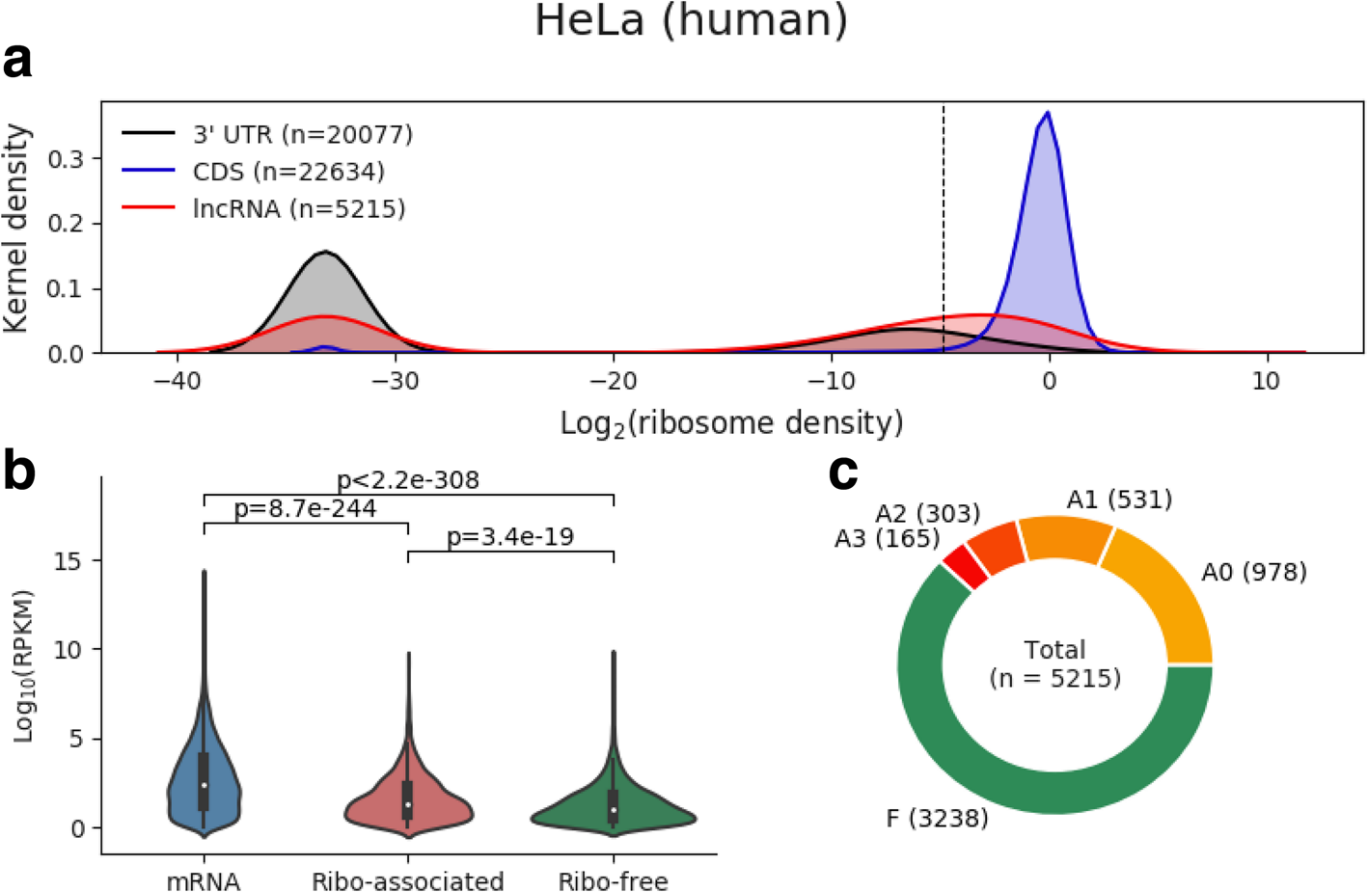
The discrimination of ribosome-associated and ribosome-free lncRNAs by ribosome density in the HeLa dataset. **a** Kernel density distribution of ribosome density (log_2_ scale) for 3 ^′^UTRs (gray), CDSs (blue), and lncRNAs (red). The vertical dashed line corresponds to the 90th percentile of the ribosome density scores for 3 ^′^ UTRs, which is used as the cut-off to distinguish between ribosome-associated lncRNAs and ribosome-free lncRNAs. Those lncRNAs to the right of this cut-off (including the cut-off itself) are identified as ribosome-associated lncRNAs; the rest are ribosome-free in this study. Note that transcripts or regions without any mapped Ribo-seq read correspond to a peak near -33 (owing to the addition of a pseudo value of 10e-10 prior to log transformation). **b** Violin plot of the expression levels (RPKM, log2 scale) of mRNAs as well as ribo-associated and free lncRNAs. The *p*-values correspond to two-sample *t*-tests. **c** Classification of lncRNAs by using FLOSS, RRS, and Framescore as filters to assess the coding potential for each ribosome-associated lncRNA. “F” means ribosome-free, “A0” means no coding filter has been passed, “A1”, “A2”, and “A3” denote that one, two, and three passed translation filter(s), respectively. (See Additional file 9 for the other datasets)

To identify ribosome-associated lncRNAs in each dataset, we first calculated the ribosome density (i.e., the number of ribosomes per unit length of transcript) for each lncRNA and further derived the empirical distribution of ribosome density values from 3 ^′^ UTRs. Then we adopted a cut-off value at the 90th percentile of the ribosome density values for 3 ^′^ UTRs. The rationale for choosing this cut-off value is that it guarantees that the majority (i.e., > 90%) of mRNAs that are associated with ribosomes and produce proteins are identified as expected [26] (see Fig. 3a and Additional file 9). Finally, a transcript with ribosome density greater than or equal to the cut-off value was defined as ribosome-associated and was otherwise defined as ribosome-free. For expressed mRNAs, an average of 97.36% (94.73–99.51%) and 98.30% (95.99–99.42%) of them were observed to interact with ribosomes in human and mouse, respectively. This is in agreement with the fact that mRNAs serve as protein-coding transcripts associated with ribosomes. Surprisingly, we found that an average of 39.17% (24.65–59.92%) of human-expressed lncRNAs and an average of 48.16% (26.04–70.13%) of mouse-expressed lncRNAs were also associated with ribosomes (see Fig. 1 and Additional file 10). In total, 7,153 and 3,577 lncRNAs were identified as associated with ribosomes in at least one human and mouse dataset, respectively. We also determined that ribosomal association was more difficult to detect among low-expression transcripts than among highly expressed ones, but this was not observed among all datasets (see Fig. 3b and Additional file 9). Despite the differences between the experiment samples, which may affect the expression level and the ribosomal association of lncRNAs, a substantial fraction of lncRNAs were observed to interact with ribosomes over all human and mouse ribosome profiling experiments.

### Analysis of the coding potential for ribosome-associated lncRNAs based on Ribo-seq

To further examine whether the ribosome-associated lncRNAs encode peptides, we first defined the putative lncRNA ORFs (see “Methods”), and then assessed the coding potential of their putative ORFs based on the following considerable characteristics for translating ORFs. (i) FLOSS (fragment length organization similarity score) was used to compare the length distributions of footprints from CDSs with the surveyed lncRNAs; (ii) RRS (ribosome release score) was used to measure the drop signal of footprints at the translation termination sites; (iii) Framescore, which was developed in this study, was used to measure the three-nucleotide periodicity. Note that such characteristics are measured by analyzing Ribo-seq reads across a given transcript (see “Methods” for detailed description of FLOSS, RRS, and Framescore).

**Fig. 4:**
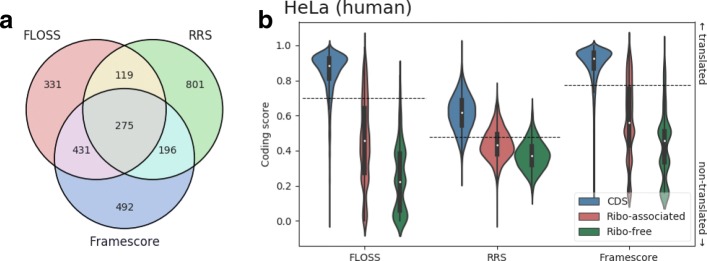
Analysis of coding potential by using FLOSS, RRS, and Framescore on the HeLa dataset. **a** Venn diagram of putative ORFs in ribosome-associated lncRNAs evaluated by three coding filters (FLOSS, RRS, and Framescore). **b** Comparisons of the coding potential among CDSs (blue) and putative ORFs of ribosome-associated (red) or ribosome-free lncRNAs (green) for FLOSS, RRS, and Framescore, respectively. Based on these three coding metrics, we generated three cut-offs (the 10th percentiles represented as horizontal dashed lines) from CDSs to independently filter translation events for lncRNAs. For a coding filter of FLOSS, RRS, or Framescore, lncRNAs above the corresponding cut-off values (including the cut-off values) are identified as putatively translated lncRNAs according to this coding filter. (See Additional file 12 for the other datasets)

The above three different coding metrics were calculated after removing footprints corresponding to contaminants. To filter footprints from among potential nonribosomal RNA–protein complexes, we first compared Ribo-seq reads from lncRNAs to those from mRNAs and found that reads of a specific length were enriched among lncRNAs (see Additional file 11). By identifying the sequences that were most frequently observed in these enriched reads from the full transcripts, we found that Ribo-seq reads may also be obtained from snRNAs, snoRNAs, and miRNAs. This finding is consistent with previous observations [35]. To integrate these three coding metrics to more stringently assess the ability of each ribosome-associated lncRNA to encode a peptide, we first generated cut-offs from mRNAs based on these three metrics and then applied these cut-offs to filter lncRNAs. Figure 4b and Additional file 12 show the distribution of FLOSS, RRS, and Framescore values among mRNAs as well as ribosome-associated and ribosome-free lncRNAs. Based on these three coding metrics, mRNAs consistently have the strongest coding abilities. Conversely, both the ribosome-associated and ribosome-free lncRNAs showed weak coding potential. Note that there is still a tendency toward higher coding scores for ribosome-associated lncRNAs relative to ribosome-free lncRNAs across all datasets, suggesting that some of the ribosome-associated lncRNAs may even encode peptides. Figure 4a (see Additional file 12 for other datasets) indicates how many of the putative ORFs in ribosome-associated lncRNAs pass the cut-offs for those three coding scores (FLOSS, RRS, and Framescore). In HeLa cells, for example, we observed 275 putative ORFs that passed those three coding filters, implying that translation of these putative ORFs may occur.

For convenience, we label the translation of a lncRNA containing an ORF that passed 0–3 coding filters as“A0”–“A3”. When there are multiple ORFs in a lncRNA, we chose the one with the highest number of coding filters it passed. Finally, we obtained a preliminary classification of lncRNAs in each dataset. Figure 3c shows that 5215 lncRNAs are expressed in HeLa cells, of which 3238 are classified as ribosome-free, while the rest are classified as ribosome-associated. Furthermore, among the ribosome-associated lncRNAs, 978 were classified as “A0,” which means we have no evidence of translation events on these lncRNAs, while 165 were classified as “A3,” which indicates that at least one putative ORF has passed all three coding filters (FLOSS, RRS, and Framescore) and means that credible translation of them may be happening.

### Identification of trans-lncRNAs, ribo-lncRNAs and noribo-lncRNAs across multiple datasets

**Fig. 5:**
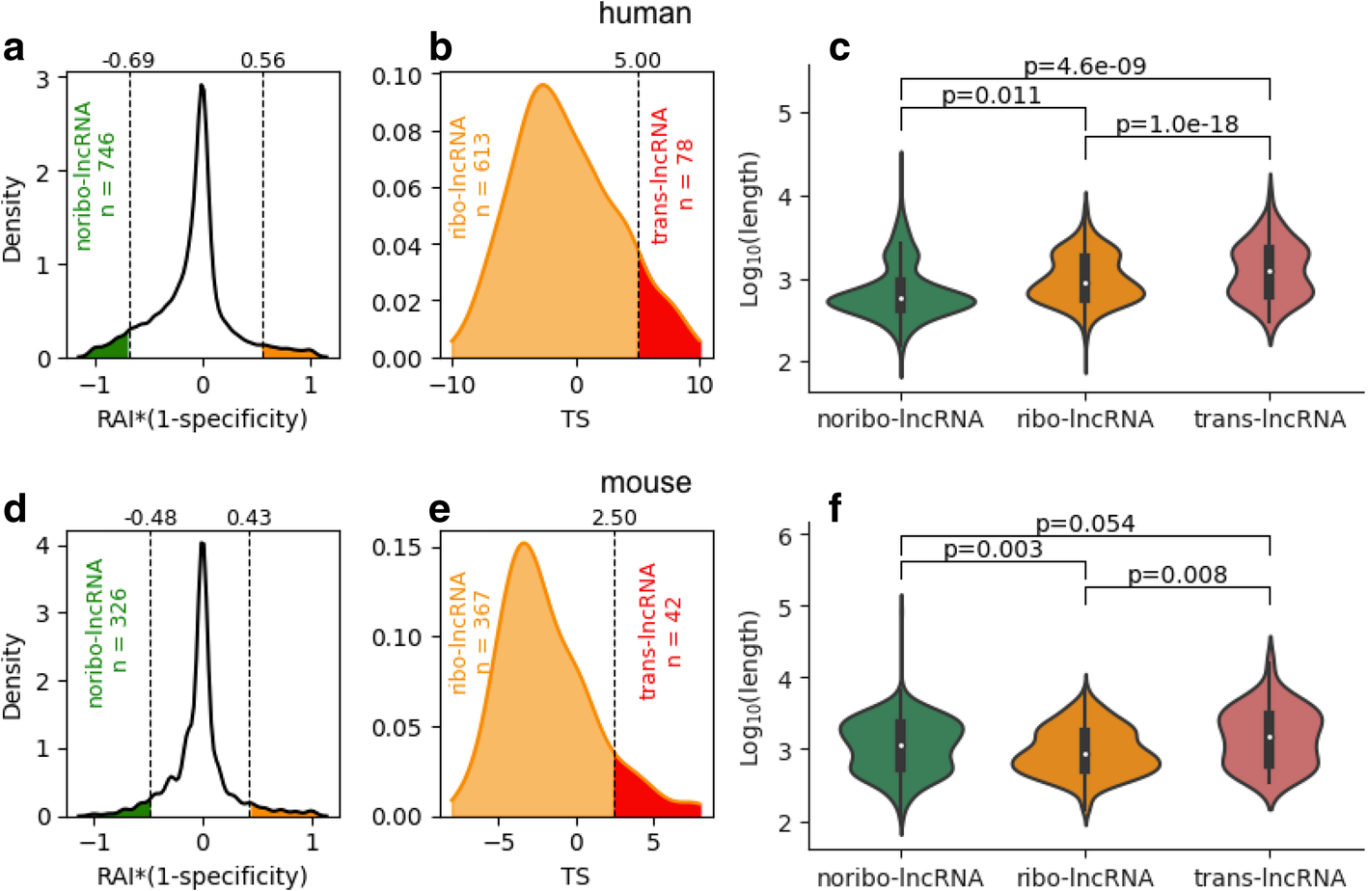
Classification of trans-lncRNAs, ribo-lncRNAs, and noribo-lncRNAs. **a** The kernel density of the RAI * (1 - spec) scores for human lncRNAs. Two vertical dashed lines represent the 5th percentile (left, upper bound for the reliable ribosome-free lncRNAs, termed “noribo-lncRNAs (green)”) and the 95th percentile (right, lower bound for the reliable ribosome-associated lncRNAs (orange) for further classification) of the RAI * (1 - spec) scores. **b** The kernel density of the TS scores for human ribosome-associated lncRNAs identified in **a**. Top 5% of lncRNAs were classified as “trans-lncRNAs (red)” suggesting that stable translation events are likely to occur among them. The remaining lncRNAs were finally classified as “ribo-lncRNA (orange)” indicating that there is an interaction with ribosomes in this part of lncRNAs, but no strong translation activity was observed. **c** Comparisons among trans-lncRNAs, ribo-lncRNAs, and noribo-lncRNAs for their transcript lengths in human. **d**–**f** show the results for mouse; *p*-values in **c** and **f** were calculated using two-sample *t*-tests

As a measure of the reliability of ribosomal associations for a particular lncRNA, we developed RAI * (1 - *spec*) to assess the integrated confidence of specific ribosomal associations across multiple pieces of experimental evidence. Here, RAI is a metric that measures ribosomal association across datasets in which the target lncRNA was expressed; *spec* is a metric for transcript expression specificity. We used a binary value to represent the ribosome density for a lncRNA in each experiment, as the ribosome density is normalized to transcript abundance in each dataset, which complicates the use of ribosome density across different datasets directly. A lncRNA with an RAI * (1 - *spec*) value of 1 indicates that the transcript consistently interacts with ribosomes among multiple datasets, and an RAI * (1 - *spec*) value of -1 denotes that this transcript is highly dissociated from ribosomes. (See “Methods” for the detailed definition of RAI * (1 - *spec*).) Additional file 8 lists the RAI * (1 - *spec*) values of all lncRNAs in the human and mouse datasets, respectively. As shown in Fig. 5a and 5d, we also used two threshold values—a low threshold at the 5th percentile and a high threshold at the 95th percentile—to determine high confident ribosome-free lncRNAs (termed “noribo-lncRNAs”) and ribosome-associated lncRNAs (termed “ribo-lncRNAs”). A lncRNA was classified as a noribo-lncRNA when its RAI * (1 - *spec*) value fell below the lower threshold and as a ribo-lncRNA when its RAI * (1 - *spec*) value exceeded the upper threshold. It is worth noting that the terms “ribosome-associated lncRNAs” and “ribosome-free lncRNAs” mentioned above are particularly used to categorize lncRNAs in a single dataset, whereas the terms “ribo-lncRNAs” and “noribo-lncRNAs” are defined across multiple datasets.

Furthermore, for ribo-lncRNAs that were widely expressed and commonly associated with ribosomes across multiple tissues or cell types, we determined if there are lncRNAs that can be translated. We presented the translation score (TS), a weighted sum function of translation events (A0 ∼A3), to evaluate the coding capacity for each ribo-lncRNA. The TS value for a lncRNA is expected to be positively related to the likelihood of this lncRNA contains an ORF encoding a peptide. We separated ribo-lncRNAs within the top 5% of TS values as putatively translated lncRNAs (termed “trans-lncRNAs”). (See Figs. 5b and 5e.) Overall, 746 noribo-lncRNAs, 613 ribo-lncRNAs, and 78 trans-lncRNAs in human (326 noribo-lncRNAs, 367 ribo-lncRNAs, and 42 trans-lncRNAs in mouse) were identified in this study (see Additional file 8 for the complete list of trans-lncRNAs, ribo-lncRNAs, and noribo-lncRNAs).

Footprint alignments, which were used to distinguish between ribosome-associated and ribosome-free lncRNAs, are more likely to occur on a longer transcript sequence. Thus, the first step is to evaluate the effect of transcript length on the RAI * (1 - *spec*) metric. We compared the transcript length among the trans-lncRNAs, ribo-lncRNAs, and noribo-lncRNAs (see Fig. 5c and 5f). Although we observed that the ribo-lncRNAs tended to be longer than noribo-lncRNAs in the human datasets (*p*<0.05), we also found the opposite result in the mouse datasets (*p*<0.01), which suggests that transcript length may not the dominant factor affecting the ribosomal association of lncRNAs. We also observed that, on average, the trans-lncRNAs were the longest in both human and mouse, suggesting transcript length is one of the important features that determines whether a transcript can encode a peptide.

### Exploring the biological characteristics of ribosome-associated lncRNAs

Next, we investigated the biological characteristics of ribosome-associated lncRNAs to determine their coding potential, sensitivity to nonsense-mediated decay, and cellular localization.

#### Coding potential

**Fig. 6:**
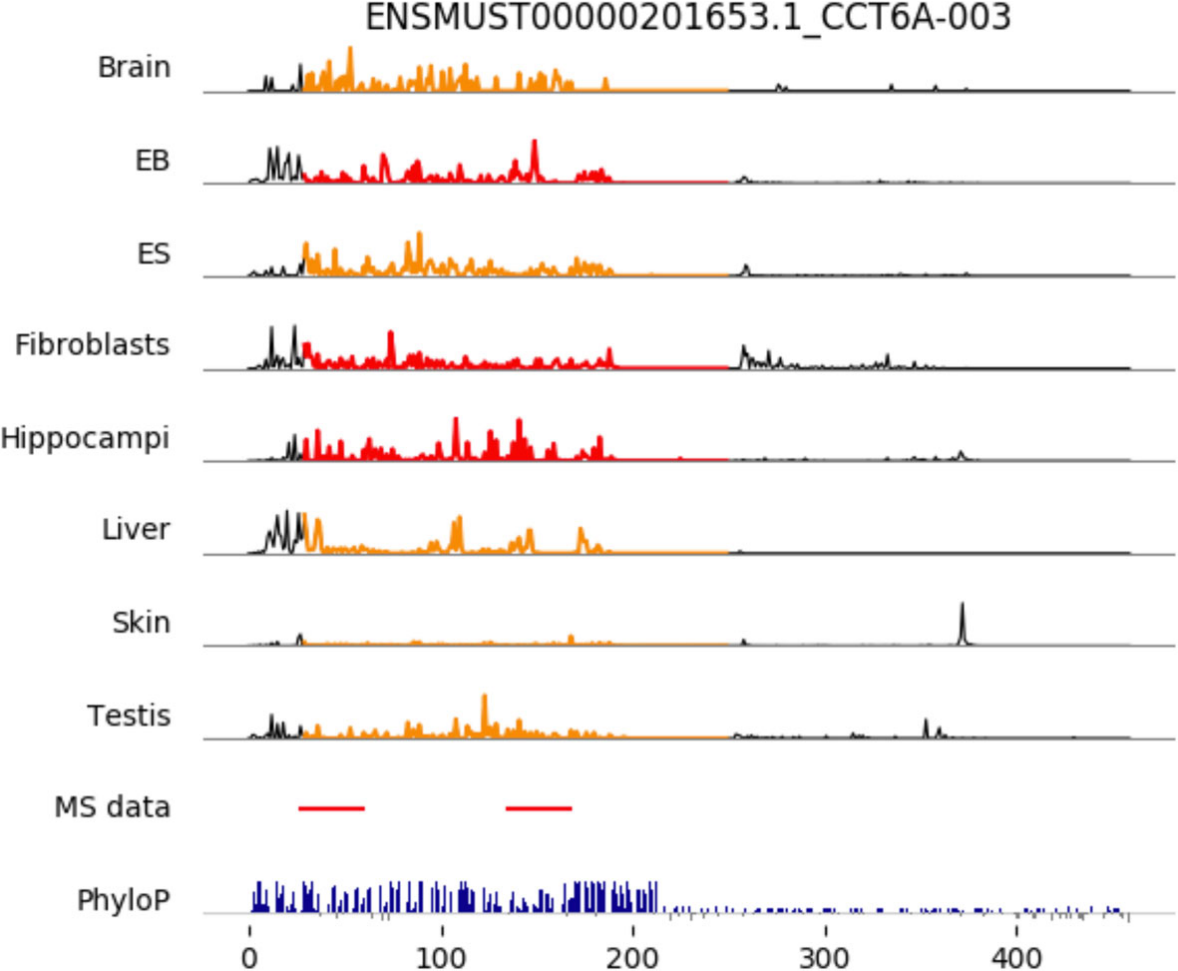
Overlapping of ribosome footprint coverage, mass spectrometry data, and sequence conservation (phyloP score) across mouse lncRNA CCT6A. Top eight panels indicate the ribosome coverage (arbitrary unit) across the CCT6A-003 transcript, where the colored region represents a putatively translated ORF identified by applying three coding metrics (FLOSS, RRS, and Framescore). Orange and red regions indicate this putative ORF has passed two and three coding filters, respectively. The MS data panel shows the overlapping of peptides transformed from mass spectrometry data in this transcript. The phyloP panel shows the base-wise conservation scores with positive values (blue) meaning slower evolution than expected, and negative values (gray) suggesting faster evolution than expected

To investigate whether the trans-lncRNAs detected in this study are consistent with mass spectrometry data, we aligned peptide sequences that were transformed from mass spectrometry data to lncRNAs. As expected, the lncRNAs with mappable peptides were significantly enriched among the trans-lncRNAs and ribo-lncRNAs for human and mouse (all *p*<0.001, see Table 2). In particular, the trans-lncRNAs were associated with the highest odds ratios (8.28 and 23.03 for human and mouse, respectively), which indicates that trans-lncRNAs have the highest potential for coding peptides. Figure 6 shows the footprint coverage, peptide alignment, and sequence conversation (phyloP score) for trans-lncRNA ENSMUST00000201653.1_CCT6A-003. For the footprint coverage, a colored region indicates the putative ORF predicted in this lncRNA. The peptide sequences transformed from the mass spectrometry data are consistently mapped onto this putative ORF. Also, we observed positive phyloP scores for the putative ORF, which indicates that this putative ORF sequence is evolutionarily conserved. Both metrics supported the hypothesis that the trans-lncRNA can encode peptides (see Additional files 13, 14, 15 and 16 for the details of other putative ORFs).

**Table 2:** Long non-coding RNAs supported by mass spectrometry data

	Human		Mouse	
	Total	MS supported (odds ratio)	Total	MS supported (odds ratio)
Trans-lncRNA	78	***5 (8.28)	42	***7 (23.03)
Ribo-lncRNA	613	***18 (4.16)	367	***10 (3.82)
Noribo-lncRNA	746	2 (0.32)	326	2 (0.73)
Other	12209	85 (0.40)	5525	33 (0.23)
Total	13646	110	6260	52

(One-sided Fisher’s exact test) *** *p*< 0.001

#### Cellular localization

**Fig. 7:**
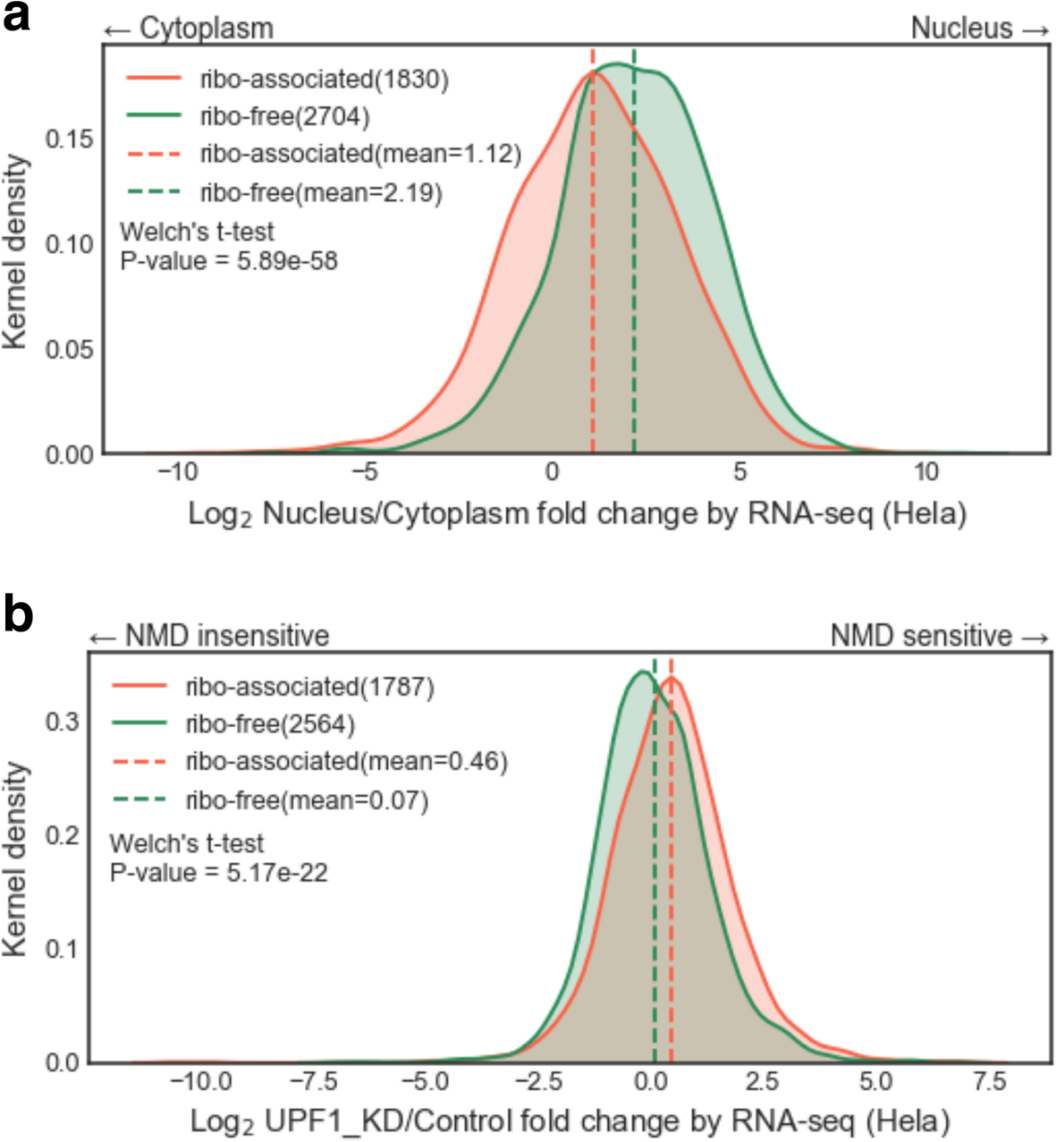
Comparisons between ribosome-associated lncRNAs and ribosome-free lncRNAs in HeLa cells (**a**) Cellular localization analysis. The fold changes of expression values were calculated between the nuclear and the cytoplasmic compartments to quantify the localization. (See Additional file 18 for the raw data used to generate this kernel density plot.) (**b**) Nonsense mediated decay (NMD) analysis. As UPF1 is an important NMD factor, we can use the fold changes of expression values between samples from a UPF1 knockdown and control to express NMD sensitivity. (See Additional file 19 for the raw data used to generate this kernel density plot.) The corresponding mean values are shown by vertical dashed lines; *p*-values were calculated using Welch’s *t*-test

We sought to examine whether the ribosome-associated lncRNAs are enriched in the cytoplasm where the ribosomes are located. Here, we used expression fold change, which compared the abundance of lncRNAs from the nuclear or the cytoplasmic fraction, to quantify the subcellular localization in HeLa cells (see “Methods” for details for generating the fold changes). Figure 7a indicates the kernel density of expression fold changes from the cytoplasmic fractions to the nuclear fractions for either ribosome-associated and ribosome-free lncRNAs. As expected, both the ribosome-associated lncRNAs and the ribosome-free lncRNAs were more likely to exist in the nucleus (*mean*=2.19 and 1.12 for ribosome-free lncRNAs and ribosome-associated lncRNAs, respectively). However, if compared with the ribosome-free lncRNAs, the ribosome-associated lncRNAs have a significant tendency to be present in the cytoplasm (*p*<0.001).

#### Sensitivity to nonsense-mediated decay

To test whether the ribo-lncRNAs are associated with nonsense-mediated decay (NMD), we investigated the differences in expression levels of various RNA populations in the presence (control) or absence (UPF1_KD) of NMD (see “Methods” for details to generate the fold change values). In HeLa cells, Fig. 7b is the kernel density of expression fold changes from the control samples to UPF1 knockdown samples for either ribosome-associated or ribosome-free lncRNAs. In our observations, the expression level of ribosome-free lncRNAs was not affected by NMD (*mean*=0.07). Interestingly, we found the expression level of ribosome-associated lncRNAs were significantly sensitive to NMD compared to ribosome-free lncRNAs (*mean*=0.46, *p*<0.001).

## Discussion

We emphasize that the term ribosomal association in this study refers to the ribosome translating or binding of a transcript, as ribosomes not only translate proteins but may also carry out other unclear functions by interacting with transcripts. To our knowledge, this is the first comprehensive study of ribosome–lncRNA interactions across multiple ribosome-profiling experiments in mammals, and it has several differences from previous studies: (i) more lncRNAs, including lincRNAs (long intergenic RNAs), were examined; (ii) a main focus on human and mouse because of the well-annotated lncRNAs for these two species; (iii) the use of the ribosome density metric and the cut-off value derived from 3 ^′^ UTRs to detect ribosomal associations of lncRNAs, which thus obtained robust detection rates of ribosome-associated lncRNAs over multiple independent datasets. We developed a novel tool, RAI * (1 - *spec*), to measure ribosomal association from multiple ribosome-profiling experiments. By using the RAI * (1 - *spec*) metric, we determined high-confidence ribosome-associated lncRNAs (ribo-lncRNAs) and ribosome-free lncRNAs (noribo-lncRNAs) and investigated the biological characteristics of ribosome-associated and ribosome-free lncRNAs involving coding potential, cellular localization, and NMD sensitivity.

Processed transcripts and retained introns were observed to prefer to associate with ribosomes, which suggests these two biotypes of lncRNAs are related to either protein-coding or ribosome-mediated regulation. For example, SEC22B has two transcript variants in the human genome, both of which were annotated as “processed transcript that does not contain an ORF” in GENCODE v25lift37(release 25 mapped to GRCh37). However, they had high RAI * (1 - *spec*) scores (both are 1, see Fig. 2 and Additional file 8), indicating their strong association with ribosomes. Moreover, we also observed a high translation score for SEC22B-001 (TS = 9.5), which indicates there is credible translation activity on this transcript. Strikingly, we found that SEC22B was removed from lncRNA category and annotated as a “protein coding” transcript in human genome h19 (GRCh38). Indeed, compared to ribosome-free lncRNAs, ribosome-associated lncRNAs have a higher protein-coding potential in the light of three variant coding metrics—FLOSS, RRS, and Framescore (see Fig. 4b and Additional file 12). This particular case of SEC22B suggests that some lncRNAs with high RAI * (1 - *spec*) and TS values could be protein/peptide coding transcripts. It seems plausible to use the RAI * (1 - *spec*) and TS in combination to examine the coding capacity of lncRNAs.

Most snoRNAs are located in introns of ribosomal protein genes and of genes encoding translation factors or nucleolar proteins. However, several noncoding genes are also reported as hosts for small nucleolar RNA (snoRNA) expression. Notably, as shown in Additional file 17, we observed snoRNA host gene-derived lncRNAs enriched in both trans-lncRNAs and ribo-lncRNAs, suggesting their interaction with ribosomes, which is consistent with previous studies [8, 36–38]. One possible reason for their association with ribosomes is that such lncRNAs are by-products of snoRNA production and are targeted to ribosomes, thus triggering the nonsense-mediated decay (NMD) pathway. Host gene-derived lncRNAs were reported to be sensitive to NMD [37], which provides indirect support of this hypothesis. In particular, GAS5 (growth arrest-specific 5) and ZFAS1 (ZNFX1 Antisense RNA 1), which were revealed by ribosomal association analysis in this study, have been reported to be associated with distinct biological functions. The GAS5 lncRNA sequence was determined to control transcriptional activity of apoptosis-related genes, while the NMD pathway appears to regulate the abundance of GAS5 transcripts [38]. The ZFAS1 lncRNA sequence was primarily identified to interact with the 40S ribosome subunit and reported to affect ribosomal protein modification [39]. The ZFAS1–ribosome interaction was also conserved in mouse (see Additional file 8), which suggests that the lncRNA may play a role in targeting the ribosome.

For lncRNAs, the dissociation of ribosomes illuminates lncRNA localization and functional studies. NEAT1 (nuclear enriched abundant transcript 1) is known to be a nuclear-enriched lncRNA. NEAT1 has been found to function as an important structural determinant of nuclear paraspeckles [40], which corresponds to the apparent ribosome-free NEAT1 (RAI *(1 - *spec*) = -0.8). TUG1 (taurine up-regulated gene 1) is a PRC2 (polycomb repressive complex 2)-associated lncRNA involved in cell-cycle regulation [41]. The longest transcript variant of TUG1 was highly ribosome-free (RAI * (1 - *spec*) = -1)). A TUG1 transcript variant of the human (ENST00000569149, RAI * (1 - *spec*) = 0.2)) and two transcript variants of the mouse (ENSMUST00000193809 and ENSMUST00000132077 with RAI * (1 - *spec*) = 1 and -1, respectively), on the contrary, displayed entirely different ribosomal association characteristics. This is also consistent with the finding that a unique peptide maps to TUG1 [42]. We, therefore, concluded that different transcript variants of lncRNAs act with different ribosome-associated properties, which may suggest a new functional class of lncRNAs regulated by alternative splicing coupled with ribosome targeting.

## Conclusions

In this study, we applied ribosome profiling data to identify interactions between lncRNAs and ribosomes. To our knowledge, this is the first report showing that a large fraction of lncRNAs–ribosome interactions over multiple independent studies are consistent and reliable in human and mouse. We developed the ribosomal association index (RAI) and used it with transcript expression specificity (spec) to measure the degree of reliability of lncRNA-ribosome interactions across multiple datasets. Furthermore, we used three different coding metrics (FLOSS, RRS, and Framescore) to assess the coding potential for ribosome-associated lncRNAs. LncRNAs detected to associate with ribosomes were observed to be more likely to be located in the cytoplasm and be more sensitive to NMD compared to ribosome-free lncRNAs. We also noticed that many ribosome-associated lncRNAs are tissue- or splicing-specific, which suggests these lncRNAs may target ribosomes under specific conditions to perform certain special functions. An interesting goal for future research is determining the biological mechanism underlying the condition-specific ribosomal association for lncRNAs. Future research may also identify the genomic characteristics of ribosome-associated lncRNAs and develop a method for distinguishing ribosome-associated lncRNAs from other RNA species. The complete list of ribosome associations of known lncRNAs in human and mouse are available online, from Additional file 8, which will be a useful resource for functional lncRNA studies.

## Additional files

Additional file 1 **Table S1.** Ribosome profiling datasets used in this study (human). (DOCX 287 kb)

Additional file 2 **Table S2.** Ribosome profiling datasets used in this study (mouse). (DOCX 288 kb)

Additional file 3 **Table S6.** Mapping statistics for RNA-seq and Ribo-seq reads. (XLSX 51.4 kb)

Additional file 4 **Figure S1.** Frequency distributions of Ribo-seq read lengths across CDSs, 5 ^′^/3 ^′^UTRs, and lncRNAs (human). (PDF 8253.44 kb)

Additional file 5 **Figure S2.** Frequency distributions of Ribo-seq read lengths across CDSs, 5 ^′^/3 ^′^UTRs, and lncRNAs (mouse). (PDF 10956.8 kb)

Additional file 6 **Table S3.** Genomic sequences, gene annotations, and contaminant sequences for human and mouse. (DOCX 279 kb)

Additional file 7 **Table S4.** Software and parameters used in this study. (DOCX 278 kb)

Additional file 8 **Table S8.** Ribosome association for human and mouse lncRNAs. (XLSX 1515.52 kb)

Additional file 9 **Figure S3.** The discrimination of ribosome-associated and ribosome-free lncRNAs by ribosome density in all selected datasets. (PDF 1146.88 kb)

Additional file 10 **Table S7.** Analysis of ribosomal associations of mRNAs and lncRNAs. (XLSX 46.7 kb)

Additional file 11 **Table S5.** Contaminant Ribo-seq reads derived from miRNAs, snRNAs, and snoRNAs are enriched in lncRNAs. (DOCX 280 kb)

Additional file 12 **Figure S4.** Analysis of coding potential by using FLOSS, RRS, and Framescore in all selected datasets. (PDF 1771.52 kb)

Additional file 13 **Table S9.** Alignment of mass spectrometry data to human trans-lncRNAs. (TSV 9.83 kb)

Additional file 14 **Table S10.** Alignment of mass spectrometry data to mouse trans-lncRNAs. (TSV 5.97 kb)

Additional file 15 **Table S14.** Putative ORFs in human lncRNAs. (TSV 7987.2 kb)

Additional file 16 **Table S15.** Putative ORFs in mouse lncRNAs. (TSV 5478.4 kb)

Additional file 17 **Table S13.** LncRNAs derived from snoRNA host genes are enriched in trans-lncRNAs and ribo-lncRNAs. (PDF 55.9 kb)

Additional file 18 **Table S11.** Fold change values for cellular localization analysis in HeLa cells. (XLSX 154 kb)

Additional file 19 **Table S12.** Fold change values for NMD analysis in HeLa cells. (XLSX 147 kb)

## Abbreviations

CDS: Coding sequence
FLOSS: Fragment length organization similarity score
lincRNA: Long intergenic noncoding RNA
lncRNA: Long noncoding RNA
miRNA: microRNA
NMD: Nonsense-mediated decay
ORF: Open reading frame
RAI: Ribosomal association index
Ribo-seq: Ribosome profiling
RNA-seq: RNA sequencing
RPKM: Reads per kilobase per total million mapped reads
RRS: Ribosome release score
snoRNA: Small nucleolar RNA
snRNA: Small nuclear RNA
*spec*: Transcript expression tissue-specificity
TEC: To be experimentally confirmed
TS: Translation score
UTR: Untranslated region
