# Supplementary material for: Identification and analysis of ribosome-associated lncRNAs using ribosome profiling data
Source: BMC Genomics. 2018 May 29;19:414. doi: 10.1186/s12864-018-4765-z (PMC5975437; doi:10.1186/s12864-018-4765-z)
Supplement: Supplementary file 1 — Table S1. Ribosome profiling datasets used in this study (human). (DOCX 287 kb) [file 12864_2018_4765_MOESM1_ESM.docx]

# Table S1. Ribosome profiling datasets used in this study (Human)

| **Source** | **Reference** | **Sample** | **RNA-seq** | **Ribo-seq** | **Description** |
| --- | --- | --- | --- | --- | --- |
| Brain | Gonzalez2014 [[1]](https://paperpile.com/c/39P52p/oaOfj) | normal-A | GSM1495249 | GSM1495244 | Normal brain |
|  |  | normal-B | GSM1495250 | GSM1495245 |  |
|  |  | normal-C | GSM1495251 | GSM1495246 |  |
|  |  | tumor-A | GSM1495252 | GSM1495247 |  |
|  |  | tumor-B | GSM1495253 | GSM1495248 |  |
| Breast | Rubio2014 [[2]](https://paperpile.com/c/39P52p/2ep44) | control-rep1 | GSM1503444 | GSM1503442 | Breast cancer (cell type: Ductal breast carcinoma; cell line: MDA-MB-231) |
|  |  | control-rep2 | GSM1503438 | GSM1503434 |  |
| Eye | Tanenbaum2015 [[3]](https://paperpile.com/c/39P52p/kx4sd) | G1-rep1 | GSM1657726 | GSM1657720 | Retinal pigment epithelial cells (cell type: RPE-1) |
|  |  | G1-rep2 | GSM1657727 | GSM1657721 |  |
|  |  | G2-rep1 | GSM1657728 | GSM1657722 |  |
|  |  | G2-rep2 | GSM1657729 | GSM1657723 |  |
|  |  | M-rep1 | GSM1657730 | GSM1657724 |  |
|  |  | M-rep2 | GSM1657731 | GSM1657725 |  |
| Fibroblasts | Shitrit2015 [[4]](https://paperpile.com/c/39P52p/gb27U) | control | GSM1712278 | GSM1712271 | Primary fibroblasts |
|  | Xu2016 [[5]](https://paperpile.com/c/39P52p/TyZRj) | wt-d-leucine | GSM1585204 | GSM1585210 | Fibroblast (supplemented with d-leucine or l-leucine) |
|  |  | wt-l-leucine | GSM1585205 | GSM1585211 |  |
| HEK | Eichhorn2014 [[6]](https://paperpile.com/c/39P52p/g0Fbe) | mock | GSM1479597 | GSM1479598 | HEK293T ( mock transfection) |
|  | Iwasaki2016 [[7]](https://paperpile.com/c/39P52p/J7kaP) | dmso-rep1 | GSM1720808 | GSM1720803 | HEK 293 T-REx cell (treatment: DMSO) |
|  |  | dmso-rep2 | GSM1720809 | GSM1720804 |  |
|  | Sidrauski2015 [[8]](https://paperpile.com/c/39P52p/vQk44) | control-a | GSM1606099 | GSM1606107 | HEK293T (treatment: untreated) |
|  |  | control-b | GSM1606100 | GSM1606108 |  |
|  | Subtelny2014 [[9]](https://paperpile.com/c/39P52p/4Qa8Z) | cyt | GSM1276541 | GSM1276542 | HEK293T (Cytoplasmically-enriched ) |
| HeLa | Guo2010 [[10]](https://paperpile.com/c/39P52p/AkGg0) | mock12hr | GSM546927 | GSM546926 | HeLa (transfection: mock) |
|  |  | mock32hr | GSM546921 | GSM546920 |  |
|  | Park2016 [[11]](https://paperpile.com/c/39P52p/J0MKF) | Mphase-rep1 | GSM2100590 | GSM2100598 | Hela (RNA-seq oligo-dT) |
|  |  | Mphase-rep2 | GSM2100591 | GSM2100599 |  |
|  |  | Sphase-rep1 | GSM2100587 | GSM2100596 |  |
|  |  | Sphase-rep2 | GSM2100588 | GSM2100597 |  |
|  | Zur2016 [[12]](https://paperpile.com/c/39P52p/Iz77m) | G1phase-exp1 | GSM1898014 GSM1898015 GSM1898016 | GSM1898018 GSM1898019 GSM1898020 | HeLa S3 cells |
|  |  | G1phase-exp2 | GSM1898017 | GSM1898021 |  |
|  |  | Mphase-exp1 | GSM1898006 GSM1898007 GSM1898008 | GSM1898010 GSM1898011 GSM1898012 |  |
|  |  | Mphase-exp2 | GSM1898009 | GSM1898013 |  |
| KOPT-K1 | Wolfe2014 [[13]](https://paperpile.com/c/39P52p/EMG0F) | dmso-rep1 | GSM1370699 | GSM1370695 | KOPT-K1 T-ALL cell line |
|  |  | dmso-rep2 | GSM1370700 | GSM1370696 |  |
| Lymphoblastoid | Cenik2015 [[14]](https://paperpile.com/c/39P52p/CD9TM) | GM12878-rep1 | GSM1609427 | GSM1609378 | EBV-transformed lymphoblastoid cells |
|  |  | GM12878-rep2 | GSM1609428 | GSM1609379 |  |
|  |  | GM12891-rep1 | GSM1609430 | GSM1609382 |  |
|  |  | GM12891-rep2 | GSM1609431 | GSM1609383 |  |
|  |  | GM12892-rep1 | GSM1609433 | GSM1609384 |  |
|  |  | GM12892-rep2 | GSM1609434 | GSM1609385 |  |
|  |  | GM19238-rep1 | GSM1609436 | GSM1609417 |  |
|  |  | GM19238-rep3 | GSM1609438 | GSM1609418 |  |
|  |  | GM19239-rep2 | GSM1609440 | GSM1609413 |  |
|  |  | GM19240-rep1 | GSM1609442 | GSM1609421 |  |
|  |  | GM19240-rep2 | GSM1609443 | GSM1609422 |  |
|  |  | GM19240-rep3 | GSM1609444 | GSM1609423 |  |
| Macrophages | Su2015 [[15]](https://paperpile.com/c/39P52p/XxKHd) | mock-rep1 | GSM1632596 | GSM1632594 | human primary macrophages (TLR2 stimulated; micrococccal nuclease) |
|  |  | mock-rep2 | GSM1632600 | GSM1632598 |  |
| Muscle | Wein2014 [[16]](https://paperpile.com/c/39P52p/ZiQD2) | control | GSM1356677 | GSM1356675 | Skeletal muscle (normal control) |
| Myeloma | Wiita2013 [[17]](https://paperpile.com/c/39P52p/WkNS9) | control | GSM1184591 | GSM1184592 | MM1.S myeloma cell line |
| PC3 | Hsieh2012 [[18]](https://paperpile.com/c/39P52p/nBi3S) | control-rep1 | GSM869036 | GSM869037 | PC3 (prostate cancer cells; sample type: polyA RNA; treatment: vehicle) |
|  |  | control-rep2 | GSM869042 | GSM869043 |  |
| U2OS | Eichhorn2014 [[6]](https://paperpile.com/c/39P52p/g0Fbe) | mock | GSM1479587 | GSM1479588 | U2OS cell line (mock-transfected, tRNA and rRNA depleted RNA-seq) |
|  | Guo2014 [[19]](https://paperpile.com/c/39P52p/ivXdV) | mock | GSM1248736 | GSM1248735 | U2OS cells (mock-transfected, poly(A)-selected RNA-seq) |
|  | Jang2015 [[20]](https://paperpile.com/c/39P52p/q9Vvc) | CT00-rep1 | GSM1371395 | GSM1371443 | U2OS (cell type: osteosarcoma) |
|  |  | CT00-rep2 | GSM1371407 | GSM1371455 |  |
|  |  | CT02-rep1 | GSM1371396 | GSM1371444 |  |
|  |  | CT02-rep2 | GSM1371408 | GSM1371456 |  |
|  |  | CT04-rep1 | GSM1371397 | GSM1371445 |  |
|  |  | CT04-rep2 | GSM1371409 | GSM1371457 |  |
|  |  | CT06-rep1 | GSM1371398 | GSM1371446 |  |
|  |  | CT06-rep2 | GSM1371410 | GSM1371458 |  |
|  |  | CT08-rep1 | GSM1371399 | GSM1371447 |  |
|  |  | CT08-rep2 | GSM1371411 | GSM1371459 |  |
|  |  | CT10-rep1 | GSM1371400 | GSM1371448 |  |
|  |  | CT10-rep2 | GSM1371412 | GSM1371460 |  |
|  |  | CT12-rep1 | GSM1371401 | GSM1371449 |  |
|  |  | CT12-rep2 | GSM1371413 | GSM1371461 |  |
|  |  | CT14-rep1 | GSM1371402 | GSM1371450 |  |
|  |  | CT14-rep2 | GSM1371414 | GSM1371462 |  |
|  |  | CT16-rep1 | GSM1371403 | GSM1371451 |  |
|  |  | CT16-rep2 | GSM1371415 | GSM1371463 |  |
|  |  | CT18-rep1 | GSM1371404 | GSM1371452 |  |
|  |  | CT18-rep2 | GSM1371416 | GSM1371464 |  |
|  |  | CT20-rep1 | GSM1371405 | GSM1371453 |  |
|  |  | CT20-rep2 | GSM1371417 | GSM1371465 |  |
|  |  | CT22-rep1 | GSM1371406 | GSM1371454 |  |
|  |  | CT22-rep2 | GSM1371418 | GSM1371466 |  |
| hES | Werner2015 [[21]](https://paperpile.com/c/39P52p/2GALZ) | control-rep1 | GSM1523640 | GSM1523624 | hES cell (cell line: H1) |
|  |  | control-rep2 | GSM1523648 | GSM1523632 |  |

#

# Reference

[1. Gonzalez C, Sims JS, Hornstein N, Mela A, Garcia F, Lei L, et al. Ribosome profiling reveals a cell-type-specific translational landscape in brain tumors. J. Neurosci. 2014;34:10924–36. Available from:](http://paperpile.com/b/39P52p/oaOfj) <http://dx.doi.org/10.1523/JNEUROSCI.0084-14.2014>

[2. Rubio CA, Weisburd B, Holderfield M, Arias C, Fang E, DeRisi JL, et al. Transcriptome-wide characterization of the eIF4A signature highlights plasticity in translation regulation. Genome Biol. 2014;15:476. Available from:](http://paperpile.com/b/39P52p/2ep44) <http://dx.doi.org/10.1186/s13059-014-0476-1>

[3. Tanenbaum ME, Stern-Ginossar N, Weissman JS, Vale RD. Regulation of mRNA translation during mitosis. Elife 2015;4. Available from:](http://paperpile.com/b/39P52p/kx4sd) <http://dx.doi.org/10.7554/eLife.07957>

[4. Tirosh O, Cohen Y, Shitrit A, Shani O, Le-Trilling VTK, Trilling M, et al. The Transcription and Translation Landscapes during Human Cytomegalovirus Infection Reveal Novel Host-Pathogen Interactions. PLoS Pathog. 2015;11:e1005288. Available from:](http://paperpile.com/b/39P52p/gb27U) <http://dx.doi.org/10.1371/journal.ppat.1005288>

[5. Xu B, Gogol M, Gaudenz K, Gerton JL. Improved transcription and translation with L-leucine stimulation of mTORC1 in Roberts syndrome. BMC Genomics 2016;17:25. Available from:](http://paperpile.com/b/39P52p/TyZRj) <http://dx.doi.org/10.1186/s12864-015-2354-y>

[6. Eichhorn SW, Guo H, McGeary SE, Rodriguez-Mias RA, Shin C, Baek D, et al. mRNA destabilization is the dominant effect of mammalian microRNAs by the time substantial repression ensues. Mol. Cell 2014;56:104–15. Available from:](http://paperpile.com/b/39P52p/g0Fbe) <http://dx.doi.org/10.1016/j.molcel.2014.08.028>

[7. Iwasaki S, Floor SN, Ingolia NT. Rocaglates convert DEAD-box protein eIF4A into a sequence-selective translational repressor. Nature 2016;534:558–61. Available from:](http://paperpile.com/b/39P52p/J7kaP) <http://dx.doi.org/10.1038/nature17978>

[8. Sidrauski C, McGeachy AM, Ingolia NT, Walter P. The small molecule ISRIB reverses the effects of eIF2α phosphorylation on translation and stress granule assembly. Elife 2015;4. Available from:](http://paperpile.com/b/39P52p/vQk44) <http://dx.doi.org/10.7554/eLife.05033>

[9. Subtelny AO, Eichhorn SW, Chen GR, Sive H, Bartel DP. Poly(A)-tail profiling reveals an embryonic switch in translational control. Nature 2014;508:66–71. Available from:](http://paperpile.com/b/39P52p/4Qa8Z) <http://dx.doi.org/10.1038/nature13007>

[10. Guo H, Ingolia NT, Weissman JS, Bartel DP. Mammalian microRNAs predominantly act to decrease target mRNA levels. Nature 2010;466:835–40. Available from:](http://paperpile.com/b/39P52p/AkGg0) <http://dx.doi.org/10.1038/nature09267>

[11. Park J-E, Yi H, Kim Y, Chang H, Kim VN. Regulation of Poly(A) Tail and Translation during the Somatic Cell Cycle. Mol. Cell 2016;62:462–71. Available from:](http://paperpile.com/b/39P52p/J0MKF) <http://dx.doi.org/10.1016/j.molcel.2016.04.007>

[12. Zur H, Aviner R, Tuller T. Complementary Post Transcriptional Regulatory Information is Detected by PUNCH-P and Ribosome Profiling. Sci. Rep. 2016;6:21635. Available from:](http://paperpile.com/b/39P52p/Iz77m) <http://dx.doi.org/10.1038/srep21635>

[13. Wolfe AL, Singh K, Zhong Y, Drewe P, Rajasekhar VK, Sanghvi VR, et al. RNA G-quadruplexes cause eIF4A-dependent oncogene translation in cancer. Nature 2014;513:65–70. Available from:](http://paperpile.com/b/39P52p/EMG0F) <http://dx.doi.org/10.1038/nature13485>

[14. Cenik C, Cenik ES, Byeon GW, Grubert F, Candille SI, Spacek D, et al. Integrative analysis of RNA, translation, and protein levels reveals distinct regulatory variation across humans. Genome Res. 2015;25:1610–21. Available from:](http://paperpile.com/b/39P52p/CD9TM) <http://dx.doi.org/10.1101/gr.193342.115>

[15. Su X, Yu Y, Zhong Y, Giannopoulou EG, Hu X, Liu H, et al. Interferon-γ regulates cellular metabolism and mRNA translation to potentiate macrophage activation. Nat. Immunol. 2015;16:838–49. Available from:](http://paperpile.com/b/39P52p/XxKHd) <http://dx.doi.org/10.1038/ni.3205>

[16. Wein N, Vulin A, Falzarano MS, Szigyarto CA-K, Maiti B, Findlay A, et al. Translation from a DMD exon 5 IRES results in a functional dystrophin isoform that attenuates dystrophinopathy in humans and mice. Nat. Med. 2014;20:992–1000. Available from:](http://paperpile.com/b/39P52p/ZiQD2) <http://dx.doi.org/10.1038/nm.3628>

[17. Wiita AP, Ziv E, Wiita PJ, Urisman A, Julien O, Burlingame AL, et al. Global cellular response to chemotherapy-induced apoptosis. Elife 2013;2:e01236. Available from:](http://paperpile.com/b/39P52p/WkNS9) <http://dx.doi.org/10.7554/eLife.01236>

[18. Hsieh AC, Liu Y, Edlind MP, Ingolia NT, Janes MR, Sher A, et al. The translational landscape of mTOR signalling steers cancer initiation and metastasis. Nature 2012;485:55–61. Available from:](http://paperpile.com/b/39P52p/nBi3S) <http://dx.doi.org/10.1038/nature10912>

[19. Guo JU, Agarwal V, Guo H, Bartel DP. Expanded identification and characterization of mammalian circular RNAs. Genome Biol. 2014;15:409. Available from:](http://paperpile.com/b/39P52p/ivXdV) <http://dx.doi.org/10.1186/s13059-014-0409-z>

[20. Jang C, Lahens NF, Hogenesch JB, Sehgal A. Ribosome profiling reveals an important role for translational control in circadian gene expression. Genome Res. 2015;25:1836–47. Available from:](http://paperpile.com/b/39P52p/q9Vvc) <http://dx.doi.org/10.1101/gr.191296.115>

[21. Werner A, Iwasaki S, McGourty CA, Medina-Ruiz S, Teerikorpi N, Fedrigo I, et al. Cell-fate determination by ubiquitin-dependent regulation of translation. Nature 2015;525:523–7. Available from:](http://paperpile.com/b/39P52p/2GALZ) <http://dx.doi.org/10.1038/nature14978>
