# Supplementary material for: Identification and analysis of ribosome-associated lncRNAs using ribosome profiling data
Source: BMC Genomics. 2018 May 29;19:414. doi: 10.1186/s12864-018-4765-z (PMC5975437; doi:10.1186/s12864-018-4765-z)
Supplement: Supplementary file 2 — Table S2. Ribosome profiling datasets used in this study (mouse). (DOCX 288 kb) [file 12864_2018_4765_MOESM2_ESM.docx]

# Table S2. Ribosome profiling datasets used in this study (Mouse)

| **Source** | **Reference** | **Sample** | **RNA-seq** | **Ribo-seq** | **Description** |
| --- | --- | --- | --- | --- | --- |
| Brain | Gonzalez2014 [[1]](https://paperpile.com/c/TLiL0m/EtzG1) | normal-A | GSM1245211 | GSM1245214 | tissue: PDGF/Cre tumor; Stage: end-stage |
|  |  | normal-B | GSM1245212 | GSM1245215 |  |
|  |  | normal-C | GSM1245213 | GSM1245216 |  |
|  |  | tumor-A | GSM1245217 | GSM1245223 |  |
|  |  | tumor-B | GSM1245218 | GSM1245224 |  |
|  |  | tumor-C | GSM1245219 | GSM1245225 |  |
| Fibroblast | Thoreen2012 [[2]](https://paperpile.com/c/TLiL0m/okorY) | wild-vehicle | GSM904895 | GSM904893 | Embryonic fibroblast (genotype: 4EBP1/2 +/+ p53 -/-; genetic: 129/Svj |
| Hippocampi | Cho2015 [[3]](https://paperpile.com/c/TLiL0m/P0nc6) | 10min-rep1 | GSM1853990 | GSM1853985 | Hippocampal tissue (strain: C57BL/6N) |
|  |  | 10min-rep2 | GSM1854000 | GSM1853995 |  |
|  |  | 10min-rep3 | GSM1854010 | GSM1854005 |  |
|  |  | 30min-rep1 | GSM1853991 | GSM1853986 |  |
|  |  | 30min-rep2 | GSM1854001 | GSM1853996 |  |
|  |  | 30min-rep3 | GSM1854011 | GSM1854006 |  |
|  |  | 4hr-rep1 | GSM1853992 | GSM1853987 |  |
|  |  | 4hr-rep2 | GSM1854002 | GSM1853997 |  |
|  |  | 4hr-rep3 | GSM1854012 | GSM1854007 |  |
|  |  | 5min-rep1 | GSM1853989 | GSM1853984 |  |
|  |  | 5min-rep2 | GSM1853999 | GSM1853994 |  |
|  |  | 5min-rep3 | GSM1854009 | GSM1854004 |  |
|  |  | control-rep1 | GSM1853988 | GSM1853983 |  |
|  |  | control-rep2 | GSM1853998 | GSM1853993 |  |
|  |  | control-rep3 | GSM1854008 | GSM1854003 |  |
| Liver | Alvarez2017 [[4]](https://paperpile.com/c/TLiL0m/zFial) | control-rep1 | GSM2219150 | GSM2219142 | Fetal liver (cell type: Erythroid progenitors; strain: C57BL/6; age: E14. |
|  |  | control-rep2 | GSM2219151 | GSM2219143 |  |
|  | Eichhorn2014 [[5]](https://paperpile.com/c/TLiL0m/wNlDT) | wt | GSM1479601 | GSM1479602 | Primary liver tissue (strain: C57BL/6; age: 6 weeks; sex: male) |
|  | Fradejas2017 [[6]](https://paperpile.com/c/TLiL0m/HNeJu) | secisbp2-wt-rep1 | GSM2227376 | GSM2227367 | Liver (age: 5-8 weeks; genotype: wild type) |
|  |  | secisbp2-wt-rep2 | GSM2227377 | GSM2227368 |  |
|  |  | trsp-wt-rep1 | GSM2227380 | GSM2227371 |  |
|  |  | trsp-wt-rep2 | GSM2227382 | GSM2227373 |  |
|  | Frederic2015 [[7]](https://paperpile.com/c/TLiL0m/znCWM) | ZT00-A | GSM1897722 | GSM1897856 | Liver (strain: C57BL/6; age: post natal day 12-16; genotype: wild type) |
|  |  | ZT00-B | GSM1897734 | GSM1897868 |  |
|  |  | ZT00-C | GSM1897751 | GSM1897880 |  |
|  |  | ZT00-D | GSM1897777 | GSM1897892 |  |
|  |  | ZT02-A | GSM1897723 | GSM1897857 |  |
|  |  | ZT02-B | GSM1897735 | GSM1897869 |  |
|  |  | ZT02-C | GSM1897754 | GSM1897881 |  |
|  |  | ZT02-D | GSM1897779 | GSM1897893 |  |
|  |  | ZT04-A | GSM1897724 | GSM1897858 |  |
|  |  | ZT04-B | GSM1897736 | GSM1897870 |  |
|  |  | ZT04-C | GSM1897756 | GSM1897882 |  |
|  |  | ZT04-D | GSM1897781 | GSM1897894 |  |
|  |  | ZT06-A | GSM1897725 | GSM1897859 |  |
|  |  | ZT06-B | GSM1897737 | GSM1897871 |  |
|  |  | ZT06-C | GSM1897758 | GSM1897883 |  |
|  |  | ZT06-D | GSM1897783 | GSM1897895 |  |
|  |  | ZT08-A | GSM1897726 | GSM1897860 |  |
|  |  | ZT08-B | GSM1897738 | GSM1897872 |  |
|  |  | ZT08-C | GSM1897760 | GSM1897884 |  |
|  |  | ZT08-D | GSM1897785 | GSM1897896 |  |
|  |  | ZT10-A | GSM1897727 | GSM1897861 |  |
|  |  | ZT10-B | GSM1897739 | GSM1897873 |  |
|  |  | ZT10-C | GSM1897762 | GSM1897885 |  |
|  |  | ZT10-D | GSM1897788 | GSM1897897 |  |
|  |  | ZT12-A | GSM1897728 | GSM1897862 |  |
|  |  | ZT12-B | GSM1897740 | GSM1897874 |  |
|  |  | ZT12-C | GSM1897764 | GSM1897886 |  |
|  |  | ZT12-D | GSM1897790 | GSM1897898 |  |
|  |  | ZT14-A | GSM1897729 | GSM1897863 |  |
|  |  | ZT14-B | GSM1897742 | GSM1897875 |  |
|  |  | ZT14-C | GSM1897766 | GSM1897887 |  |
|  |  | ZT14-D | GSM1897791 | GSM1897899 |  |
|  |  | ZT16-A | GSM1897730 | GSM1897864 |  |
|  |  | ZT16-B | GSM1897743 | GSM1897876 |  |
|  |  | ZT16-C | GSM1897768 | GSM1897888 |  |
|  |  | ZT16-D | GSM1897793 | GSM1897900 |  |
|  |  | ZT18-A | GSM1897731 | GSM1897865 |  |
|  |  | ZT18-B | GSM1897745 | GSM1897877 |  |
|  |  | ZT18-C | GSM1897771 | GSM1897889 |  |
|  |  | ZT18-D | GSM1897796 | GSM1897901 |  |
|  |  | ZT20-A | GSM1897732 | GSM1897866 |  |
|  |  | ZT20-B | GSM1897747 | GSM1897878 |  |
|  |  | ZT20-C | GSM1897773 | GSM1897890 |  |
|  |  | ZT20-D | GSM1897798 | GSM1897902 |  |
|  |  | ZT22-A | GSM1897733 | GSM1897867 |  |
|  |  | ZT22-B | GSM1897749 | GSM1897879 |  |
|  |  | ZT22-C | GSM1897775 | GSM1897891 |  |
|  |  | ZT22-D | GSM1897800 | GSM1897903 |  |
|  | Howard2013 [[8]](https://paperpile.com/c/TLiL0m/yJuoB) | wt | GSM1122211 | GSM1122205 | Liver (strain: FVB/N; age: 3 weeks; treatment: 6 week diet 0 ppm sele |
|  | Janich2015 [[9]](https://paperpile.com/c/TLiL0m/34TkM) | ZT0-rep1 | GSM1644100 | GSM1644076 | Liver (strain: C57BL/6JRj; age: 11-12 weeks; gender: male) |
|  |  | ZT0-rep2 | GSM1644101 | GSM1644077 |  |
|  |  | ZT10-rep1 | GSM1644110 | GSM1644086 |  |
|  |  | ZT10-rep2 | GSM1644111 | GSM1644087 |  |
|  |  | ZT12-rep1 | GSM1644112 | GSM1644088 |  |
|  |  | ZT12-rep2 | GSM1644113 | GSM1644089 |  |
|  |  | ZT14-rep1 | GSM1644114 | GSM1644090 |  |
|  |  | ZT14-rep2 | GSM1644115 | GSM1644091 |  |
|  |  | ZT16-rep1 | GSM1644116 | GSM1644092 |  |
|  |  | ZT16-rep2 | GSM1644117 | GSM1644093 |  |
|  |  | ZT18-rep1 | GSM1644118 | GSM1644094 |  |
|  |  | ZT18-rep2 | GSM1644119 | GSM1644095 |  |
|  |  | ZT2-rep1 | GSM1644102 | GSM1644078 |  |
|  |  | ZT2-rep2 | GSM1644103 | GSM1644079 |  |
|  |  | ZT20-rep1 | GSM1644120 | GSM1644096 |  |
|  |  | ZT20-rep2 | GSM1644121 | GSM1644097 |  |
|  |  | ZT22-rep1 | GSM1644122 | GSM1644098 |  |
|  |  | ZT22-rep2 | GSM1644123 | GSM1644099 |  |
|  |  | ZT4-rep1 | GSM1644104 | GSM1644080 |  |
|  |  | ZT4-rep2 | GSM1644105 | GSM1644081 |  |
|  |  | ZT6-rep1 | GSM1644106 | GSM1644082 |  |
|  |  | ZT6-rep2 | GSM1644107 | GSM1644083 |  |
|  |  | ZT8-rep1 | GSM1644108 | GSM1644084 |  |
|  |  | ZT8-rep2 | GSM1644109 | GSM1644085 |  |
| Skin | Blanco2016 [[10]](https://paperpile.com/c/TLiL0m/RSeNb) | wt1 | GSM1854037 | GSM1854031 | Skin (tumour stage: skin papilloma; strain: C57BL/6; age: 1 month) |
|  |  | wt2 | GSM1854038 | GSM1854032 |  |
|  |  | wt3 | GSM1854039 | GSM1854033 |  |
|  | Sendoel2017 [[11]](https://paperpile.com/c/TLiL0m/UeTgk) | wt-invivo-rep0 | GSM2199587 | GSM2199581 | Back skins (strain: R26-Sox2-IRES-eGFP fl/+; age: P4) |
|  |  | wt-invivo-rep1 | GSM2199588 | GSM2199582 |  |
| Testis | Castaneda2014 [[12]](https://paperpile.com/c/TLiL0m/02l3s) | wt-a | GSM1234250 | GSM1234248 | Testis (strain: 129SvJae; genotype: wild type) |
|  |  | wt-b | GSM1234254 | GSM1234252 |  |
| mEB | Ingolia2011 [[13]](https://paperpile.com/c/TLiL0m/7Og9F) | eb | GSM765286 | GSM765291 | Embryoid body (genotype: CReP+/-; GADD34+/+ (WT)) |
| mES | Hurt2013 [[14]](https://paperpile.com/c/TLiL0m/HmbN7) | control | GSM1024299 GSM1024300 | GSM1024311 | Embryonic stem cells (v6.5 cell line) |
|  | Ingolia2011 [[13]](https://paperpile.com/c/TLiL0m/7Og9F) | mes | GSM765288 | GSM765300 | ES cell (E14 cell line; genetic: 129/Ola) |
|  | Reid2014 [[15]](https://paperpile.com/c/TLiL0m/AYhDd) | cyt | GSM1299862 GSM1299863 | GSM1299858 GSM1299859 | Embryonic fibroblasts (genotype: CReP+/-; GADD34+/+ (WT)) |
|  |  | er | GSM1299860 GSM1299861 | GSM1299856 GSM1299857 |  |

**Reference**

[1. Gonzalez C, Sims JS, Hornstein N, Mela A, Garcia F, Lei L, et al. Ribosome profiling reveals a cell-type-specific translational landscape in brain tumors. J. Neurosci. 2014;34:10924–36. Available from:](http://paperpile.com/b/TLiL0m/EtzG1) <http://dx.doi.org/10.1523/JNEUROSCI.0084-14.2014>

[2. Thoreen CC, Chantranupong L, Keys HR, Wang T, Gray NS, Sabatini DM. A unifying model for mTORC1-mediated regulation of mRNA translation. Nature 2012;485:109–13. Available from:](http://paperpile.com/b/TLiL0m/okorY) <http://dx.doi.org/10.1038/nature11083>

[3. Cho J, Yu N-K, Choi J-H, Sim S-E, Kang SJ, Kwak C, et al. Multiple repressive mechanisms in the hippocampus during memory formation. Science 2015;350:82–7. Available from:](http://paperpile.com/b/TLiL0m/P0nc6) <http://dx.doi.org/10.1126/science.aac7368>

[4. Alvarez-Dominguez JR, Zhang X, Hu W. Widespread and dynamic translational control of red blood cell development. Blood 2017;129:619–29. Available from:](http://paperpile.com/b/TLiL0m/zFial) <http://dx.doi.org/10.1182/blood-2016-09-741835>

[5. Eichhorn SW, Guo H, McGeary SE, Rodriguez-Mias RA, Shin C, Baek D, et al. mRNA destabilization is the dominant effect of mammalian microRNAs by the time substantial repression ensues. Mol. Cell 2014;56:104–15. Available from:](http://paperpile.com/b/TLiL0m/wNlDT) <http://dx.doi.org/10.1016/j.molcel.2014.08.028>

[6. Fradejas-Villar N, Seeher S, Anderson CB, Doengi M, Carlson BA, Hatfield DL, et al. The RNA-binding protein Secisbp2 differentially modulates UGA codon reassignment and RNA decay. Nucleic Acids Res. 2017;45:4094–107. Available from:](http://paperpile.com/b/TLiL0m/HNeJu) <http://dx.doi.org/10.1093/nar/gkw1255>

[7. Atger F, Gobet C, Marquis J, Martin E, Wang J, Weger B, et al. Circadian and feeding rhythms differentially affect rhythmic mRNA transcription and translation in mouse liver. Proc. Natl. Acad. Sci. U. S. A. 2015;112:E6579–88. Available from:](http://paperpile.com/b/TLiL0m/znCWM) <http://dx.doi.org/10.1073/pnas.1515308112>

[8. Howard MT, Carlson BA, Anderson CB, Hatfield DL. Translational redefinition of UGA codons is regulated by selenium availability. J. Biol. Chem. 2013;288:19401–13. Available from:](http://paperpile.com/b/TLiL0m/yJuoB) <http://dx.doi.org/10.1074/jbc.M113.481051>

[9. Janich P, Arpat AB, Castelo-Szekely V, Lopes M, Gatfield D. Ribosome profiling reveals the rhythmic liver translatome and circadian clock regulation by upstream open reading frames. Genome Res. 2015;25:1848–59. Available from:](http://paperpile.com/b/TLiL0m/34TkM) <http://dx.doi.org/10.1101/gr.195404.115>

[10. Blanco S, Bandiera R, Popis M, Hussain S, Lombard P, Aleksic J, et al. Stem cell function and stress response are controlled by protein synthesis. Nature 2016;534:335–40. Available from:](http://paperpile.com/b/TLiL0m/RSeNb) <http://dx.doi.org/10.1038/nature18282>

[11. Sendoel A, Dunn JG, Rodriguez EH, Naik S, Gomez NC, Hurwitz B, et al. Translation from unconventional 5’ start sites drives tumour initiation. Nature 2017;541:494–9. Available from:](http://paperpile.com/b/TLiL0m/UeTgk) <http://dx.doi.org/10.1038/nature21036>

[12. Castañeda J, Genzor P, van der Heijden GW, Sarkeshik A, Yates JR 3rd, Ingolia NT, et al. Reduced pachytene piRNAs and translation underlie spermiogenic arrest in Maelstrom mutant mice. EMBO J. 2014;33:1999–2019. Available from:](http://paperpile.com/b/TLiL0m/02l3s) <http://dx.doi.org/10.15252/embj.201386855>

[13. Ingolia NT, Lareau LF, Weissman JS. Ribosome profiling of mouse embryonic stem cells reveals the complexity and dynamics of mammalian proteomes. Cell 2011;147:789–802. Available from:](http://paperpile.com/b/TLiL0m/7Og9F) <http://dx.doi.org/10.1016/j.cell.2011.10.002>

[14. Hurt JA, Robertson AD, Burge CB. Global analyses of UPF1 binding and function reveal expanded scope of nonsense-mediated mRNA decay. Genome Res. 2013;23:1636–50. Available from:](http://paperpile.com/b/TLiL0m/HmbN7) <http://dx.doi.org/10.1101/gr.157354.113>

[15. Reid DW, Chen Q, Tay AS-L, Shenolikar S, Nicchitta CV. The unfolded protein response triggers selective mRNA release from the endoplasmic reticulum. Cell 2014;158:1362–74. Available from:](http://paperpile.com/b/TLiL0m/AYhDd) <http://dx.doi.org/10.1016/j.cell.2014.08.012>
