# Supplementary material for: Identification and analysis of ribosome-associated lncRNAs using ribosome profiling data
Source: BMC Genomics. 2018 May 29;19:414. doi: 10.1186/s12864-018-4765-z (PMC5975437; doi:10.1186/s12864-018-4765-z)
Supplement: Supplementary file 4 — Figure S1. Frequency distributions of Ribo-seq read lengths across CDSs, 5 ′/3 ′UTRs, and lncRNAs (human). (PDF 8253.44 kb) [file 12864_2018_4765_MOESM4_ESM.pdf]

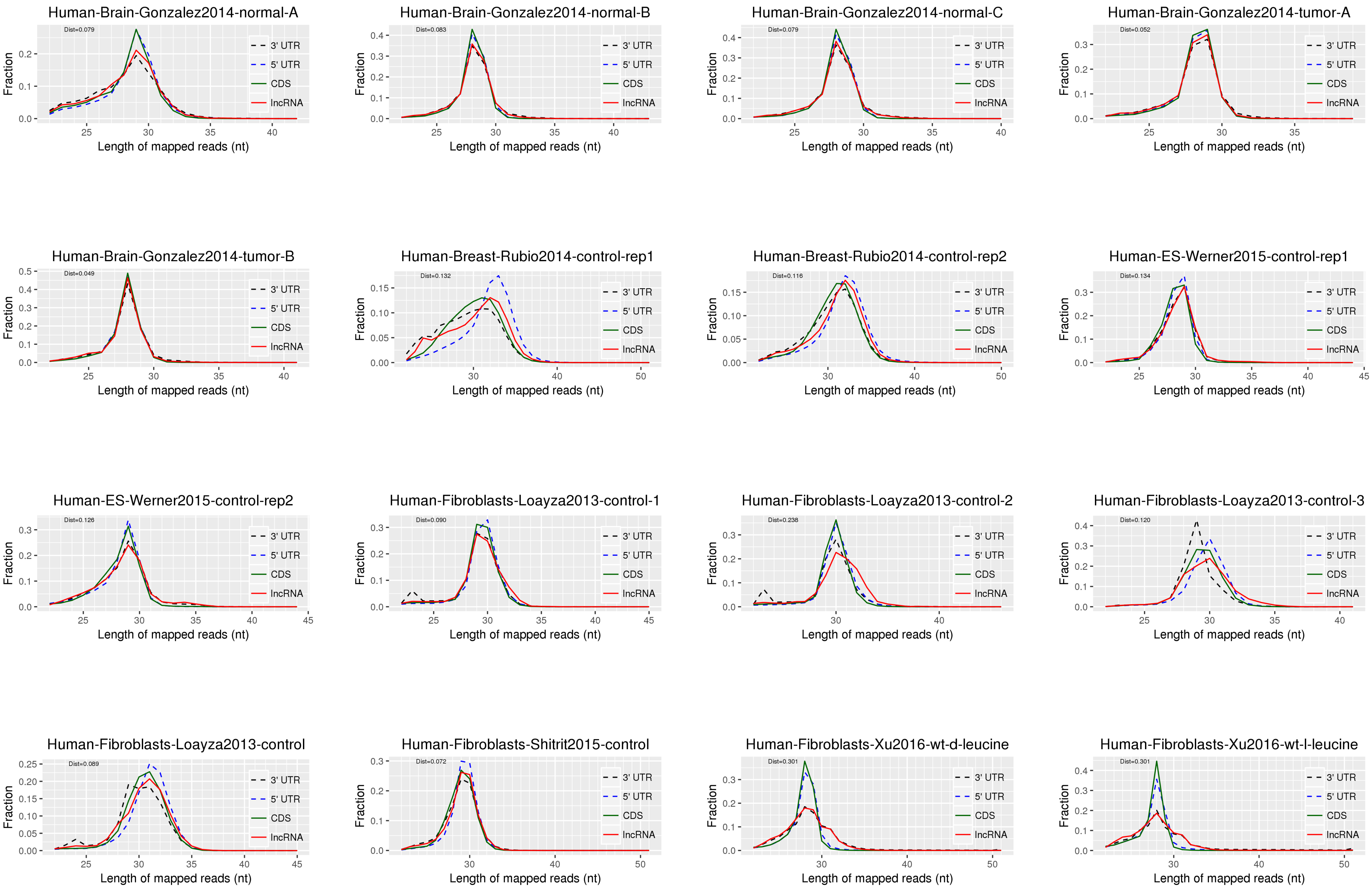
Human-Brain-Gonzalez2014-normal-A
Dist=0.079
Human-Brain-Gonzalez2014-normal-B
Dist=0.083
Human-Brain-Gonzalez2014-normal-C
Dist=0.079
Human-Brain-Gonzalez2014-tumor-A
Dist=0.052
Human-Brain-Gonzalez2014-tumor-B
Dist=0.049
Human-Breast-Rubio2014-control-rep1
Dist=0.132
Human-Breast-Rubio2014-control-rep2
Dist=0.116
Human-ES-Werner2015-control-rep1
Dist=0.134
Human-ES-Werner2015-control-rep2
Dist=0.126
Human-Fibroblasts-Loayza2013-control-1
Dist=0.090
Human-Fibroblasts-Loayza2013-control-2
Dist=0.238
Human-Fibroblasts-Loayza2013-control-3
Dist=0.120
Human-Fibroblasts-Loayza2013-control
Dist=0.089
Human-Fibroblasts-Shitrit2015-control
Dist=0.072
Human-Fibroblasts-Xu2016-wt-d-leucine
Dist=0.301
Human-Fibroblasts-Xu2016-wt-l-leucine
Dist=0.301
3' UTR
5' UTR
CDS
lncRNA
Fraction
Length of mapped reads (nt)

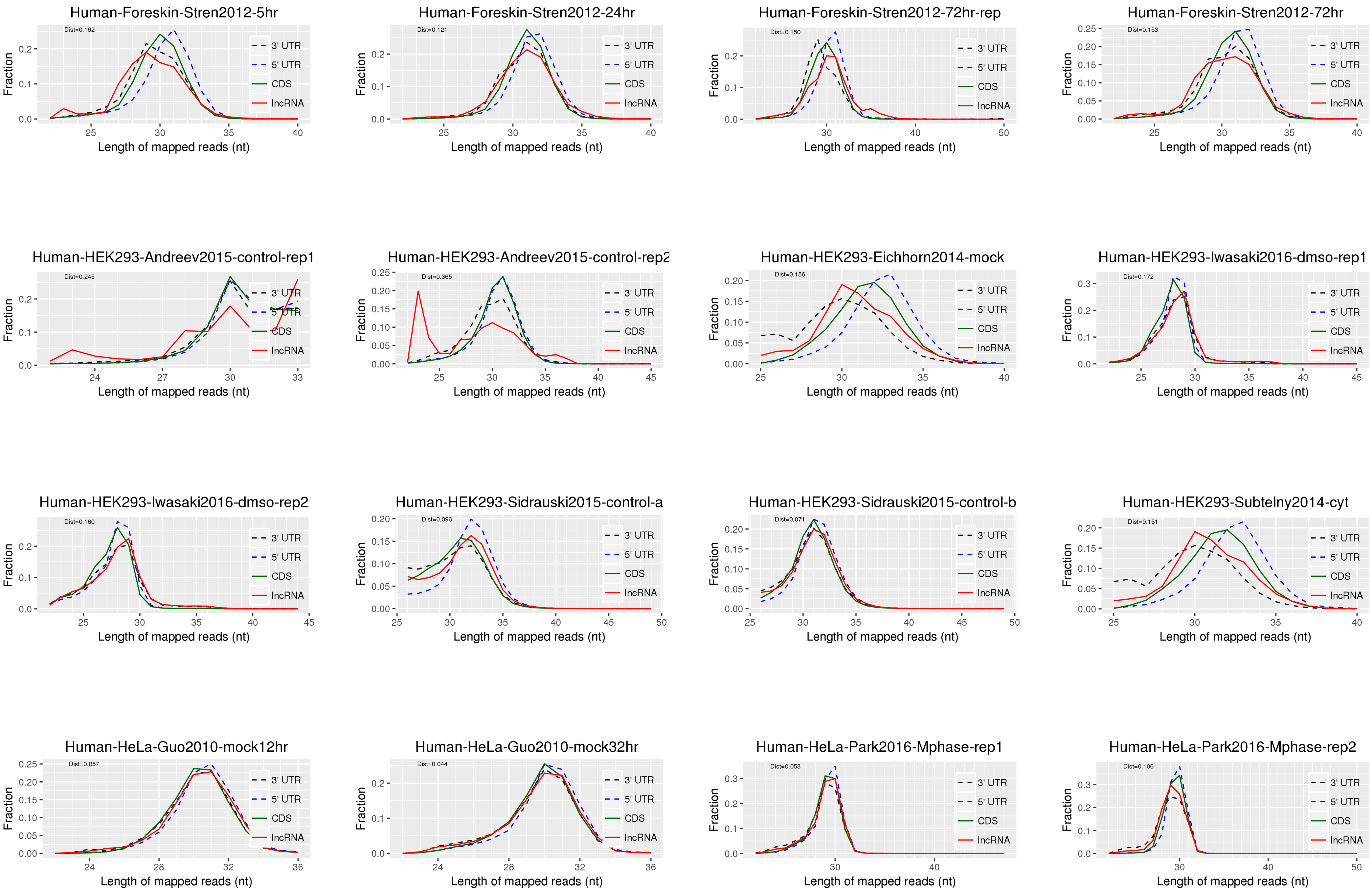

Human-Foreskin-Stren2012-5hr
Dist=0.162
Human-Foreskin-Stren2012-24hr
Dist=0.121
Human-Foreskin-Stren2012-72hr-rep
Dist=0.150
Human-Foreskin-Stren2012-72hr
Dist=0.153
Human-HEK293-Andreev2015-control-rep1
Dist=0.245
Human-HEK293-Andreev2015-control-rep2
Dist=0.365
Human-HEK293-Eichhorn2014-mock
Dist=0.156
Human-HEK293-Iwasaki2016-dmso-rep1
Dist=0.172
Human-HEK293-Iwasaki2016-dmso-rep2
Dist=0.160
Human-HEK293-Sidrauski2015-control-a
Dist=0.096
Human-HEK293-Sidrauski2015-control-b
Dist=0.071
Human-HEK293-Subtelny2014-cyt
Dist=0.151
Human-HeLa-Guo2010-mock12hr
Dist=0.057
Human-HeLa-Guo2010-mock32hr
Dist=0.044
Human-HeLa-Park2016-Mphase-rep1
Dist=0.053
Human-HeLa-Park2016-Mphase-rep2
Dist=0.106
3' UTR
5' UTR
CDS
lncRNA
Fraction
Length of mapped reads (nt)

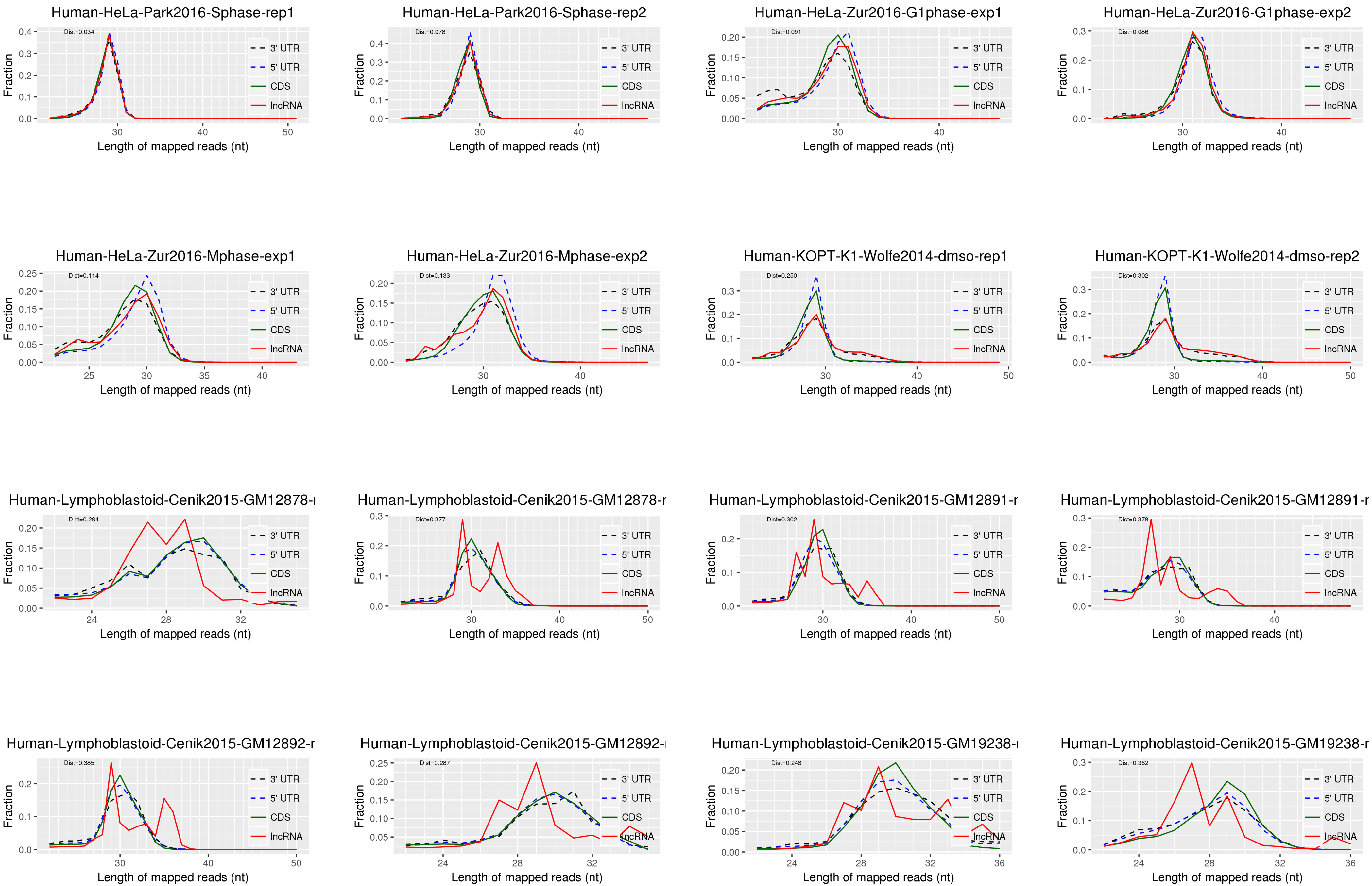
Human-HeLa-Park2016-Sphase-rep1
Dist=0.034
Human-HeLa-Park2016-Sphase-rep2
Dist=0.078
Human-HeLa-Zur2016-G1phase-exp1
Dist=0.091
Human-HeLa-Zur2016-G1phase-exp2
Dist=0.086
Human-HeLa-Zur2016-Mphase-exp1
Dist=0.114
Human-HeLa-Zur2016-Mphase-exp2
Dist=0.133
Human-KOPT-K1-Wolfe2014-dmso-rep1
Dist=0.250
Human-KOPT-K1-Wolfe2014-dmso-rep2
Dist=0.302
Human-Lymphoblastoid-Cenik2015-GM12878-
Dist=0.284
Human-Lymphoblastoid-Cenik2015-GM12878-r
Dist=0.377
Human-Lymphoblastoid-Cenik2015-GM12891-r
Dist=0.302
Human-Lymphoblastoid-Cenik2015-GM12891-r
Dist=0.378
Human-Lymphoblastoid-Cenik2015-GM12892-r
Dist=0.385
Human-Lymphoblastoid-Cenik2015-GM12892-
Dist=0.287
Human-Lymphoblastoid-Cenik2015-GM19238-
Dist=0.248
Human-Lymphoblastoid-Cenik2015-GM19238-r
Dist=0.362
3' UTR
5' UTR
CDS
lncRNA
Fraction
Length of mapped reads (nt)

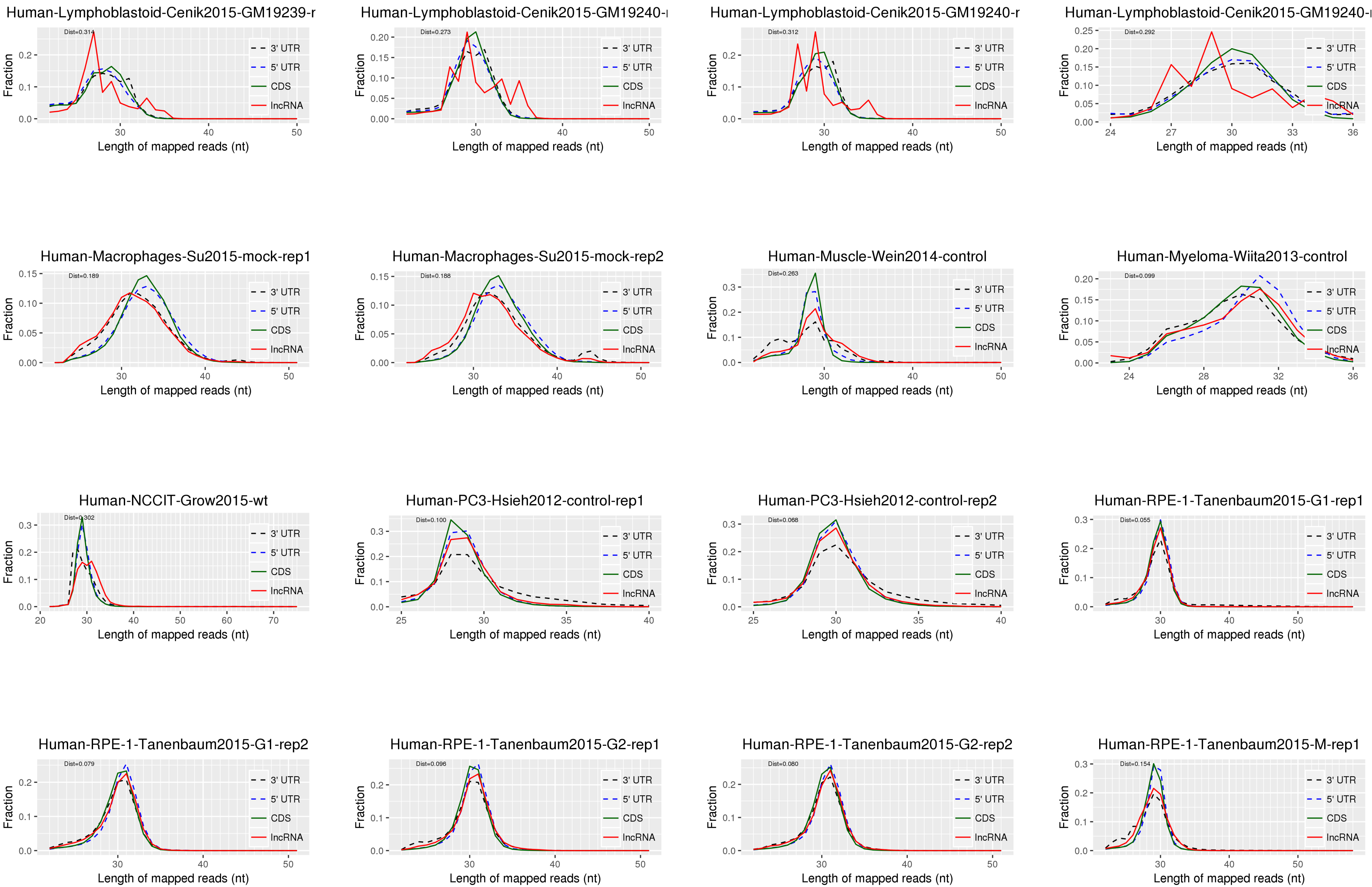

Human-Lymphoblastoid-Cenik2015-GM19239-r
Dist=0.314
Human-Lymphoblastoid-Cenik2015-GM19240-r
Dist=0.273
Human-Lymphoblastoid-Cenik2015-GM19240-r
Dist=0.312
Human-Lymphoblastoid-Cenik2015-GM19240-r
Dist=0.292
Human-Macrophages-Su2015-mock-rep1
Dist=0.189
Human-Macrophages-Su2015-mock-rep2
Dist=0.188
Human-Muscle-Wein2014-control
Dist=0.263
Human-Myeloma-Wiita2013-control
Dist=0.099
Human-NCCIT-Grow2015-wt
Dist=0.302
Human-PC3-Hsieh2012-control-rep1
Dist=0.100
Human-PC3-Hsieh2012-control-rep2
Dist=0.068
Human-RPE-1-Tanenbaum2015-G1-rep1
Dist=0.055
Human-RPE-1-Tanenbaum2015-G1-rep2
Dist=0.079
Human-RPE-1-Tanenbaum2015-G2-rep1
Dist=0.096
Human-RPE-1-Tanenbaum2015-G2-rep2
Dist=0.080
Human-RPE-1-Tanenbaum2015-M-rep1
Dist=0.154
3' UTR
5' UTR
CDS
lncRNA
Fraction
Length of mapped reads (nt)

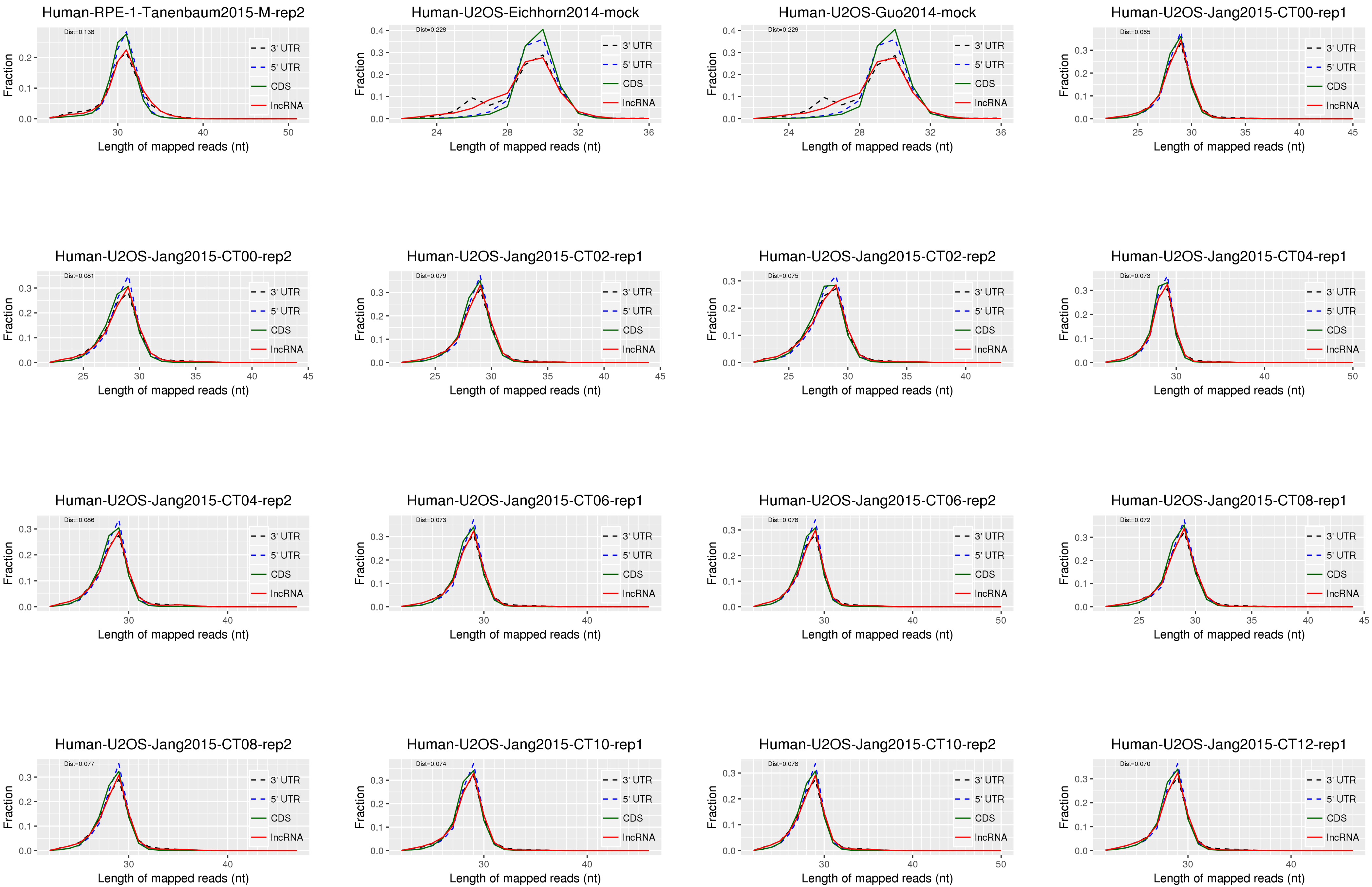
Human-RPE-1-Tanenbaum2015-M-rep2
Dist=0.138
Human-U2OS-Eichhorn2014-mock
Dist=0.228
Human-U2OS-Guo2014-mock
Dist=0.229
Human-U2OS-Jang2015-CT00-rep1
Dist=0.065
Human-U2OS-Jang2015-CT00-rep2
Dist=0.081
Human-U2OS-Jang2015-CT02-rep1
Dist=0.079
Human-U2OS-Jang2015-CT02-rep2
Dist=0.075
Human-U2OS-Jang2015-CT04-rep1
Dist=0.073
Human-U2OS-Jang2015-CT04-rep2
Dist=0.086
Human-U2OS-Jang2015-CT06-rep1
Dist=0.073
Human-U2OS-Jang2015-CT06-rep2
Dist=0.078
Human-U2OS-Jang2015-CT08-rep1
Dist=0.072
Human-U2OS-Jang2015-CT08-rep2
Dist=0.077
Human-U2OS-Jang2015-CT10-rep1
Dist=0.074
Human-U2OS-Jang2015-CT10-rep2
Dist=0.078
Human-U2OS-Jang2015-CT12-rep1
Dist=0.070
3' UTR
5' UTR
CDS
lncRNA
Fraction
Length of mapped reads (nt)

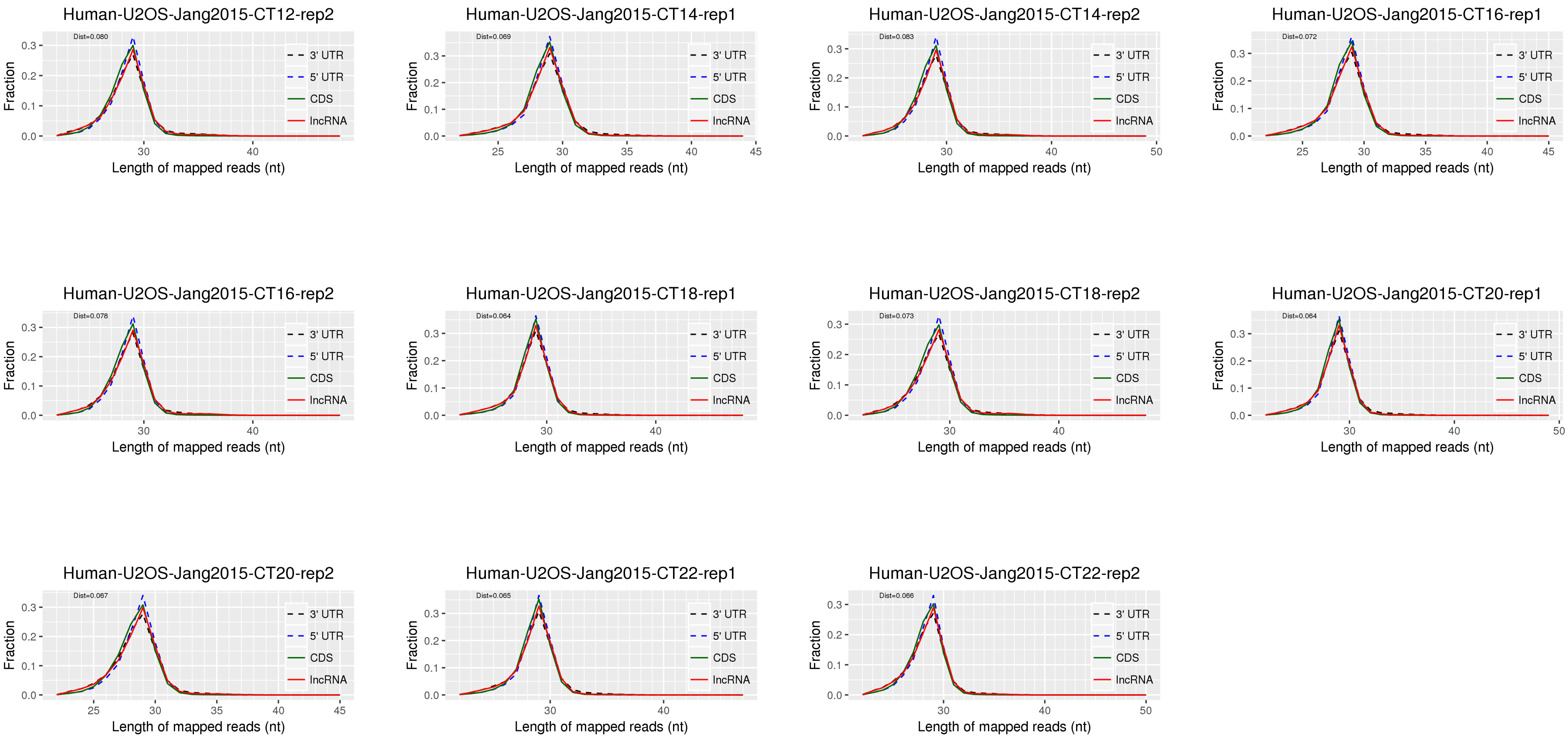
Human-U2OS-Jang2015-CT12-rep2
Dist=0.080
Human-U2OS-Jang2015-CT14-rep1
Dist=0.069
Human-U2OS-Jang2015-CT14-rep2
Dist=0.083
Human-U2OS-Jang2015-CT16-rep1
Dist=0.072
Human-U2OS-Jang2015-CT16-rep2
Dist=0.078
Human-U2OS-Jang2015-CT18-rep1
Dist=0.064
Human-U2OS-Jang2015-CT18-rep2
Dist=0.073
Human-U2OS-Jang2015-CT20-rep1
Dist=0.064
Human-U2OS-Jang2015-CT20-rep2
Dist=0.067
Human-U2OS-Jang2015-CT22-rep1
Dist=0.065
Human-U2OS-Jang2015-CT22-rep2
Dist=0.066
Fraction
Length of mapped reads (nt)
3' UTR
5' UTR
CDS
lncRNA
