# Supplementary figures and images for: Identification and analysis of ribosome-associated lncRNAs using ribosome profiling data
Source: BMC Genomics. 2018 May 29;19:414. doi: 10.1186/s12864-018-4765-z (PMC5975437; doi:10.1186/s12864-018-4765-z)

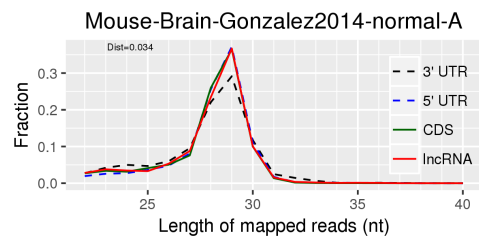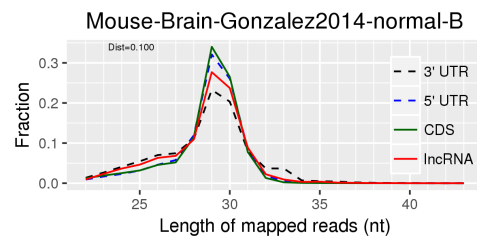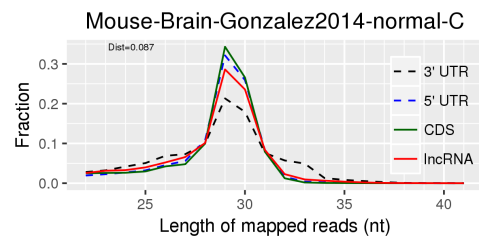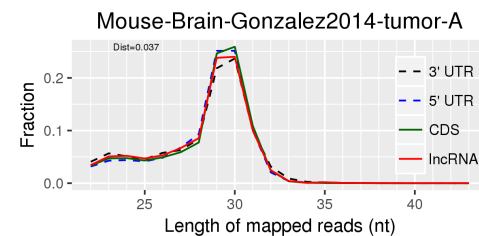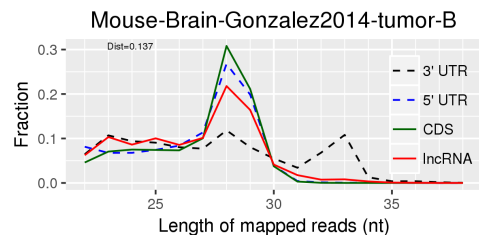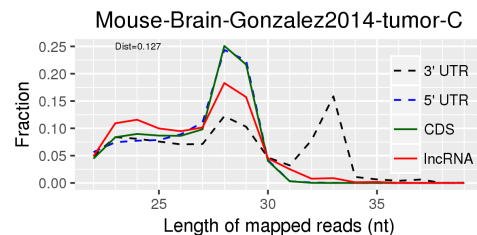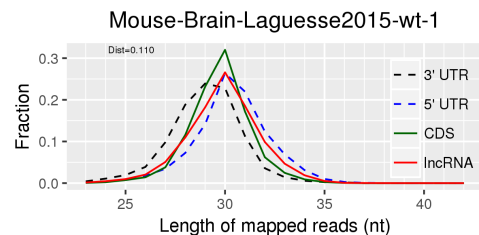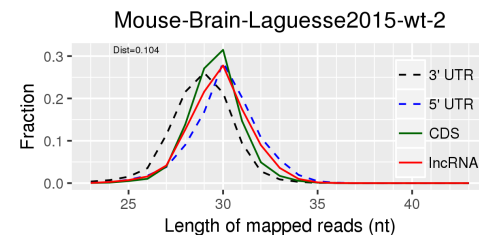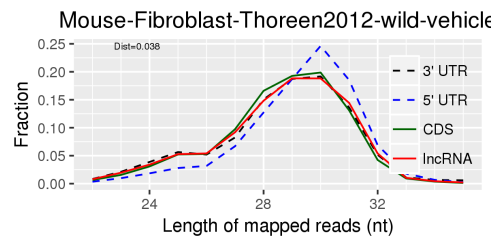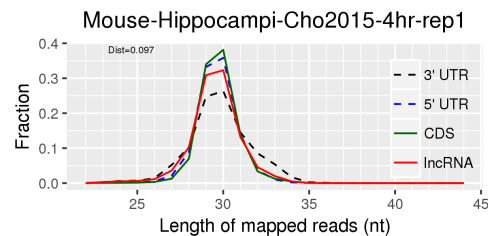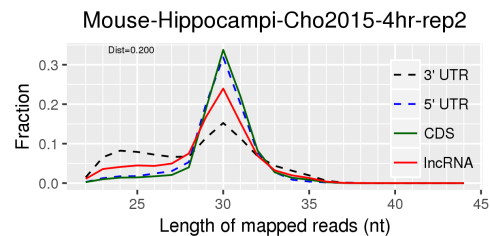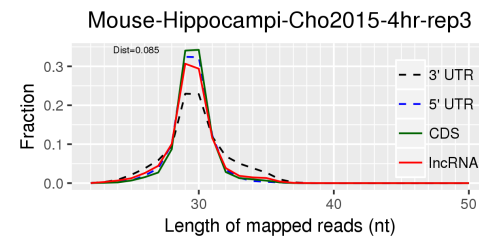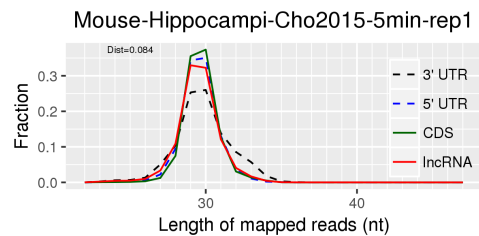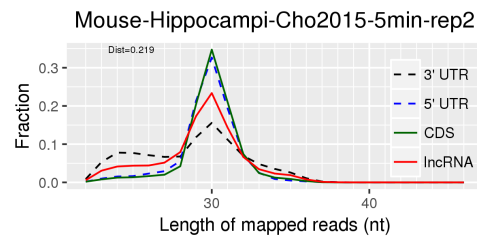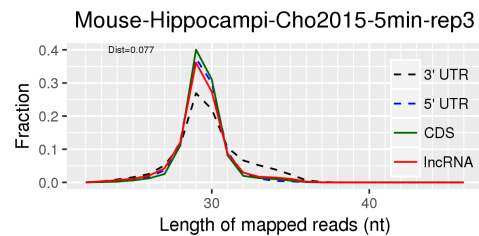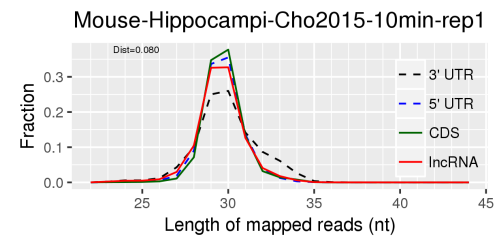

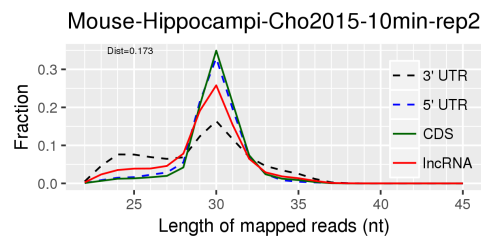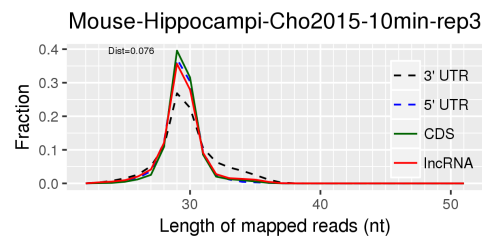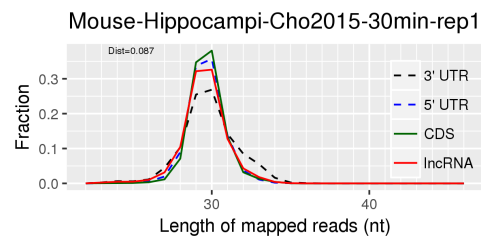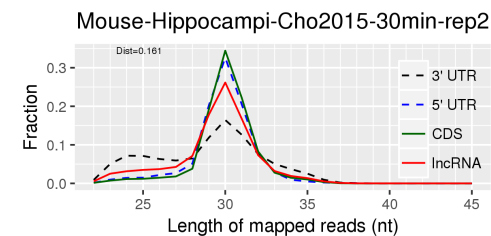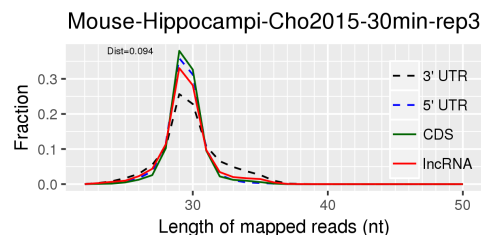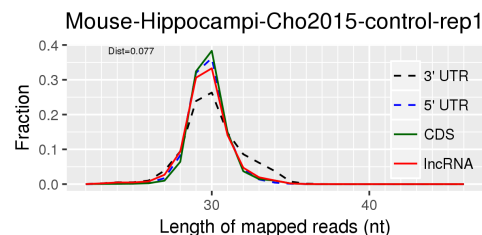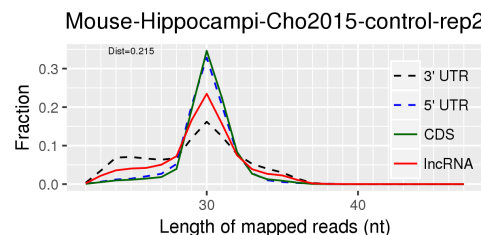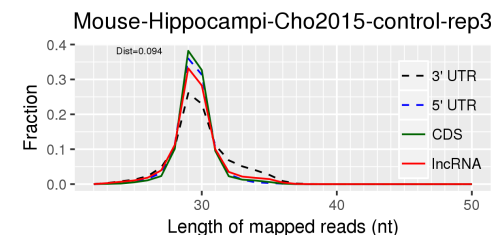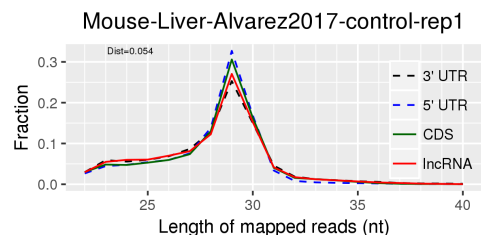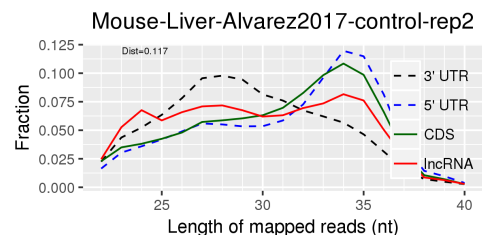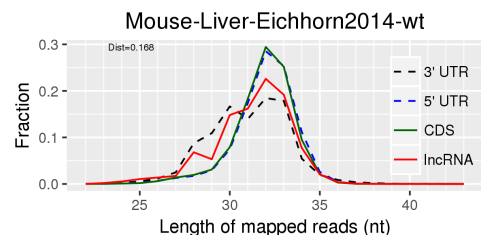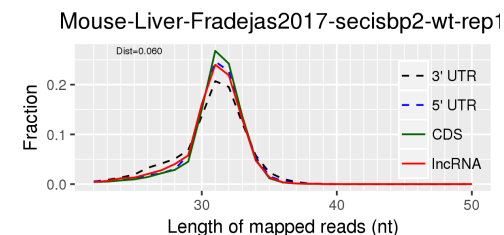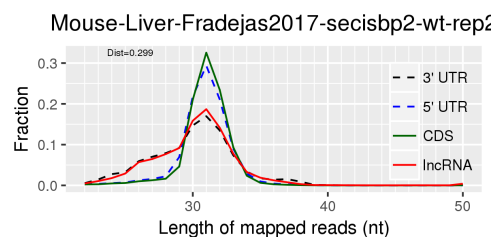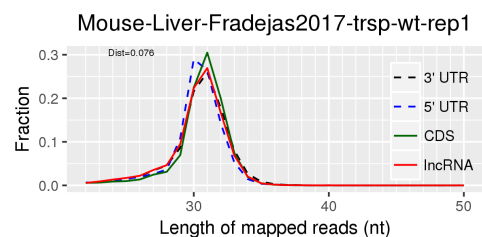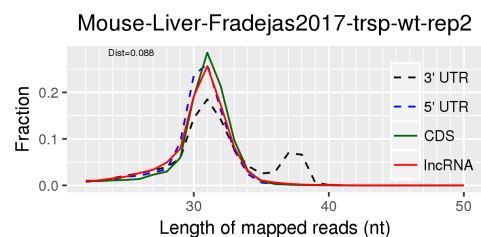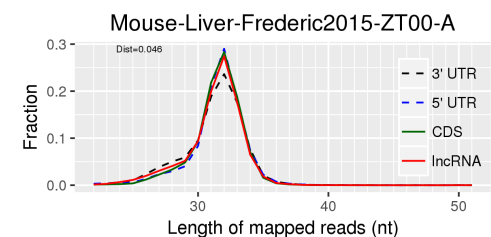

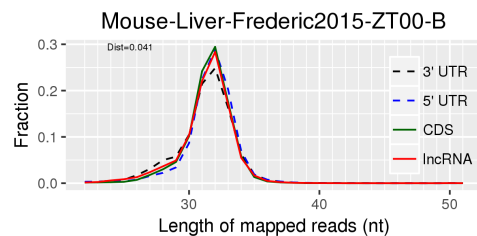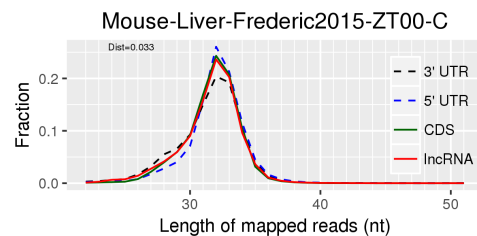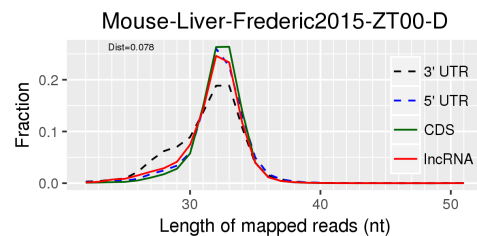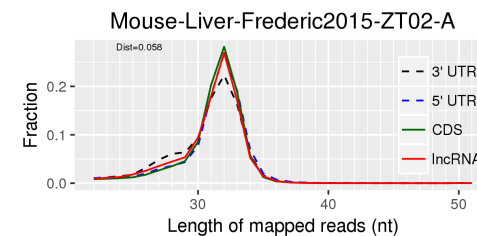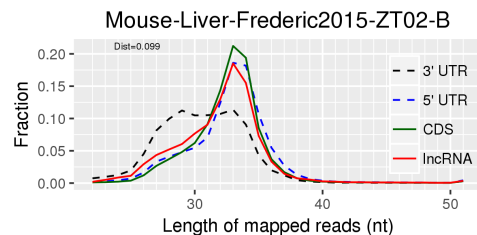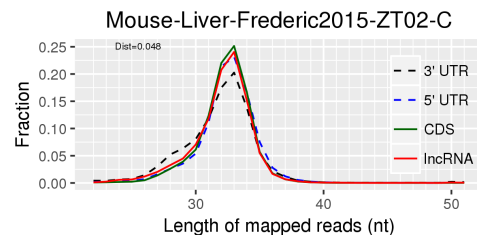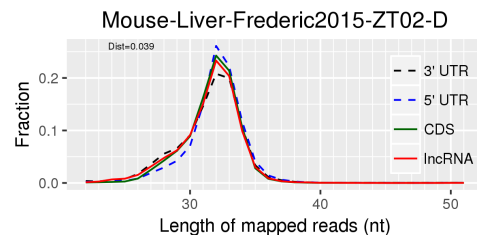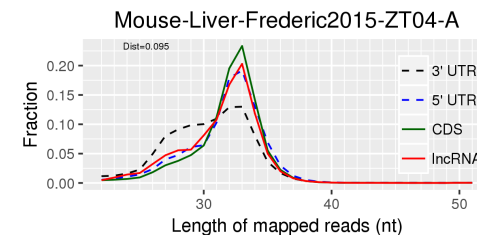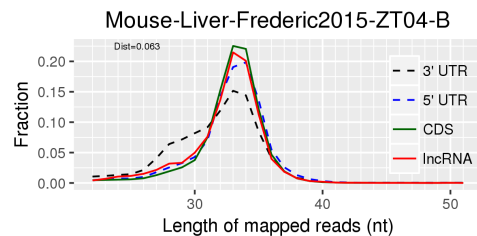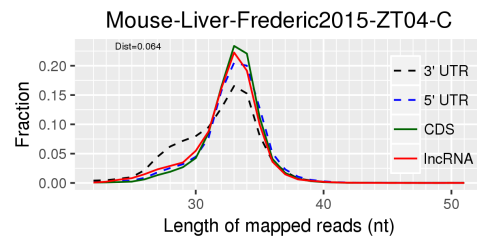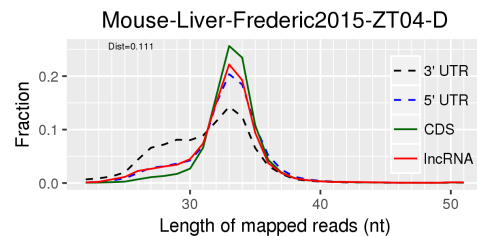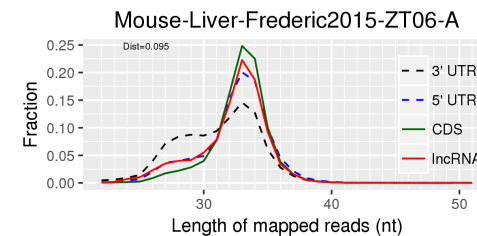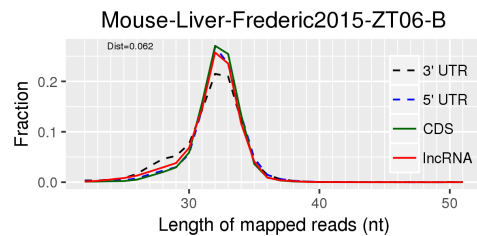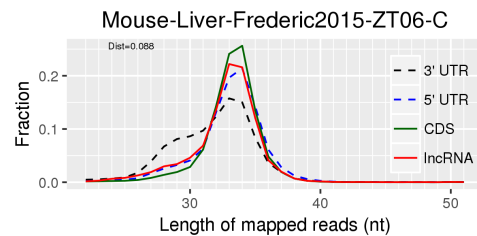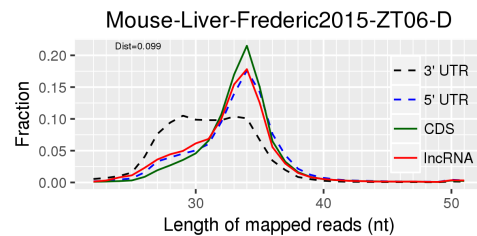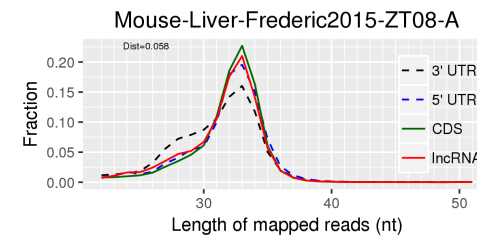

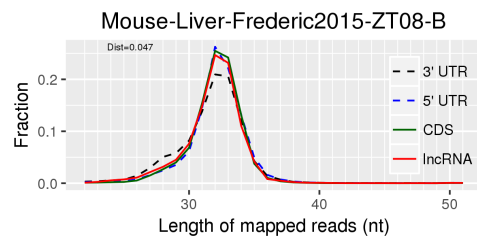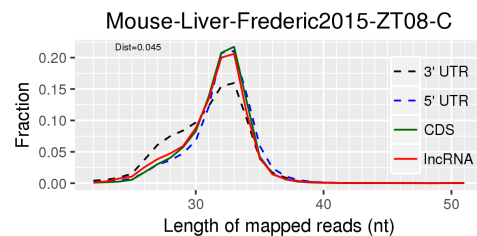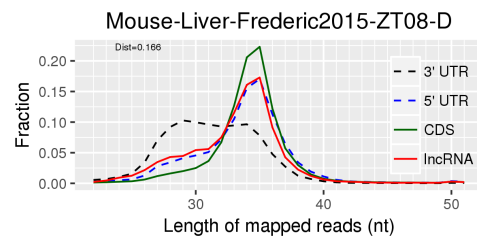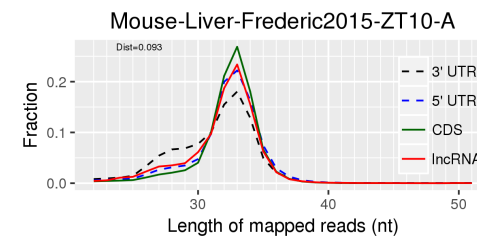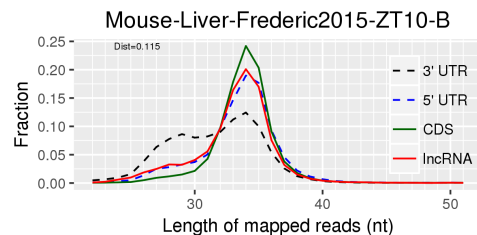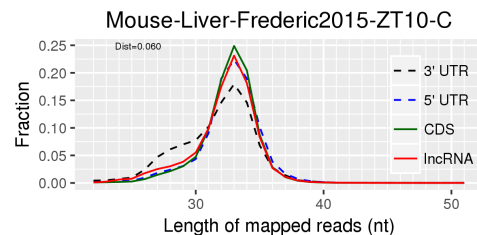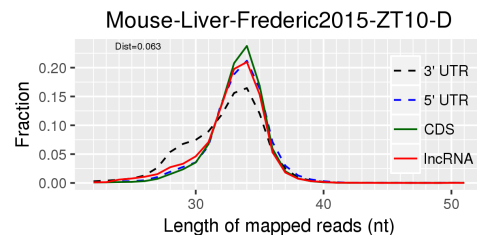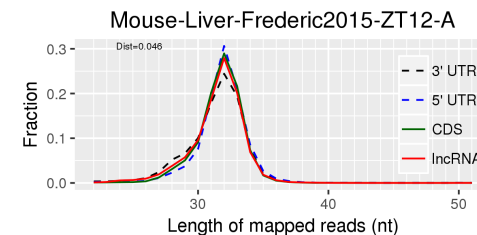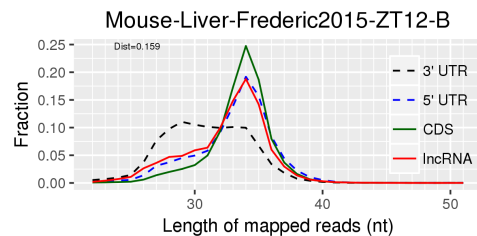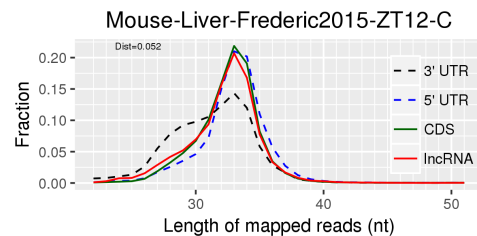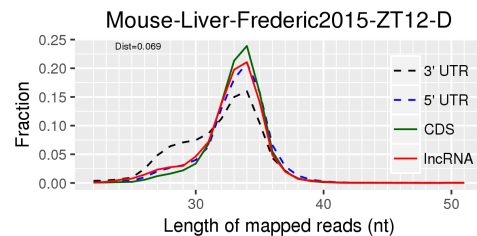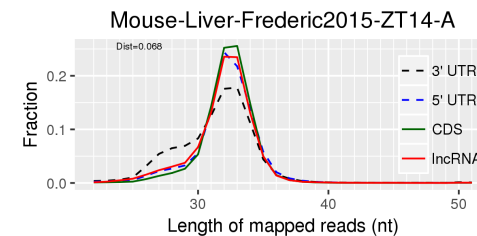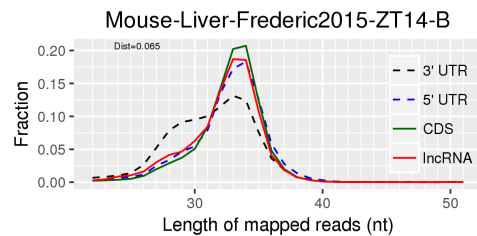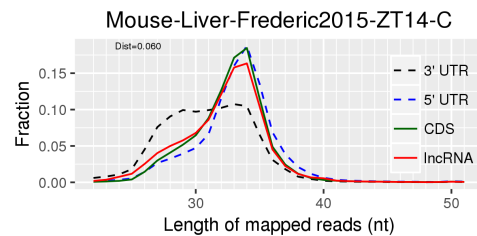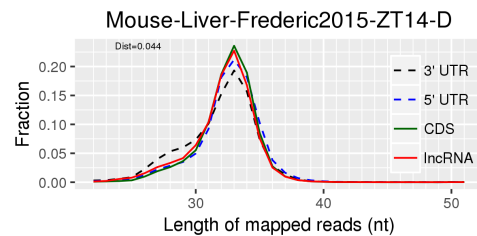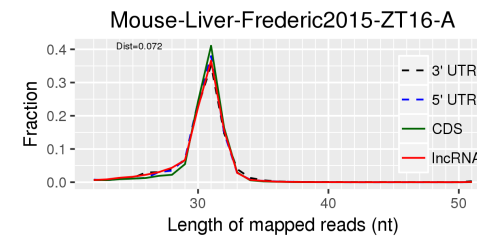

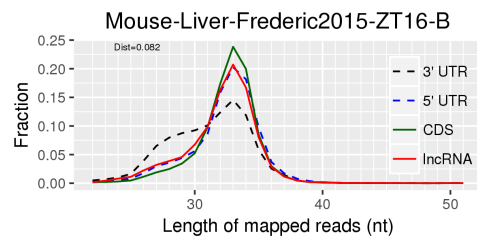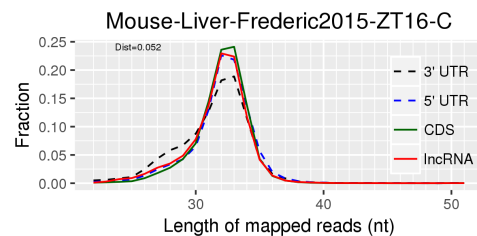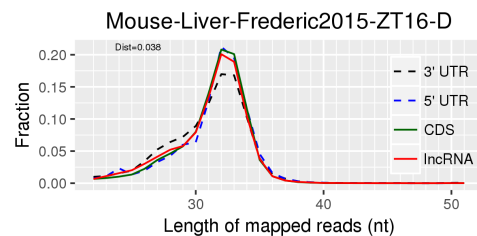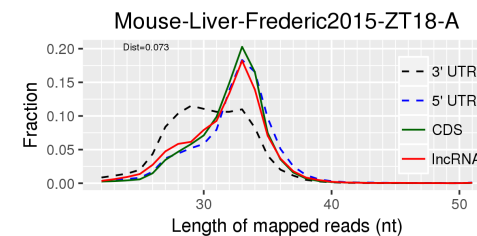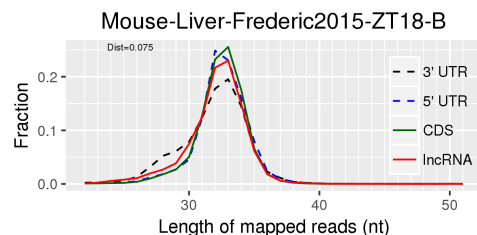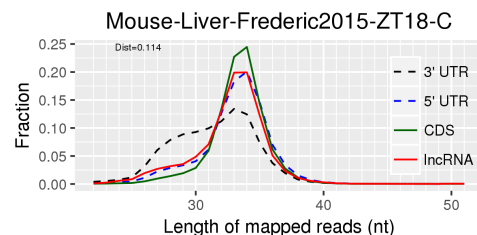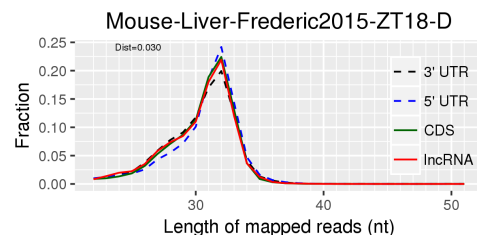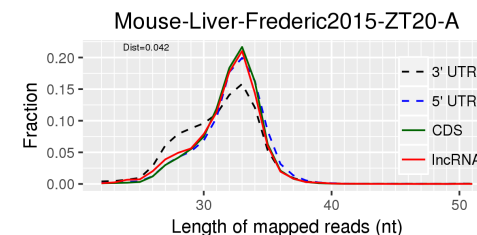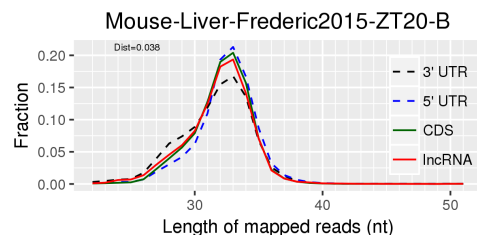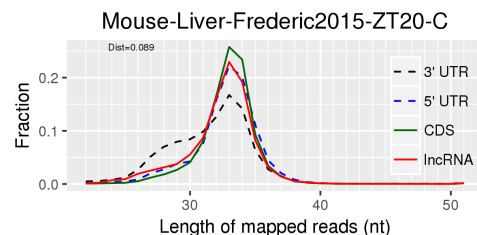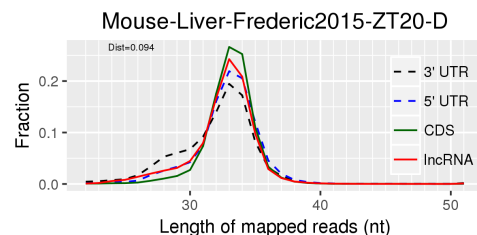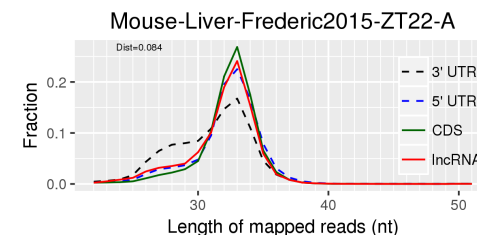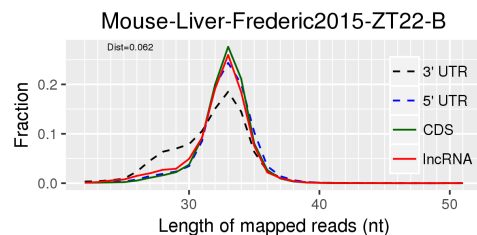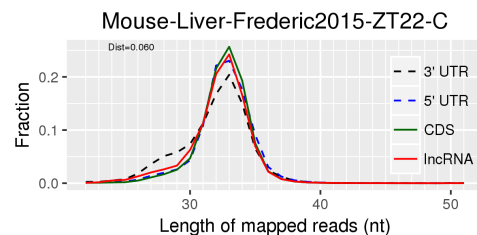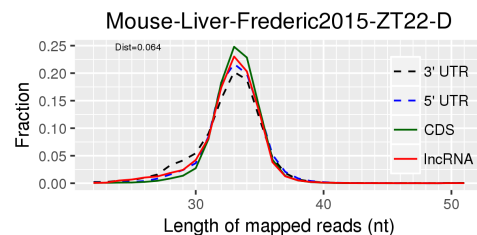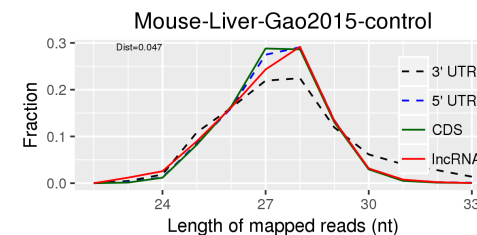

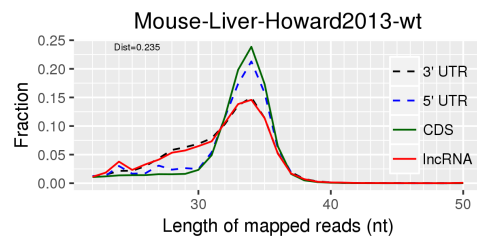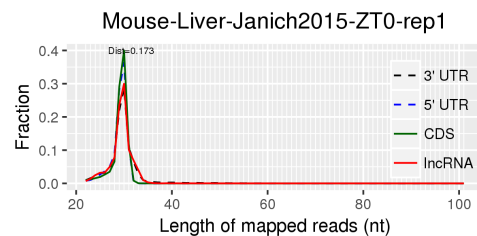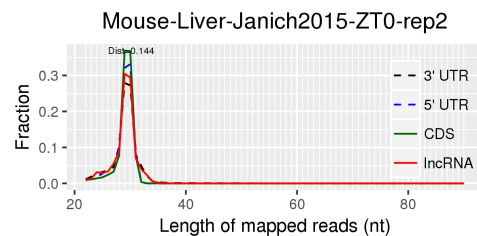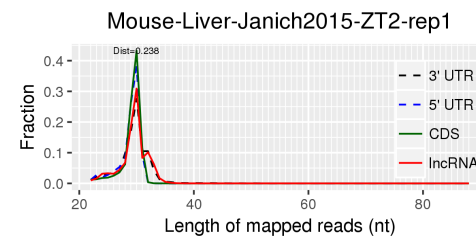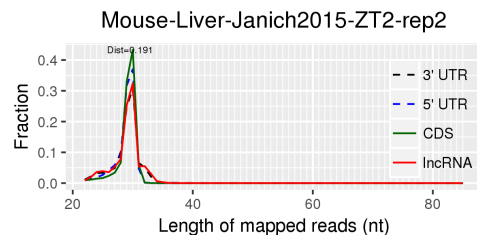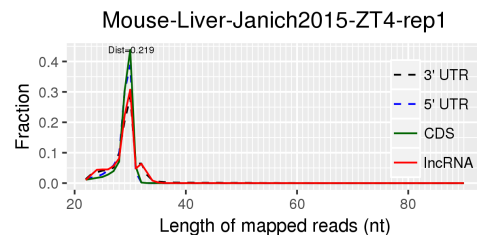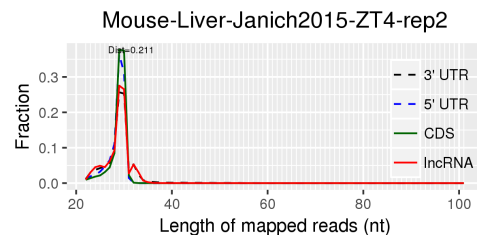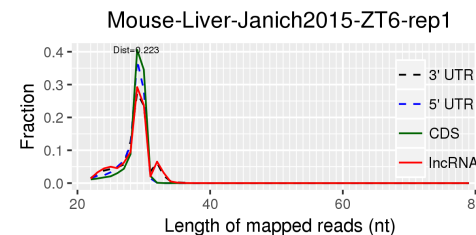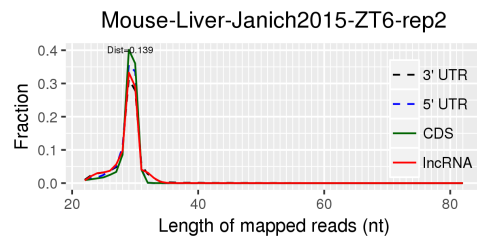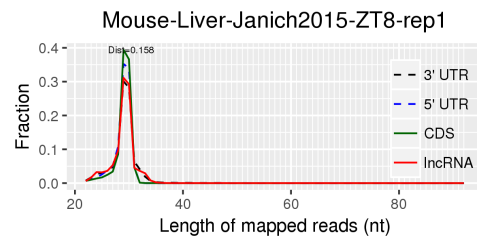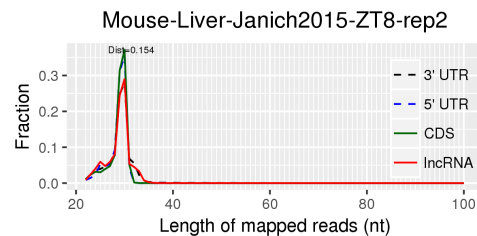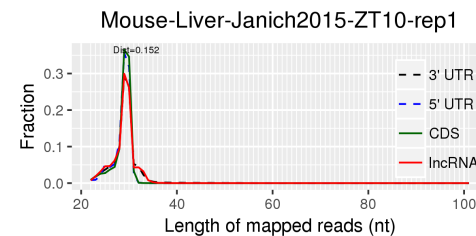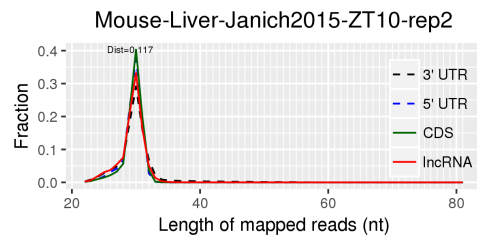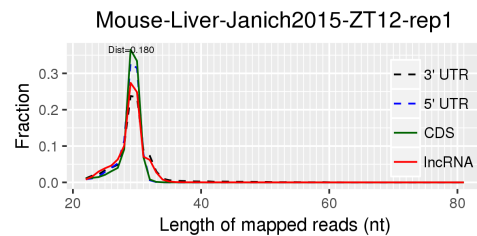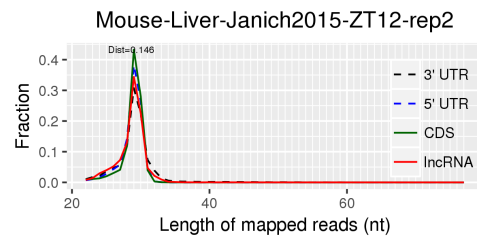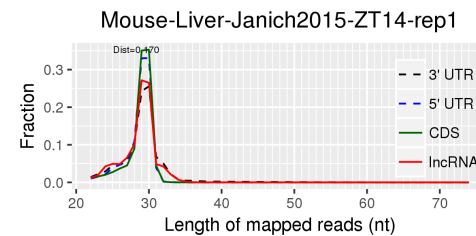

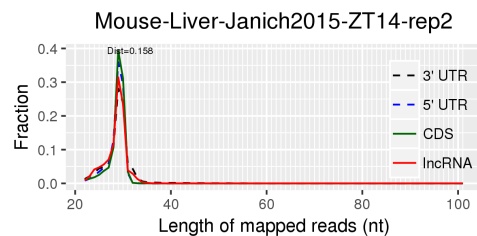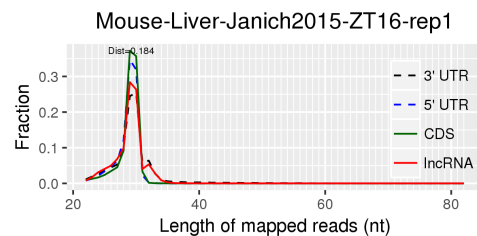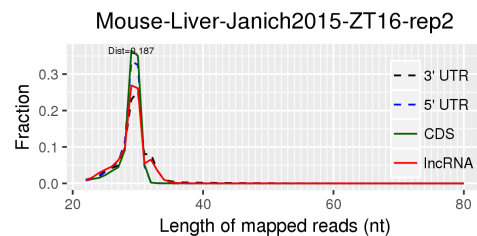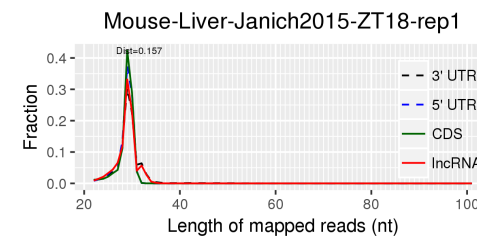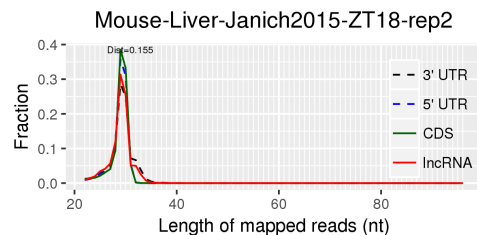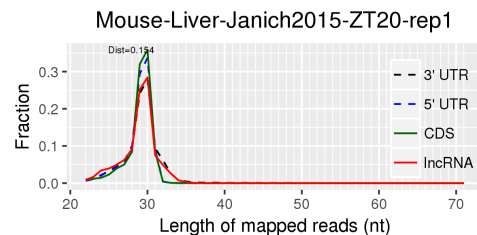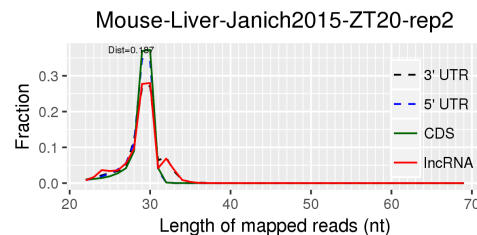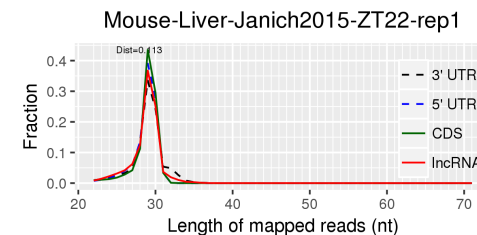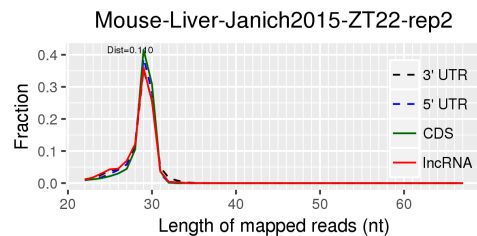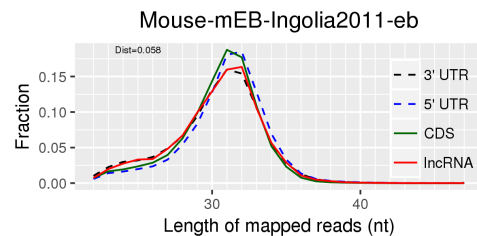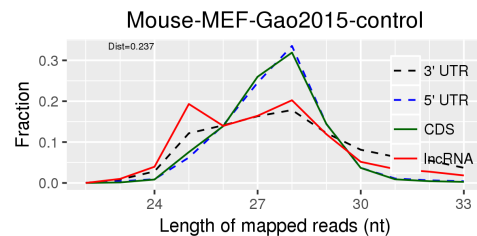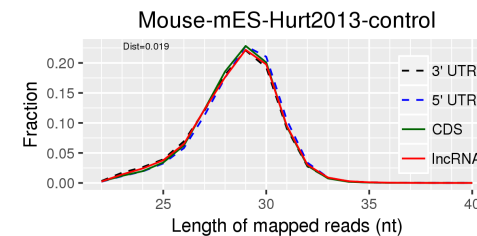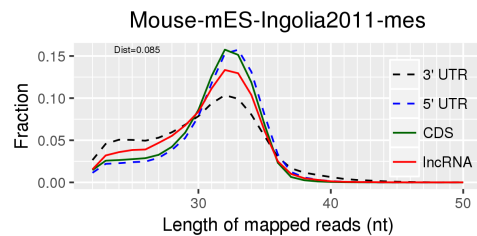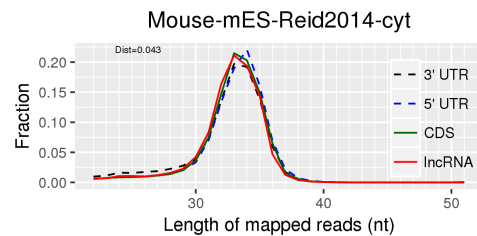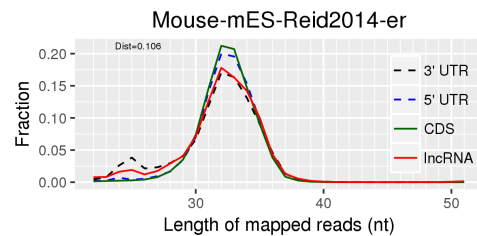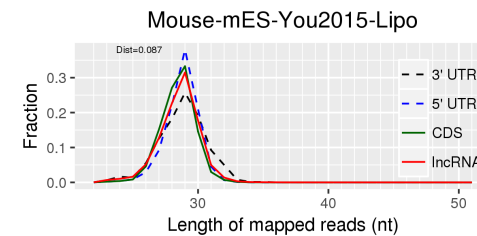

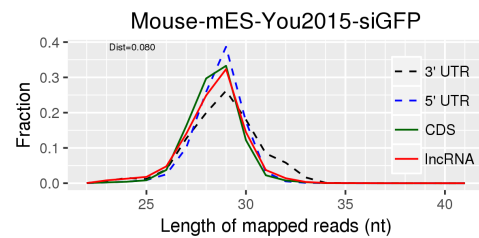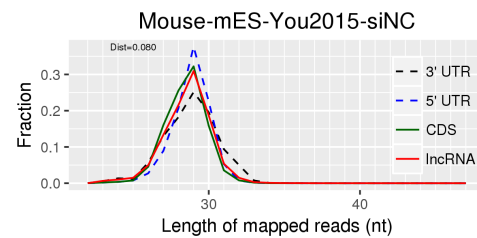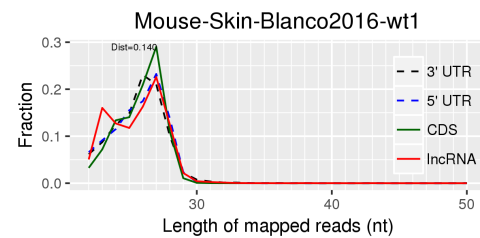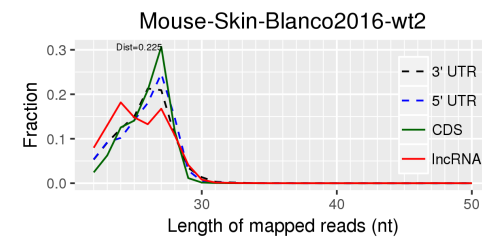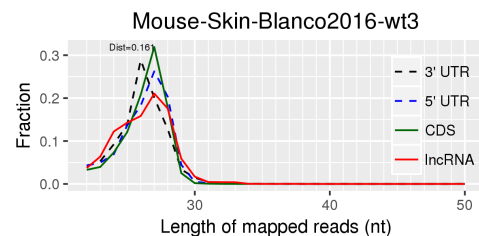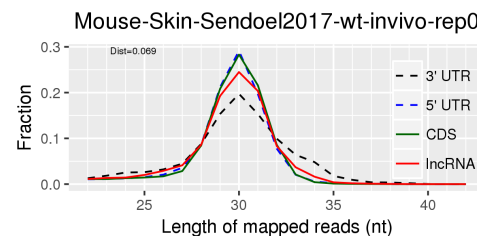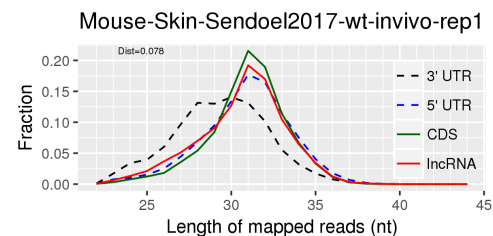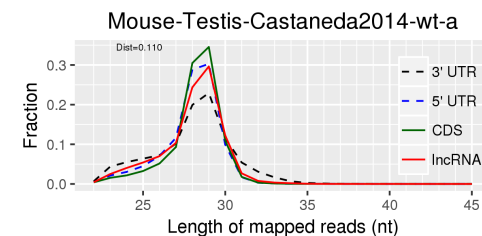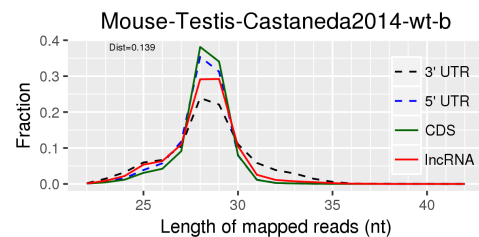

Supplement: Supplementary file 5 — Figure S2. Frequency distributions of Ribo-seq read lengths across CDSs, 5 ′/3 ′UTRs, and lncRNAs (mouse). (PDF 10956.8 kb) [file 12864_2018_4765_MOESM5_ESM.pdf]

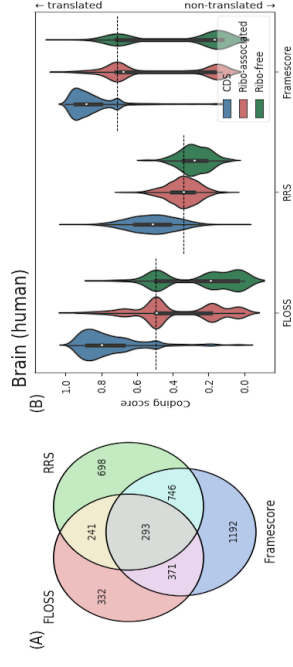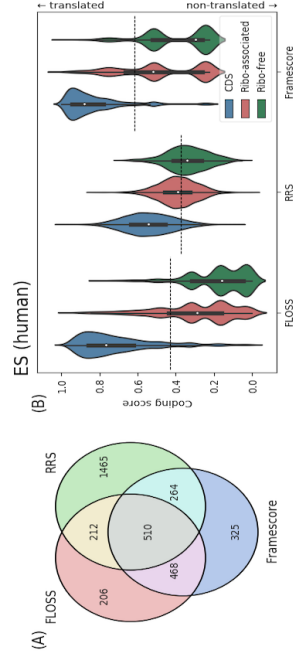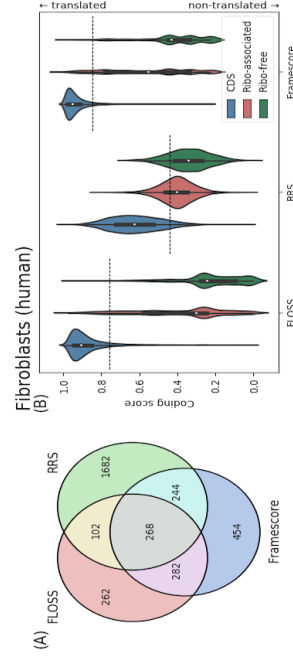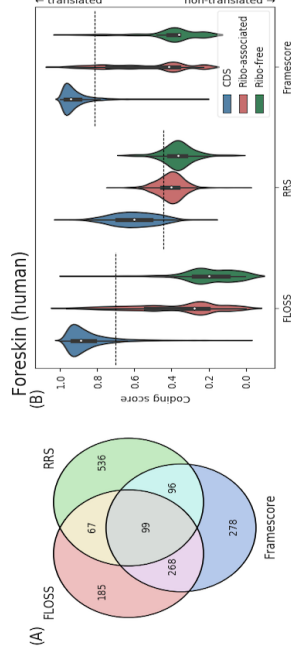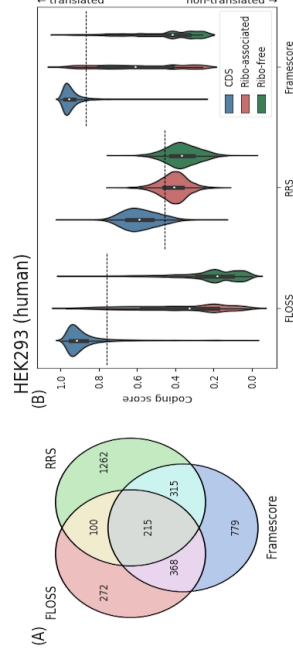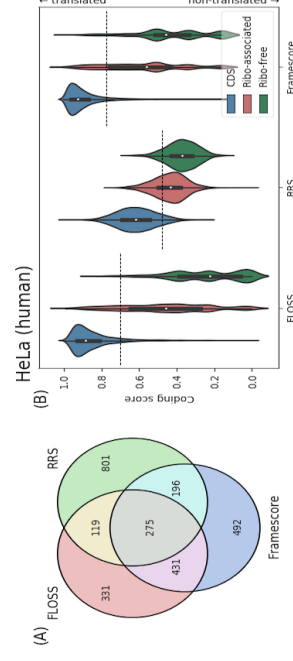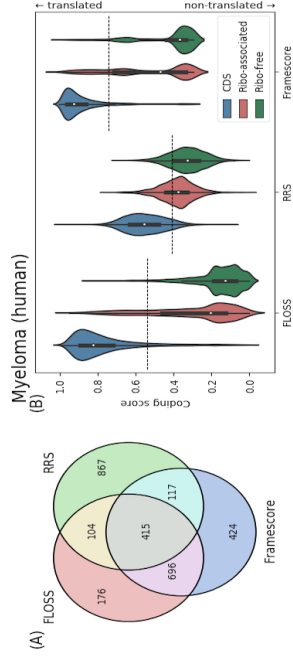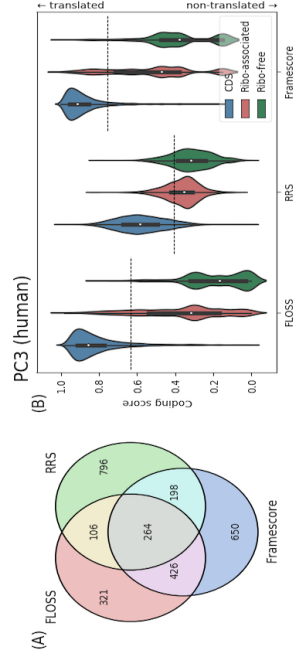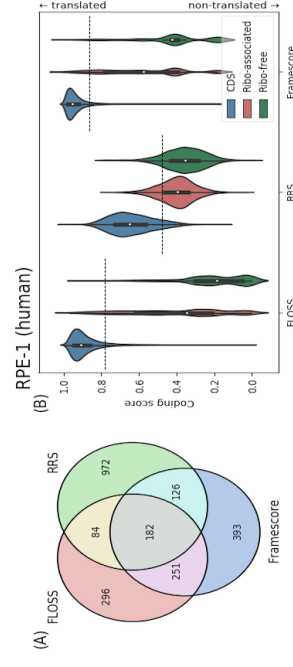

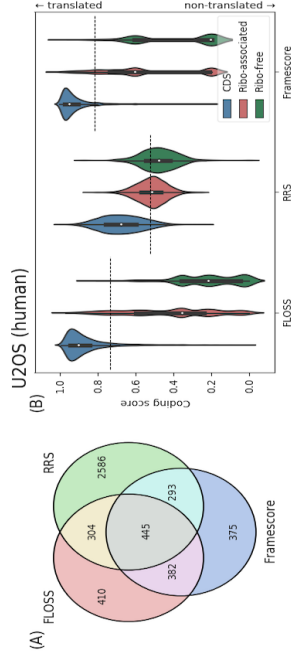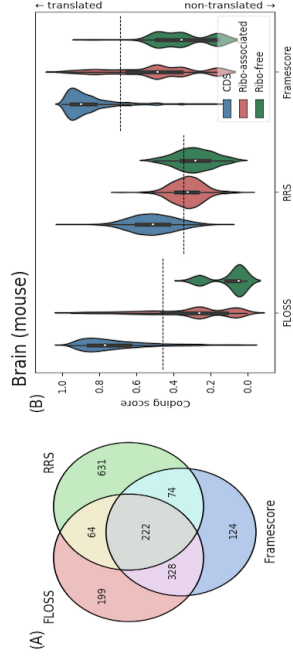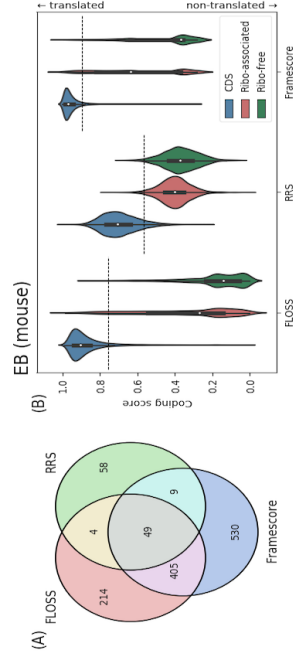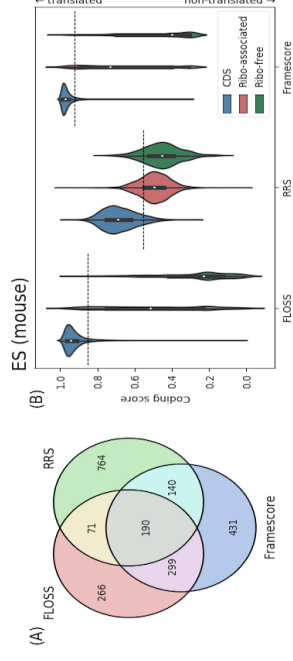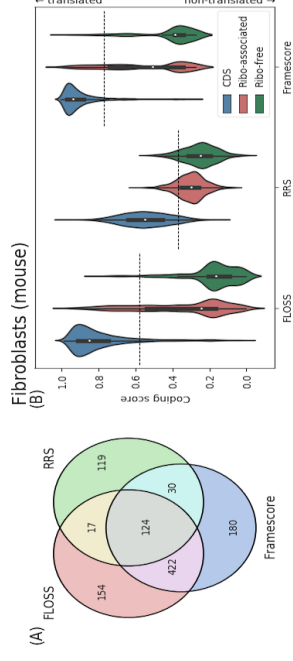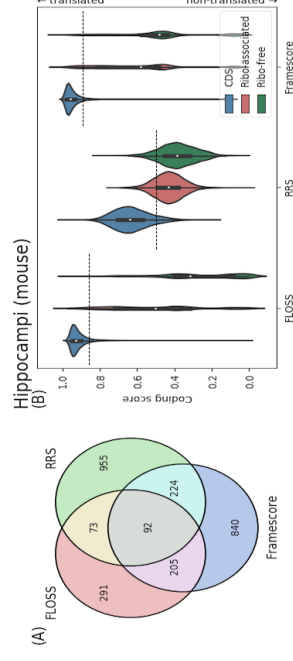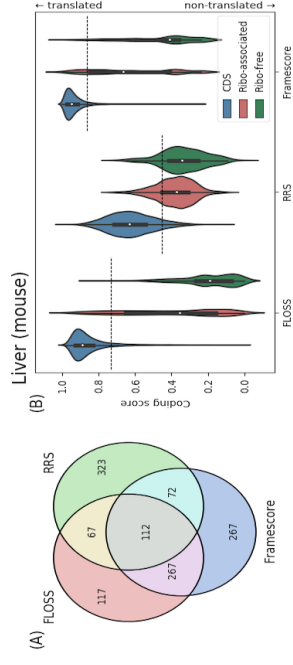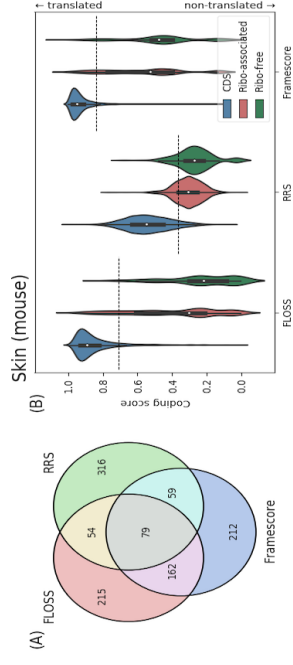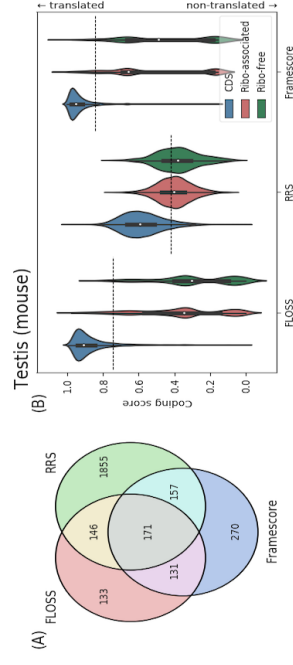

Supplement: Supplementary file 12 — Figure S4. Analysis of coding potential by using FLOSS, RRS, and Framescore in all selected datasets. (PDF 1771.52 kb) [file 12864_2018_4765_MOESM12_ESM.pdf]
