# Supplementary material for: Identification and analysis of ribosome-associated lncRNAs using ribosome profiling data
Source: BMC Genomics. 2018 May 29;19:414. doi: 10.1186/s12864-018-4765-z (PMC5975437; doi:10.1186/s12864-018-4765-z)
Supplement: Supplementary file 6 — Table S3. Genomic sequences, gene annotations, and contaminant sequences for human and mouse. (DOCX 279 kb) [file 12864_2018_4765_MOESM6_ESM.docx]

# Table S3. Genomic sequences, gene annotations and contaminant sequences for human and mouse. Genomic sequences and gene annotation files were downloaded from GENCODE[[1,2]](https://paperpile.com/c/azC5cU/aERAS+7R9Kb). The contaminant sequence collection consists of transfer RNAs (tRNAs), ribosomal RNAs (rRNAs), small nucleolar RNAs (snoRNAs), small nuclear RNAs (snRNAs) and microRNAs (miRNAs). Here, tRNAs were retrieved from GtRNAdb[[3]](https://paperpile.com/c/azC5cU/TQurD), rRNAs were obtained from NCBI[[4]](https://paperpile.com/c/azC5cU/vDWNZ), UCSC[[5]](https://paperpile.com/c/azC5cU/HWJou) and Ensembl[[6]](https://paperpile.com/c/azC5cU/YIe6i).

| **Species** | **Files** | **Descriptions** |
| --- | --- | --- |
| Human | Genome (hg19) | http://hgdownload.cse.ucsc.edu/goldenPath/hg19/bigZips/hg19.2bit |
|  | GTF (GENCODE_v25lift37) | ftp://ftp.sanger.ac.uk/pub/gencode/Gencode_human/release_25/GRCh37_mapping/gencode.v25lift37.annotation.gtf.gz |
|  | tRNAs (GtRNAdb_hg19) | http://gtrnadb.ucsc.edu/genomes/eukaryota/Hsapi19/hg19-tRNAs.fa |
|  | rRNAs (NCBI) | Search “Nucleotide” database by “rRNA[All Fields] AND "Homo sapiens"[porgn] AND biomol_rrna[PROP]” |
|  | rRNAs (UCSC) | Search “Table Browser” by “genome:Human; assembly:Feb.2009 (GRCh37/hg19); group:All tables; table:rsmk; repClass = rRNA” |
|  | rRNA (Ensembl) | Search “BioMart” by “Ensembl Genes 90; Human genes (GRCh38.p10); Transcript type: rRNA” |
|  | snoRNAs/snRNAs/miRNAs | Search “BioMart” by “Ensembl Genes 90; Human genes (GRCh38.p10); Transcript type: snoRNA, snRNA, miRNA” |
| Mouse | Genome (mm10) | http://hgdownload.soe.ucsc.edu/goldenPath/mm10/bigZips/mm10.2bit |
|  | GTF (GENCODE_v12) | ftp://ftp.sanger.ac.uk/pub/gencode/Gencode_mouse/release_M12/gencode.vM12.annotation.gtf.gz |
|  | tRNAs (GtRNAdb_mm10) | http://gtrnadb.ucsc.edu/genomes/eukaryota/Mmusc10/mm10-tRNAs.fa |
|  | rRNAs (NCBI) | Search “Nucleotide” database by “rRNA[All Fields] AND "Mus musculus"[porgn] AND biomol_rrna[PROP]” |
|  | rRNAs (UCSC) | Search “Table Browser” by “genome:Mouse; assembly:Dec.2011 (GRCm38/mm10); group:All tables; table:rsmk; repClass = rRNA” |
|  | rRNA (Ensembl) | Search “BioMart” by “Ensembl Genes 90; Mouse genes (GRCm38.p5); Transcript type: rRNA” |
|  | snoRNAs/snRNAs/miRNAs | Search “BioMart” by “Ensembl Genes 90; Mouse genes (GRCm38.p5); Transcript type: snoRNA, snRNA, miRNA” |

##

**Reference**

[1. Harrow J, Frankish A, Gonzalez JM, Tapanari E, Diekhans M, Kokocinski F, et al. GENCODE: the reference human genome annotation for The ENCODE Project. Genome Res. 2012;22:1760–74. Available from:](http://paperpile.com/b/azC5cU/aERAS) <http://dx.doi.org/10.1101/gr.135350.111>

[2. Mudge JM, Harrow J. Creating reference gene annotation for the mouse C57BL6/J genome assembly. Mamm. Genome 2015;26:366–78. Available from:](http://paperpile.com/b/azC5cU/7R9Kb) <http://dx.doi.org/10.1007/s00335-015-9583-x>

[3. Chan PP, Lowe TM. GtRNAdb: a database of transfer RNA genes detected in genomic sequence. Nucleic Acids Res. 2009;37:D93–7. Available from:](http://paperpile.com/b/azC5cU/TQurD) <http://dx.doi.org/10.1093/nar/gkn787>

[4. NCBI Resource Coordinators. Database Resources of the National Center for Biotechnology Information. Nucleic Acids Res. 2017;45:D12–7. Available from:](http://paperpile.com/b/azC5cU/vDWNZ) <http://dx.doi.org/10.1093/nar/gkw1071>

[5. Karolchik D, Hinrichs AS, Furey TS, Roskin KM, Sugnet CW, Haussler D, et al. The UCSC Table Browser data retrieval tool. Nucleic Acids Res. 2004;32:D493–6. Available from:](http://paperpile.com/b/azC5cU/HWJou) <http://dx.doi.org/10.1093/nar/gkh103>

[6. Aken BL, Ayling S, Barrell D, Clarke L, Curwen V, Fairley S, et al. The Ensembl gene annotation system. Database 2016. Available from:](http://paperpile.com/b/azC5cU/YIe6i) <http://dx.doi.org/10.1093/database/baw093>
