# Supplementary material for: Identification and analysis of ribosome-associated lncRNAs using ribosome profiling data
Source: BMC Genomics. 2018 May 29;19:414. doi: 10.1186/s12864-018-4765-z (PMC5975437; doi:10.1186/s12864-018-4765-z)
Supplement: Supplementary file 7 — Table S4. Software and parameters used in this study. (DOCX 278 kb) [file 12864_2018_4765_MOESM7_ESM.docx]

# Table S4. Softwares and parameters used in this study

| **Softwares** | **Parameters** | **Descriptions** |
| --- | --- | --- |
| Cutadapt v1.9.1 [[1]](https://paperpile.com/c/e5drSD/EXB8F) | -a *ADAPTER* -m 15 | Remove trimmed reads that are shorter than 15nt |
| Bowtie v2.3.2 [[2]](https://paperpile.com/c/e5drSD/47wDk) | --very-sensitive-local | Discard contaminant sequences from RNA-seq data |
|  | --very-sensitive-local -k 100 | Align RNA-seq data to the transcriptome  (up to 100 alignments are allowed for a read) |
|  | --very-sensitive-local --norc | Discard contaminant sequences from Ribo-seq data  (only forward reference strand is considered) |
|  | --very-sensitive-local -k 100 --norc  --rdg 99999999,99999999 --rfg 99999999,99999999 | Align Ribo-seq data to the transcriptome  (insertion and deletion are not allowed) |
| RSEM v1.2.31 [[3]](https://paperpile.com/c/e5drSD/2d7kA) | rsem-calculate-expression --alignments | Estimate transcript expression from SAM file |

#

**Reference**

[1. Martin M. Cutadapt removes adapter sequences from high-throughput sequencing reads. EMBnet.journal 2011 [cited 2017 Aug 29];17:10–2. Available from:](http://paperpile.com/b/e5drSD/EXB8F) <http://journal.embnet.org/index.php/embnetjournal/article/view/200>

[2. Langmead B, Salzberg SL. Fast gapped-read alignment with Bowtie 2. Nat. Methods 2012;9:357–9. Available from:](http://paperpile.com/b/e5drSD/47wDk) <http://dx.doi.org/10.1038/nmeth.1923>

[3. Li B, Dewey CN. RSEM: accurate transcript quantification from RNA-Seq data with or without a reference genome. BMC Bioinformatics 2011;12:323. Available from:](http://paperpile.com/b/e5drSD/2d7kA) <http://dx.doi.org/10.1186/1471-2105-12-323>
