# Supplementary material for: Identification and analysis of ribosome-associated lncRNAs using ribosome profiling data
Source: BMC Genomics. 2018 May 29;19:414. doi: 10.1186/s12864-018-4765-z (PMC5975437; doi:10.1186/s12864-018-4765-z)
Supplement: Supplementary file 9 — Figure S3. The discrimination of ribosome-associated and ribosome-free lncRNAs by ribosome density in all selected datasets. (PDF 1146.88 kb) [file 12864_2018_4765_MOESM9_ESM.pdf]

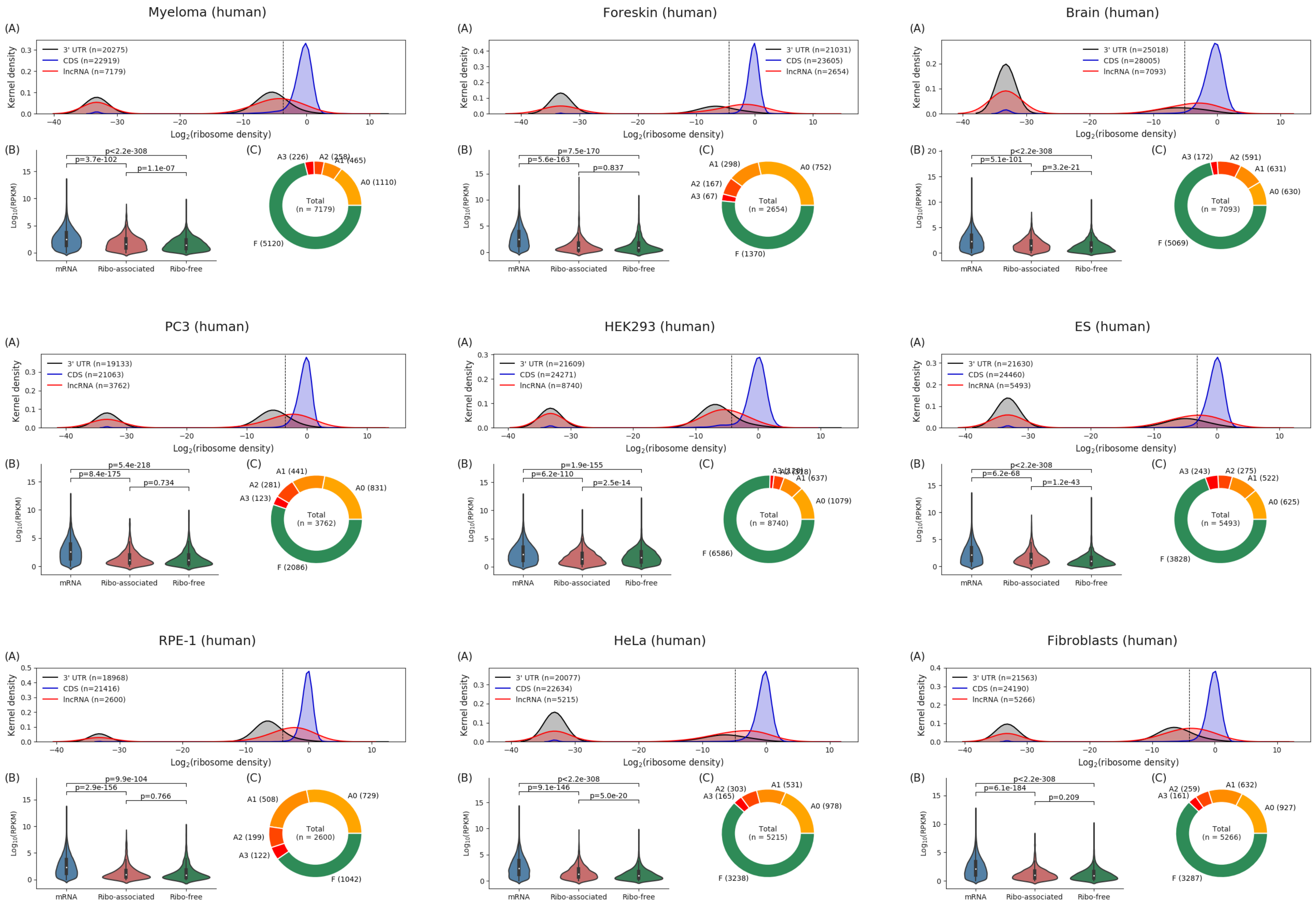
Myeloma (human)
(A)
Kernel density
3' UTR (n=20275)
CDS (n=22919)
lncRNA (n=7179)
Log2(ribosome density)
(B)
Log10(RPKM)
p<2.2e-308
p=3.7e-102
p=1.1e-07
mRNA
Ribo-associated
Ribo-free
(C)
A3 (226)
A2 (258)
A1 (465)
A0 (1110)
Total (n = 7179)
F (5120)
Foreskin (human)
(A)
Kernel density
3' UTR (n=21031)
CDS (n=23605)
lncRNA (n=2654)
Log2(ribosome density)
(B)
Log10(RPKM)
p=7.5e-170
p=5.6e-163
p=0.837
mRNA
Ribo-associated
Ribo-free
(C)
A1 (298)
A0 (752)
A2 (167)
A3 (67)
Total (n = 2654)
F (1370)
Brain (human)
(A)
Kernel density
3' UTR (n=25018)
CDS (n=28005)
lncRNA (n=7093)
Log2(ribosome density)
(B)
Log10(RPKM)
p<2.2e-308
p=5.1e-101
p=3.2e-21
mRNA
Ribo-associated
Ribo-free
(C)
A3 (172)
A2 (591)
A1 (631)
A0 (630)
Total (n = 7093)
F (5069)
PC3 (human)
(A)
Kernel density
3' UTR (n=19133)
CDS (n=21063)
lncRNA (n=3762)
Log2(ribosome density)
(B)
Log10(RPKM)
p=5.4e-218
p=8.4e-175
p=0.734
mRNA
Ribo-associated
Ribo-free
(C)
A1 (441)
A2 (281)
A3 (123)
A0 (831)
Total (n = 3762)
F (2086)
HEK293 (human)
(A)
Kernel density
3' UTR (n=21609)
CDS (n=24271)
lncRNA (n=8740)
Log2(ribosome density)
(B)
Log10(RPKM)
p=1.9e-155
p=6.2e-110
p=2.5e-14
mRNA
Ribo-associated
Ribo-free
(C)
A1 (637)
A0 (1079)
Total (n = 8740)
F (6586)
ES (human)
(A)
Kernel density
3' UTR (n=21630)
CDS (n=24460)
lncRNA (n=5493)
Log2(ribosome density)
(B)
Log10(RPKM)
p<2.2e-308
p=6.2e-68
p=1.2e-43
mRNA
Ribo-associated
Ribo-free
(C)
A3 (243)
A2 (275)
A1 (522)
A0 (625)
Total (n = 5493)
F (3828)
RPE-1 (human)
(A)
Kernel density
3' UTR (n=18968)
CDS (n=21416)
lncRNA (n=2600)
Log2(ribosome density)
(B)
Log10(RPKM)
p=9.9e-104
p=2.9e-156
p=0.766
mRNA
Ribo-associated
Ribo-free
(C)
A1 (508)
A0 (729)
A2 (199)
A3 (122)
Total (n = 2600)
F (1042)
HeLa (human)
(A)
Kernel density
3' UTR (n=20077)
CDS (n=22634)
lncRNA (n=5215)
Log2(ribosome density)
(B)
Log10(RPKM)
p<2.2e-308
p=9.1e-146
p=5.0e-20
mRNA
Ribo-associated
Ribo-free
(C)
A2 (303)
A3 (165)
A1 (531)
A0 (978)
Total (n = 5215)
F (3238)
Fibroblasts (human)
(A)
Kernel density
3' UTR (n=21563)
CDS (n=24190)
lncRNA (n=5266)
Log2(ribosome density)
(B)
Log10(RPKM)
p<2.2e-308
p=6.1e-184
p=0.209
mRNA
Ribo-associated
Ribo-free
(C)
A2 (259)
A3 (161)
A1 (632)
A0 (927)
Total (n = 5266)
F (3287)

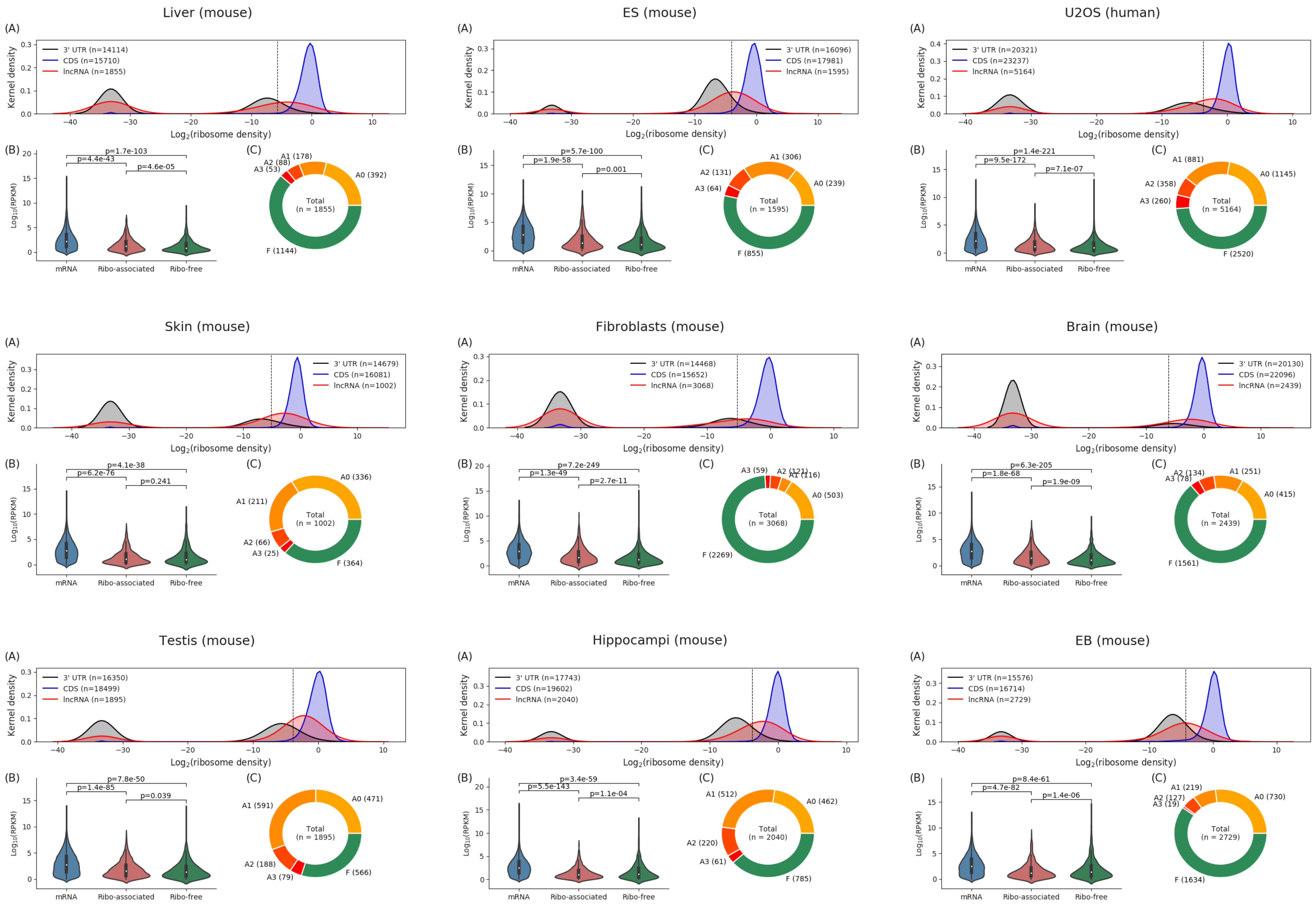

Liver (mouse)
(A)
Kernel density
3' UTR (n=14114)
CDS (n=15710)
lncRNA (n=1855)
Log2(ribosome density)
(B)
Log10(RPKM)
p=1.7e-103
p=4.4e-43
p=4.6e-05
mRNA
Ribo-associated
Ribo-free
(C)
A1 (178)
A2 (88)
A3 (53)
A0 (392)
Total
(n = 1855)
F (1144)
ES (mouse)
(A)
Kernel density
3' UTR (n=16096)
CDS (n=17981)
lncRNA (n=1595)
Log2(ribosome density)
(B)
Log10(RPKM)
p=5.7e-100
p=1.9e-58
p=0.001
mRNA
Ribo-associated
Ribo-free
(C)
A1 (306)
A2 (131)
A3 (64)
A0 (239)
Total
(n = 1595)
F (855)
U2OS (human)
(A)
Kernel density
3' UTR (n=20321)
CDS (n=23237)
lncRNA (n=5164)
Log2(ribosome density)
(B)
Log10(RPKM)
p=1.4e-221
p=9.5e-172
p=7.1e-07
mRNA
Ribo-associated
Ribo-free
(C)
A1 (881)
A2 (358)
A3 (260)
A0 (1145)
Total
(n = 5164)
F (2520)
Skin (mouse)
(A)
Kernel density
3' UTR (n=14679)
CDS (n=16081)
lncRNA (n=1002)
Log2(ribosome density)
(B)
Log10(RPKM)
p=4.1e-38
p=6.2e-76
p=0.241
mRNA
Ribo-associated
Ribo-free
(C)
A0 (336)
A1 (211)
A2 (66)
A3 (25)
Total
(n = 1002)
F (364)
Fibroblasts (mouse)
(A)
Kernel density
3' UTR (n=14468)
CDS (n=15652)
lncRNA (n=3068)
Log2(ribosome density)
(B)
Log10(RPKM)
p=7.2e-249
p=1.3e-49
p=2.7e-11
mRNA
Ribo-associated
Ribo-free
(C)
A3 (59)
A2 (121)
A1 (116)
A0 (503)
Total
(n = 3068)
F (2269)
Brain (mouse)
(A)
Kernel density
3' UTR (n=20130)
CDS (n=22096)
lncRNA (n=2439)
Log2(ribosome density)
(B)
Log10(RPKM)
p=6.3e-205
p=1.8e-68
p=1.9e-09
mRNA
Ribo-associated
Ribo-free
(C)
A2 (134)
A3 (78)
A1 (251)
A0 (415)
Total
(n = 2439)
F (1561)
Testis (mouse)
(A)
Kernel density
3' UTR (n=16350)
CDS (n=18499)
lncRNA (n=1895)
Log2(ribosome density)
(B)
Log10(RPKM)
p=7.8e-50
p=1.4e-85
p=0.039
mRNA
Ribo-associated
Ribo-free
(C)
A1 (591)
A0 (471)
A2 (188)
A3 (79)
Total
(n = 1895)
F (566)
Hippocampi (mouse)
(A)
Kernel density
3' UTR (n=17743)
CDS (n=19602)
lncRNA (n=2040)
Log2(ribosome density)
(B)
Log10(RPKM)
p=3.4e-59
p=5.5e-143
p=1.1e-04
mRNA
Ribo-associated
Ribo-free
(C)
A1 (512)
A0 (462)
A2 (220)
A3 (61)
Total
(n = 2040)
F (785)
EB (mouse)
(A)
Kernel density
3' UTR (n=15576)
CDS (n=16714)
lncRNA (n=2729)
Log2(ribosome density)
(B)
Log10(RPKM)
p=8.4e-61
p=4.7e-82
p=1.4e-06
mRNA
Ribo-associated
Ribo-free
(C)
A1 (219)
A2 (127)
A3 (19)
A0 (730)
Total
(n = 2729)
F (1634)
