# Supplementary material for: Identification and analysis of ribosome-associated lncRNAs using ribosome profiling data
Source: BMC Genomics. 2018 May 29;19:414. doi: 10.1186/s12864-018-4765-z (PMC5975437; doi:10.1186/s12864-018-4765-z)
Supplement: Supplementary file 11 — Table S5. Contaminant Ribo-seq reads derived from miRNAs, snRNAs, and snoRNAs are enriched in lncRNAs. (DOCX 280 kb) [file 12864_2018_4765_MOESM11_ESM.docx]

# Table S5. Contaminant Ribo-seq reads derived from miRNAs, snRNAs and snoRNAs are enriched in lncRNAs

|  | **Length of Ribo-seq read (nt)** | | | | | | | | | |
| --- | --- | --- | --- | --- | --- | --- | --- | --- | --- | --- |
| **Dataset** | **18** | **21** | **22** | **23** | **24** | **25** | **28** | **29** | **30** | **33** |
| Human-Brain-Gonzalez2014-normal-C | mir-4286 |  |  |  |  |  |  |  |  |  |
| Human-Brain-Gonzalez2014-tumor-A | mir-4286 |  |  |  |  |  |  |  |  |  |
| Human-Breast-Rubio2014-control-rep1 |  | U2 snRNA |  |  |  |  | snoRNA |  |  |  |
| Human-Breast-Rubio2014-control-rep2 |  |  |  |  |  |  | snoRNA |  |  |  |
| Human-Fibroblasts-Shitrit2015-control |  |  |  |  |  |  |  |  |  |  |
| Human-Fibroblasts-Xu2016-wt-d-leucine |  |  |  |  |  | U2 snRNA |  | U11 snRNA | U6/U11 |  |
| Human-Fibroblasts-Xu2016-wt-l-leucine |  |  |  |  |  | U2 snRNA |  | U11 snRNA | U6/U11 |  |
| Human-HEK-Eichhorn2014-mock |  |  |  |  |  |  |  |  |  |  |
| Human-HEK-Iwasaki2016-dmso-rep1 | mir-4286 |  |  |  |  |  |  |  |  |  |
| Human-HEK-Iwasaki2016-dmso-rep2 | mir-4286 |  | U6 snRNA | U6 snRNA |  |  |  |  |  |  |
| Human-HEK-Sidrauski2015-control-b |  |  |  |  | U1 snRNA |  |  |  |  |  |
| Human-HEK-Subtelny2014-cyt |  |  |  |  |  |  |  |  |  |  |
| Human-HeLa-Guo2010-mock12hr |  | U2 snRNA |  |  |  |  |  |  |  |  |
| Human-HeLa-Guo2010-mock32hr |  | U2 snRNA |  |  |  |  |  |  |  |  |
| Human-HeLa-Park2016-Mphase-rep1 |  |  |  | U1 snRNA |  |  |  |  |  |  |
| Human-HeLa-Park2016-Sphase-rep1 |  |  |  |  |  |  |  |  |  |  |
| Human-HeLa-Zur2016-G1phase-exp1 |  | U2 snRNA |  |  |  |  |  |  |  |  |
| Human-HeLa-Zur2016-G1phase-exp2 |  | U2 snRNA |  |  |  |  |  |  |  |  |
| Human-HeLa-Zur2016-Mphase-exp1 |  |  |  |  |  |  |  |  |  |  |
| Human-HeLa-Zur2016-Mphase-exp2 |  | U2 snRNA |  |  |  |  |  |  |  |  |
| Human-hES-Werner2015-control-rep1 |  |  |  |  |  |  |  |  |  |  |
| Human-hES-Werner2015-control-rep2 |  |  |  |  |  |  |  |  |  |  |
| Human-KOPT-K1-Wolfe2014-dmso-rep1 | mir-4286 |  |  | U1 snRNA |  |  |  | U1 snRNA |  | U2 snRNA |
| Human-KOPT-K1-Wolfe2014-dmso-rep2 | mir-4286 |  |  | U1 snRNA |  |  |  | U1 snRNA |  | U2 snRNA |
| Human-Macrophages-Su2015-mock-rep1 |  | U2 snRNA |  |  |  |  |  |  | U2 snRNA |  |
| Human-Macrophages-Su2015-mock-rep2 |  | U2 snRNA |  |  |  |  |  |  | U2 snRNA |  |
| Human-Eye-Tanenbaum2015-G1-rep1 |  | U2 snRNA |  |  |  |  | snoRNA | U1 snRNA |  |  |
| Human-Eye-Tanenbaum2015-G1-rep2 |  | U2 snRNA |  |  |  |  |  | U1 snRNA |  |  |
| Human-Eye-Tanenbaum2015-G2-rep1 |  | U2 snRNA |  |  |  |  |  | U1 snRNA |  | U2 snRNA |
| Human-Eye-Tanenbaum2015-G2-rep2 |  | U2 snRNA |  |  |  |  |  | U1 snRNA |  | U2 snRNA |
| Human-Eye-Tanenbaum2015-M-rep1 |  | U2 snRNA |  |  |  |  | snoRNA | U1 snRNA |  |  |
| Human-Eye-Tanenbaum2025-M-rep2 |  | U2 snRNA |  |  |  |  |  | U1 snRNA |  | U2 snRNA |

# 
