# Supplementary material for: Identification and analysis of ribosome-associated lncRNAs using ribosome profiling data
Source: BMC Genomics. 2018 May 29;19:414. doi: 10.1186/s12864-018-4765-z (PMC5975437; doi:10.1186/s12864-018-4765-z)
Supplement: Supplementary file 17 — Table S13. LncRNAs derived from snoRNA host genes are enriched in trans-lncRNAs and ribo-lncRNAs. (PDF 55.9 kb) [file 12864_2018_4765_MOESM17_ESM.pdf]

	Human		Mouse	
	**#Total**	**#snoRNA host (odds ratio)**	**#Total**	**#snoRNA host (odds ratio)**
**trans-lncRNA**	78	*** 22 (20.46)	42	** 5 (7.26)
**ribo-lncRNA**	613	*** 70 (10.71)	367	*** 44 (11.21)
**noribo-lncRNA**	746	5 (0.42)	326	*** 57 (20.75)
**other**	12209	112 (0.14)	5525	1 (0.00)
**Total**	13646	209	6260	107

(One-sided fisher's exact test) *: p<0.05, **: p<0.01, ***: p<0.001
